# Single‐Cell and Spatial Transcriptomics Uncover Immune Dynamics and Cellular Heterogeneity in Benign Prostatic Hyperplasia and Prostate Cancer Transition

**DOI:** 10.1002/mco2.70760

**Published:** 2026-05-23

**Authors:** Yuanyuan Luo, Haitao Zhong, Tongrui Shang, Bin Hu, Dongbo Yuan, Xueyuan Jia, Ruichong Lin, Zehua Wang, Yinyi Fang, Guohua Zhu, Jukun Song, Zhangcheng Liu, Bo Yan, Fa Sun, Zhenyu Jia, Yunfang Yu, Luhui Mao, Hai Huang, Jianguo Zhu

**Affiliations:** ^1^ Department of Urology Guizhou Provincial People's Hospital Guiyang Guizhou China; ^2^ GuiZhou University Medical College Guiyang Guizhou Province China; ^3^ Guangdong Provincial Key Laboratory of Malignant Tumor Epigenetics and Gene Regulation Department of Medical Oncology Sun Yat‐sen Memorial Hospital Sun Yat‐sen University Guangzhou China; ^4^ Department of Urology Kweichow Moutai Hospital Renhuai Guizhou China; ^5^ School of Pharmacy Faculty of Medicine State Key Laboratory of Quality Research in Chinese Medicines Macau University of Science and Technology Taipa Macau China; ^6^ Faculty of Innovation Engineering Macau University of Science and Technology Taipa Macao China; ^7^ School of Computer and Information Engineering Guangzhou Huali College Guangzhou China; ^8^ Department of Stomatology Guizhou Medical University Stomatological Hospital Guiyang Guizhou China; ^9^ Department of Urology The Second People's Hospital of Neijiang Neijiang Sichuan China; ^10^ Department of Plant and Botany Sciences University of California of Riverside Riverside California USA; ^11^ Faculty of Medicine and Artificial Intelligence Cross Disciplinary Research Institute Macau University of Science and Technology Taipa Macau China; ^12^ Institute of Health Medicine Southern University of Science and Technology Guangzhou China; ^13^ Guangdong Provincial Key Laboratory of Cancer Pathogenesis and Precision Diagnosis and Treatment AI Big Data Laboratory Shenshan Medical Center Memorial Hospital of Sun Yat‐Sen University Shanwei China; ^14^ Key Laboratory for Cancer Prevention and Treatment of Guizhou Province Guiyang Guizhou China

**Keywords:** benign prostatic hyperplasia, continuous transformation map, immune microenvironment, prostate cancer, single‐cell RNA sequencing, spatial transcriptomics

## Abstract

Prostate cancer (PCa) is frequently accompanied by benign prostatic hyperplasia (BPH), highlighting the need to reassess their correlation in tumor risk, malignant progression, and immune status to improve the early diagnosis and treatment of PCa. In this study, single‐cell RNA sequencing and spatial transcriptomics were used to analyze the potential association between normal, BPH, and PCa tissues. Our study revealed a continuous transformation map of luminal epithelial cells from hyperplasia to malignancy in BPH and PCa, accompanied by a persistently suppressive immune microenvironment. In the lesion nodules, T cells showed a high degree of infiltration yet showed activation retardation and functional exhaustion. Macrophages were also significantly infiltrated, exhibiting significant M2 polarization characteristics, and inflammatory signaling pathways related to immune escape were activated. Natural killer (NK) cells and B cells were partially activated, yet the low abundance of NK cells and B cells resulted in functional limitations. Our results revealed the dynamic changes of the immune landscape during the occurrence and progression of PCa. Our classification and prognostic models provide a theoretical basis for immunomodulatory and personalized therapies and provide new tools for the early diagnosis and intervention of PCa.

## Graphical abstract

Leveraging integrated single‐cell and spatial transcriptomics, we delineated the sustained immunosuppressive landscape during prostate disease progression. Based on this, we have developed a precise gene‐based diagnostic classifier and a multimodal prognostic model. Results provide actionable clinical insights for future preclinical validation.

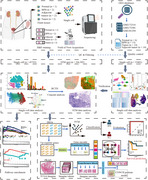

## 1 Introduction

Prostate cancer (PCa) is the second most common cancer among men, representing approximately 14.1% of all male cancers, with over 1.4 million new cases diagnosed annually [1]. Historical autopsies indicate that 83.3% of PCas develop in prostates with concomitant benign prostatic hyperplasia (BPH) [2]. Epidemiological data indicate that the presence of BPH increases the risk of developing PCa [3, 4, 5]. The complex functional and structural heterogeneity between luminal and basal epithelial (LE and BE) cells of the prostate, particularly the multifunctional characteristics of luminal cells following stimulation, has led to a reexamination of the relationship between BPH and PCa development [6]. Prostate‐specific antigen remains a sensitive serum marker for PCa. Nevertheless, due to frequent false positives caused by BPH, its specificity may be affected [7]. The overlap of PCa and BPH symptoms and biomarker characteristics makes it difficult to distinguish between the two diseases. This highlights the need to revisit the association between BPH and PCa.

The immune microenvironment includes cellular, molecular, and structural networks. In cancer, both cellular and noncellular components within the immune microenvironment coordinate together through this network and evolve with tumor progression [8, 9]. This interaction often leads to the formation of “immune deserts” or “cold” tumors.

In PCa, T‐cell deficiency impairs effective antitumor responses [10], and tumor‐associated macrophages (TAMs) significantly contribute to immune escape in PCa. TAMs often exhibit an immunosuppressive phenotype, which promotes tumor growth and weakens the antitumor immune responses [11]. In PCas, TAMs often highly express immunosuppressive factors, including IL‐10 and TGF‐β, which in turn inhibit the activation of T cells and natural killer (NK) cells [9, 12]. This process forms an immunosuppressive environment and mediates tumor resistance to immunotherapy. T cells are also critical in the PCa microenvironment. T cells are often in a suppressed, dysfunctional, or exhausted state, which is mainly due to the upregulation of immune checkpoints, including PD‐1 and CTLA‐4, which can inhibit T cell activation and enhance immune tolerance [13]. Moreover, cytokines present in the PCa microenvironment, including TGF‑β and IL‑2, further compromise the functional activity of T cells [14]. Immune deserts in PCa also limits the effectiveness of immune checkpoint inhibitors (ICIs) such as pembrolizumab (Keytruda or K‐drugs). In the KEYNOTE‐199 clinical trial, the objective response rate to pembrolizumab was only 5% even among PD‐L1‐positive patients with metastatic castration‐resistant PCa (CRPC) [15]. Therefore, immunotherapy is currently recommended primarily for patients with advanced or metastatic PCa. This limited immune response highlights the need to further explore the tumor immune microenvironment, which is of great significance for overcoming immunotherapy resistance and developing new therapeutic strategies.

Single‐cell and spatial transcriptomics (STM) focus on cellular heterogeneity, stemness, tumor microenvironment, and tumor marker analyses. Single‐cell RNA sequencing (scRNA‐seq) is useful for diagnosing and treating PCa. Cheng et al. found that CRPC may result from acquired evolutionary selection during androgen deprivation therapy and also be related to highly adaptive CRPC‐like cells in the early stage of PCa [16, 17]. STM offers insights into the spatial distribution and intratumoral substructure of cancer immunity and has great advantages in elucidating understudied immune functions and discovering new immune markers. Additionally, secreted phosphoprotein 1 positive macrophages and CAFs create a tumor immune “barrier” in nonresponders to immunotherapy, but this barrier is not present in responders to ICI treatment [18, 19]. Combining scRNA‐seq and STM helps reveal the unclear relationship between BPH and PCa and reveal spatiotemporal differences in gene expression at the cellular and tissue levels, and enhances the understanding of the complex mechanisms of prostate diseases, thereby facilitating the development of personalized treatment.

In this study, we used single‐cell and STM to investigate the relationship between BPH and PCa progression, explore changes in the immune microenvironment during disease progression, analyze the mechanisms of immunotherapy resistance in patients with PCa, and establish a prognostic model for PCa, aiming to assist clinicians in the precise treatment of patients with PCa. Based on a comprehensive analysis of six normal (NC), 13 BPH, 14 Adj_PCa, and 14 PCa samples from the gene expression omnibus (GEO) databases (GSE172316, GSE172301, and GSE181294), our results were preliminarily verified.

## 2 Results

### 2.1 The Dynamic and Spatiotemporal Single‐Cell Cross‐Tissue Landscape of Normal Prostate, BPH, and PCa

Specimens from various donors were included in this study, including one normal prostate (NC), two hyperplastic prostates (BPH1 and BPH2), two PCa specimens (PCa1 and PCa2), and their corresponding paraneoplastic specimens (Adj_PCa1 and Adj_PCa2), as shown in Table S1.

Seven samples were obtained for scRNA‐seq and STM to comprehensively map the local and systemic dynamics. Appropriate fields of view were selected for spatial transcriptome sequencing (Figure S1A). The cellular and genetic information provided by scRNA‐seq and STM are shown in Table S2.

### 2.2 Harmonized Cellular and Tissue Processes Within the Tumor Ecosystem

**FIGURE 1:**
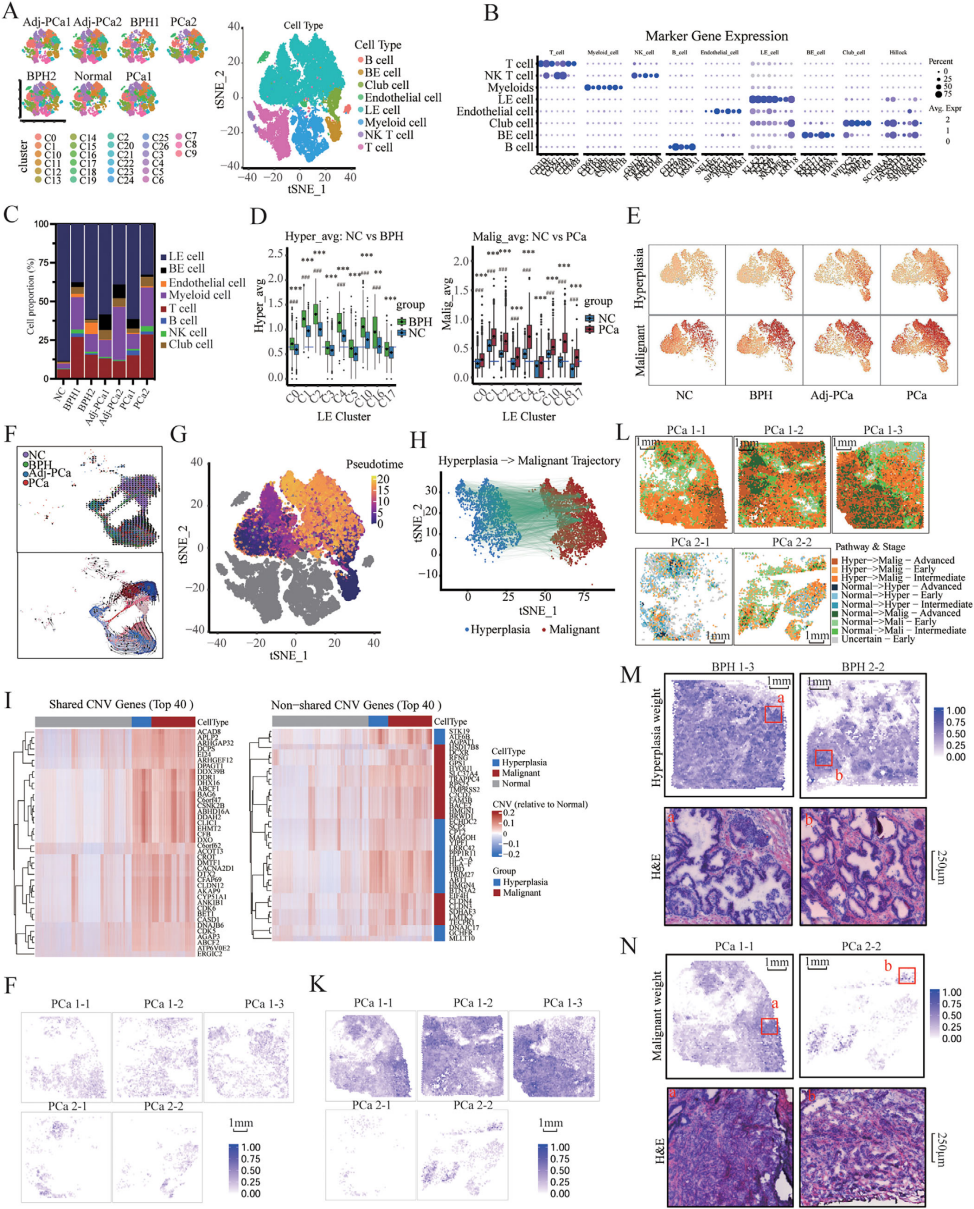
Potential malignant transformation within prostatic epithelial hyperplastic nodules. (A) Integrated analysis of scRNA‐seq samples visualized using a common tSNE embedding for cell annotation (left) and sample fractions (right). (B) Dot plot showing the expression of key marker genes (Table S3) across major cell types. Darker colors indicate higher expression levels, and larger dots indicate a higher proportion of cells expressing the gene. (C) Stacked bar plot showing the cell type composition of each sample based on cell number fractions. (D) Box plots showing the average expression of hyperplasia (left) and malignant (right) signature genes in each epithelial cluster. Asterisks (*) indicate clusters that differ significantly from the corresponding NC cluster, while hash symbols (#) indicate clusters that differ significantly from the overall NC population. *p < 0.05, **p < 0.01, ***p < 0.001; #*p* < 0.05, ##*p* < 0.01, ###*p* < 0.001. (E) t‐SNE heatmap showing the average expression of the genes from panel (D) across epithelial cells. (F) RNA velocity‐inferred developmental trajectories of epithelial cells. Trajectories are shown by sample (top) and by cluster (bottom). Arrows indicate the inferred direction of development, and colors correspond to the target cells. (G) Pseudotime analysis illustrating the simulated developmental trajectory of epithelial cells. (H) Pseudotime analysis identifying epithelial cells with potential progression from hyperplasia to malignancy. Blue indicates hyperplasia cells, red indicates malignant cells, and green arrows indicate the inferred direction of transformation. (I) Heatmap of genes within shared and unique copy number variations (CNVs) in hyperplasia and malignant epithelial cells relative to normal cells. Each column represents a single cell; red indicates amplification, and blue indicates deletion. (J and K) Spatial transcriptomic map of hyperplasia (J) or malignant (K) weights in epithelial cells of PCa tissues. Each point represents a detected spot. (L) Spatially resolved transformation pathways in prostate cancer tissues. The heatmap displays the dominant transformation pathways and progression stages across PCa samples. Colors indicate three distinct transformation trajectories: blue (normal to hyperplasia), orange (hyperplasia to malignant), and green (normal to malignant). Color intensity represents transformation progression, with light shades indicating early stages, medium shades intermediate stages, and dark shades advanced stages. (M and N) Spatial transcriptomic atlas of hyperplasia (M) and malignant (N) cells in different prostate tissues. Spatial transcriptomic deconvolution was performed using RCTD based on the top 100 signature genes of normal, hyperplasia, and malignant cells. In panel (M), the upper image depicts the hyperplasia weights of epithelial cells in hyperplastic tissue, and the lower image shows H&E staining of the region highlighted by the red box. In panel (N), the upper image depicts the malignant weights of epithelial cells in tumor tissue, and the lower image shows H&E staining of the region highlighted by the red box. Each dot represents a detected spot.

Overall, 45,763 high‐quality single cells were obtained after quality control. Common tSNE embedding analysis identified major cell types, including immune cells (T cells, B cells, NK cells, and myeloid cells), endothelial cells, and epithelial cells. Epithelial cells comprised LE cells (identified using typical epithelial cell markers), BE cells, and club cells (Figure 1A,B and Table S3).

Immune cells showed significant recruitment in BPH and PCa tissues compared with the normal prostate tissues. Relative to PCa tissues, adjacent PCa tissues showed suboptimal recruitment of T cells, B cells, and NK cells. Additionally, B cells and NK cells maintained a low abundance in all tissues (Figure 1C).

Common uniform manifold approximation and projection (UMAP) embedding analysis of 266,505 cells from GEO datasets (Table S4, see the methodology section for details) revealed major cell types, including immune cells (T cells, B cells, NK cells, and myeloid cells), endothelial cells, fibroblasts, mast cells, and epithelial cells after removing low quality cells. Epithelial cells were identified as LE cells, BE cells, and club/hillock cells (Figure S1B,C and Table S5). Immune cell subsets mainly consisted of myeloid cells, T cells, B cells, and NK cells, and the distribution pattern of these cells in different tissues was consistent with the above results. Notably, there was no significant difference between PCa tissue and Adj_PCa tissue (Figure S1D).

### 2.3 Altered Interactions Between Prostate Epithelial Cell Evolution and the Cellular Immune Microenvironment

Prostate lesions are accompanied by the activation of specific genes. Marker genes for BPH [20, 21] and PCa [22, 23] were significantly overexpressed in clusters C1, C2, C4, C10, C13, and C16 (Figure 1D,E and Table S6). Examination of RNA velocity (the change in messenger RNA (mRNA) molecular abundance with time in cells) and pseudo‐time analysis was used to reconstruct the evolutionary trajectory of epithelial cells during prostate lesion progression. C1, C2, C4, C10, C13, and C16 were mainly located at the end of the trajectory, representing the terminal differentiation state. Notably, benign lesions may experience the same pathway as malignant lesions (Figure 1F,G). Moreover, a number of hyperplastic cells exhibit developmental trajectories that point toward malignant cells (Figure 1H). This observation suggests a risk of malignant transformation of hyperplastic epithelial cells, or that some malignant cells may have originated from a hyperplastic‐like precursor state. We performed integrated copy number variation (iCNV) analysis using the average gene expression across each chromosomal region (Figure S2A). Multiple CNVs were identified, corroborating a previous report [24] and further confirmed the malignant identity of cells within clusters C1, C2, C4, C10, and C16. Notably, hyperplastic cells also exhibited CNVs highly similar to those of malignant cells, indicating the presence of widely shared mutant segments in hyperplastic and malignant cells [25] (Figure 1I and Table S7). These results suggest a potentially shared progression pathway, leading us to hypothesize that certain malignant cells may have undergone a hyperplasia‐like transition. For these clusters, cells were annotated as normal, hyperplasia, Adj_hyperplasia, or malignant. The phylogenetic tree constructed by hierarchical clustering of CNV patterns inferred from scRNA‐seq data showed a topology that supports our hypothesis (Figure S2B).

We queried genes that were highly expressed (*p* < 0.05, Log2 FC > 1.5) in cells with distinct lesion characteristics. Results showed that OLFM4, SCGB3A1, MT1G, MT1M, MT1X, MT1F, PI15, and MT2A were highly expressed in hyperplastic cells, whereas SPNK1, FABP5, TGM4, PRR4, SERPINA3, NCAPD3, CEBPD, TGM2, ALOX15B, TFF3, PAK1IP1, IGFBP5, MMP7, MGP, TM4SF1, C3, IFITM2, TRPM4, STEAP4, and FKBP5 were highly expressed in cells with malignant features (Figure S2C).

### 2.4 STM Reveals Alterations in the Immune Microenvironment During the Evolution of Epithelial Cells

In the regions corresponding to single‐cell samples, 12 fields of view (36 mm^2^ each) were selected for 10× STM sequencing (Figure S2D). Overall, 53,401 high‐quality STM spots were identified after quality control. Signature genes were calculated for the major cell types observed in the single‐cell data, including T cells, B cells, NK cells, myeloid cells, endothelial cells, LE cells, BE cells, and club cells. The top 100 genes for each cell type (Table S8) were used for STM data decomposition analysis based on robust cell type decomposition (RCTD) (Figure S2E). Based on RCTD deconvolution, the spatial distribution of malignant weights of epithelial cells in PCa tissues was visualized, revealing regional differences in malignancy levels (Figure 1J,K). Subsequently, potential cellular transition pathways, including normal‐to‐hyperplasia, hyperplasia‐to‐malignant, and normal‐to‐malignant pathways, were identified by integrating the weighted transformation index of LE cells with single‐cell trajectory predictions, and results revealed the spatial diversity of malignant evolution in PCa (Figure 1L). Additionally, we calculated the top 100 signature genes (Table S8) for different cell states within the LE cell population and applied these genes in RCTD analysis, successfully mapping hyperplastic and malignant epithelial nodules on spatial slides (Figure 1M,N).

### 2.5 Macrophages and Monocytes with the Highest Infiltrating Abundance in the Tumor Immune Microenvironment

**FIGURE 2:**
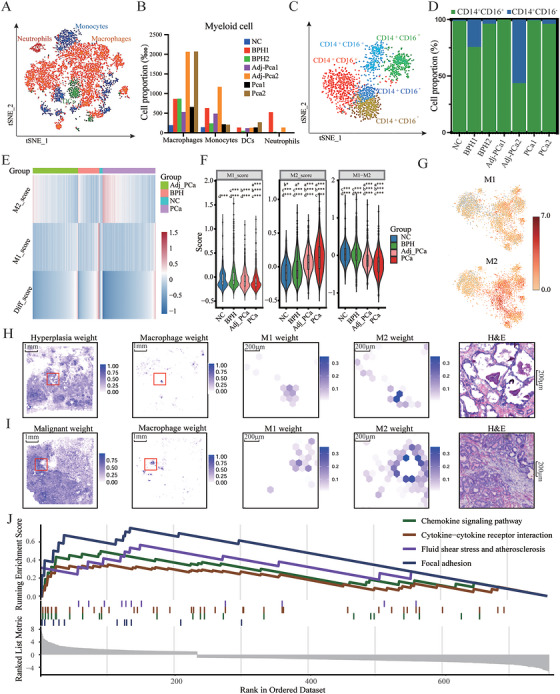
Inflammatory infiltration may promote tissue malignancy. (A) Annotation of myeloid cell subpopulations. (B) Proportional distribution of myeloid cell subpopulations across samples. (C) Annotation of monocyte subpopulations. (D) Activation states of monocytes in different tissue types. (E and F) Module scores for M1 and M2 polarization, and their difference (M1−M2), calculated for each macrophage based on signature genes. a, vs. NC; b, vs. BPH; c, vs. Adj_PCa; d, vs. PCa. *p < 0.05, **p < 0.01, ***p < 0.001. (G) t‐SNE plot showing the log‐transformed average expression of M1 and M2 macrophage polarization markers in macrophages. (H and I) Spatial characterization maps of epithelial cells (H) and macrophage (I) polarization in different prostate tissues. From left to right: weight of epithelial hyperplasia or malignancy, spatial distribution weight of macrophages, M1 and M2 polarization score weights in the red‐boxed area, and H&E staining. Each dot represents a detected spot. (J) Upregulated genes in PCa are significantly enriched in inflammation‐related pathways.

Myeloid cells were further clustered and identified as macrophages (*CD68, CD163, CD14, MRC1, CD86, CD11B*), monocytes (*CD14, CD33, ACPP, CD68*), neutrophils (*PI3, FCGR3B, TNFAIP6, CXCL8*), and dendritic cells (*DCs; FCER1A, HLA*‐*DQB2, CD1C, FLT3*) (Figure 2A). These cells were more widely distributed in BPH and PCa tissues than in NC tissues and were dominated by monocytes and macrophages. In PCa tissues, macrophages were scattered in the paracancerous and tumor areas, while the infiltration of monocytes into the tumor center was impeded (Figure 2B). Monocytes were further clustered and identified as classical (CD14+CD16−) and nonclassical (CD14+CD16+) subsets based on the expression of CD14 and CD16 [26] (Figure 2C). Classical monocytes, recognized for their phagocytic role, were elevated only in BPH1 and Adj_PCa2 cells (Figure 2D).

In the GEO datasets, myeloid cells are classified into monocyte, macrophage, DC1, and DC2 subtypes, with macrophages as the predominant population. Unlike our data, there were no differences in distribution between tumor and Adj_PCa tissues. In normal and pathological environments, human monocytes are recruited into tissues and differentiated into macrophages or dendritic cells [26]. Macrophages were relatively more abundant than in NC samples; however, their proportion remained low, indicating that the differentiation of monocytes into macrophages was partially restrained upon infiltration into PCa tissue relative to normal tissue. Notably, this phenomenon was not observed in patients with BPH (Figure S3A,B).

Macrophages polarized into M1 (classically activated macrophages) and M2 (alternatively activated macrophages) subtypes [27]. We used a clustering technique [28] to evaluate the average expression level of marker genes and determined the polarization state of macrophages (Table S9). Macrophages showed a pronounced M2 polarization tendency in both BPH and PCa tissues, which may intensify as the disease progresses (Figure 2E–G). This M2 polarization tendency was also observed in GEO datasets (Figure S3C,D). Subsequent RCTD analysis based on macrophage signature genes also revealed macrophage accumulation within the lesioned nodules, and tended toward M2 polarization (Figure 2H,I).

Furthermore, gene set enrichment analysis (GSEA) on genes highly expressed in PCa showed that the chemokine signaling pathway, cytokine–cytokine receptor interaction, fluid shear stress, atherosclerosis, and focal adhesion pathways were significantly enriched (Figure 2J). These pathways are closely related to inflammatory activation, suggesting that persistent inflammation is the key mechanism underlying BPH progression to PCa.

### 2.6 Dysfunction is a Key Feature of T Cells

**FIGURE 3:**
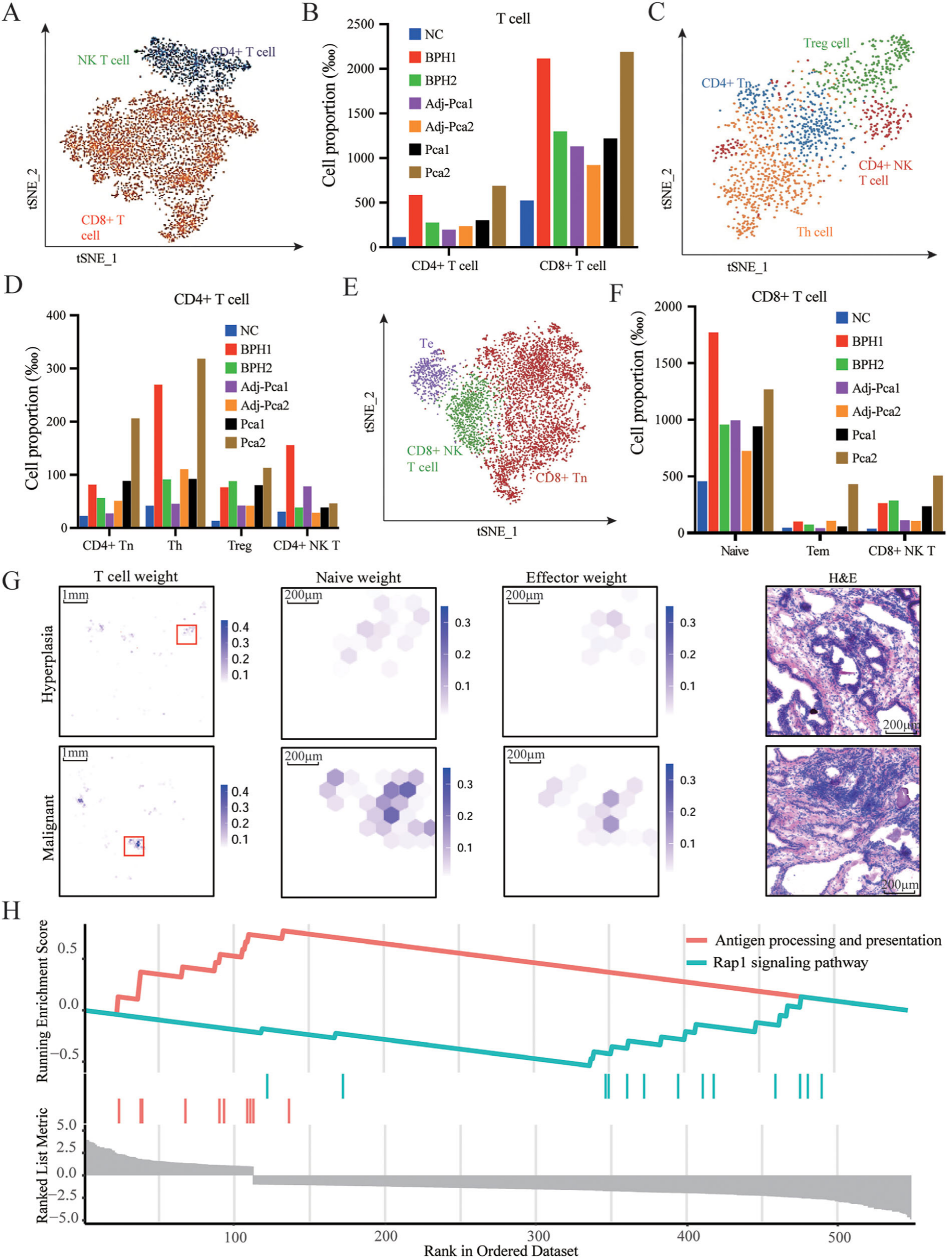
The activation of inhibitory T cells and the difficulty of antigen presentation may be the cause of the difficulty of PCa immunotherapy. (A) Cell annotation of T cell subpopulations. (B) Infiltration of CD4+ T cells and CD8+ T cells in prostate tissue samples. (C) Cell annotation of CD4+ T cell subpopulations. (D) Infiltration of CD4+ T cell subsets in different tissue samples. (E) Cell annotation of CD8+ T cell subpopulations. (F) Infiltration of CD8+ T cell subsets in different tissue samples. (G) Spatial transcriptomic atlas of T cells and their identities across BPH or PCa tissues. From left to right: T cell weight derived from spatial transcriptomic deconvolution using RCTD (based on the top 100 T‐cell signature genes), module scores of T cell naive signature genes, module scores of T cell effector signature genes, and H&E staining of the red‐boxed region. Each dot represents a detected spot. (H) Upregulated genes in PCa are significantly enriched in antigen presentation‐related pathways.

The T cells were further clustered and identified as CD4+ T cells (*CD4+*), CD8+ T cells (*CD8A+*, *CD8B+*), and NKT cells (*KRT86+*, *AREG+*, *CD4*−, *CD8A*−, *CD8B*−) (Figure 3A). CD4+ and CD8+ T cells recruitment increased in patients with BPH and PCa, with higher recruitment observed in the tumor areas of patients with PCa (Figure 3B). T cell differentiation maintained a consistent proportion of CD4+ and CD8+ T cells, with a tendency toward CD8+ differentiation in all samples. However, patients with tumors in the GEO datasets exhibited reduced CD8+ differentiation, and no differences were observed between the tumor and adjacent nontumor regions (Figure S4A,B).

CD4+ T cells were clustered and identified as CD4+ naïve T cells (CD4+ Tn) (*SELL, CCR7, FAS−, LEF1*), T helper (Th) cells (*CCR6, CAPG, TNF*), regulatory T cells (Treg) (*IL2RA, FOXP3, TNFSF18, CTLA‐4*), and CD4+ NK‐like T cells (CD4+ NKT) (*IKZF3, MACF1, SYNE2, LNPEP*, and *NEAT1*) (Figure 3C). Composition analysis revealed that the CD4+ T cells accumulating in the lesioned tissues were primarily nonactivated Tn cells, along with Th and Treg cells involved in immune regulation, with a more significant accumulation observed in the tumor lesions (Figure 3D). This accumulation severely limits the function of CD4+ T cell subsets in BPH and PCa, potentially inhibiting the function of other immune cells and promoting tumorigenesis and progression.

CD8+ T cells were clustered and identified as naïve CD8+ T cells (CD8+ Tn) (*CD55, IL7R, CD27*), effector CD8+ memory (Tem) cells (*ADGRG1, FGFBP2*), and CD8+ NK‐like T cells (CD8+ NKT) (*GZMK, GZMM, DUSP2*) (Figure 3E). In all samples, most CD8+ T cells were in a naïve state, indicading that CD8+ T cell activation may be inhibited (Figure 3F).

Functional and activation inference of T cell subpopulations in GEO datasets corroborated our findings. CD4+ T cells showed negligible expression of cytotoxicity‐related genes (Figure S4C); the high expression of IL7R and CD69 indicated that these cells were predominantly in an early activation stage without clear differentiation into effector or cytotoxic cells. This indicates that CD4+ T cells are maintained in a dysfunctional naive/early activation state or progress to a terminal effector stage with impaired function (Figure S4D,E). For CD8+ T cells, subsets expressed effector molecules but more prominently expressed IL7R, PTPRC, and CD69 (Figure S4F). The rightward path in the developmental trajectory represents the silent route, whereas the leftward path represents activation. Only a small fraction of of cells retained memory markers and showed partial cytotoxic potential (Figure S4G,H). These patterns were particularly pronounced in patients with tumors (Figure S4I,J and Table S10).

Deconvolution analysis of T cells, combined with naïve gene module scoring, revealed that T cells infiltrating hyperplastic and malignant epithelial nodules showed pronounced naïve characteristics (Figure 3G).

Similarly, a GSEA enrichment analysis of the differentially expressed genes (DEGs) was conducted. The upregulated genes in PCa did not enrich any significant pathways; however, the downregulated genes in PCa were enriched in pathways associated with antigen processing and presentation, including the antigen processing and presentation pathway and the Rap1 signaling pathway (Figure 3H). In general, T cells were inactivated or dysfunctional as a whole, which helps to maintain the immune desert state of PCa and may be associated with antigen presentation defects.

### 2.7 NK Cell Expression is Elevated in Response to Chronic Inflammatory Stimulation and Prolonged Tumor Antigen Exposure

**FIGURE 4:**
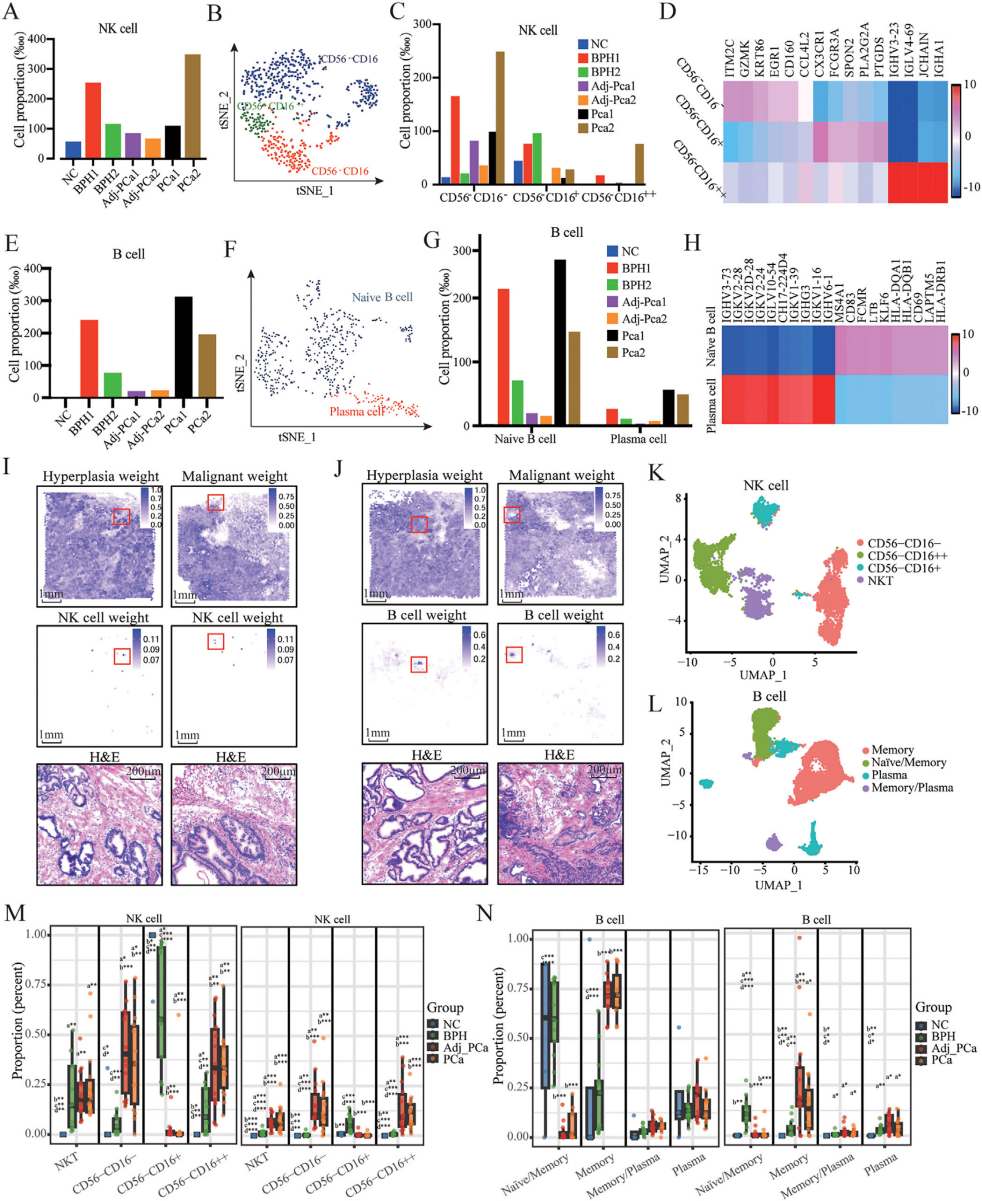
NK cells and B cells remain at low abundance across prostate lesions. (A) Distribution of NK cells across individual samples based on cell number fractions. (B) Annotation of NK cell subpopulations. (C) Distribution of NK cell subpopulations across different tissues based on cell number fractions. (D) Top five differentially expressed genes in NK cells with varying activation states. (E) Distribution of B cells across individual samples based on cell number fractions. (F) Annotation of B cell subpopulations. (G) Distribution of B cell subpopulations across different tissues based on cell number fractions. (H) Top five differentially expressed genes between naive B cells and plasma cells. (I and J) Spatial transcriptomic atlas of NK cells, B cells, and epithelial cells in different prostate tissues. Spatial transcriptomic deconvolution was performed using RCTD based on the top 100 signature genes. The top panel shows hyperplasia or malignant weights of epithelial cells in the tissue, the middle panel displays the spatial distribution and weights of NK (I) or B (J) cells, and the bottom panel shows H&E staining of the region highlighted by the red box. Each dot represents a detected spot. (K) Annotation of NK cell subpopulations in the GEO dataset. (L) Annotation of B cell subpopulations in the GEO dataset. (M) Proportional composition of NK cells at different activation stages within the NK cell compartment (left) and across sample groups (right). a, vs. NC; b, vs. BPH; c, vs. Adj_PCa; d, vs. PCa. *p < 0.05, **p < 0.01, ***p < 0.001. (N) Proportional composition of B cells at different activation stages within the B cell compartment (left) and across sample groups (right). a, vs. NC; b, vs. BPH; c, vs. Adj_PCa; d, vs. PCa. *p < 0.05, **p < 0.01, ***p < 0.001.

NK cells were further clustered to identify *CD56*−*CD16*−*, CD56*−*CD16+*, and *CD56*−*CD16++* cells based on the expression levels of *CD16* and *CD56* [29] (Figure 4A,B). NK cell maturation is characterized by decreased *CD56* and increased *CD16* expression. Overall, NK cells infiltrated in the lesioned tissues with low abundance and were mainly in a relatively low activation state (Figure 4C). Additionally, the top five genes specifically expressed at different stages of NK cell maturation, including *IGHV3‐23, LGLV4‐69, IGHA1*, and *JCHAIN*, which are involved in immune responses, were highly expressed only in mature NK cells and were rarely observed in lesioned tissues (Figure 4C,D). Prolonged chronic inflammation and persistent tumor ligand stimulation lead to *CD56* modification in classical NK cells, reduce their antibody‐dependent cytotoxicity (ADCC), and ultimately weaken the direct killing ability of NK cells. In addition, their low abundance is also a key limiting factor.

### 2.8 The Intricate Dual Role of B Cells in Regulating Epithelial Proliferation and Tumorigenesis

B cells were categorized into naive B cells (*MS4A1+, IGHG1−*) and plasma cells (*MZB1+, IGHG1+, JCHAIN+*). Compared with normal tissues, B cells were more enriched in BPH and PCa tissues, especially in the tumor center, but the abundance of B cells was still very low compared with macrophages and T cells. In addition, most B cells remained inactive across all tissues (Figure 4E,F). Approximately 10% of B cells were activated in BPH tissues, with a higher proportion observed in the adjacent and tumor tissues (Figure 4G). Gene expression analysis revealed that activating naïve B cells was primarily regulated by the increased expression of the V region of the variable domain of immunoglobulin light and heavy chains, which are involved in antigen recognition, along with the decreased expression of *MS4A1*, *KLF6*, and *FCMR* (Figure S3H). These genes indicate a function in the ADCC of activated B cells, yet their low abundance is a key limitation.

The STM deconvolution of NK and B cells (Table S8) revealed their infiltration into hyperplastic and malignant epithelial nodules. Unlike NK cells, which consistently showed a low abundance in lesioned tissues, B cells showed relatively higher infiltration (Figure 4I,J). Consistent with these observations, analyses of the GEO datasets showed similar distribution patterns of NK and B cells within diseased tissues, with no significant changes in plasma cell proportions (Figure 4K,L). In BPH tissues, most B cells remained in a naïve state and had a tendency to differentiate into memory cells. Conversely, B cells in PCa and adjacent tissues predominantly underwent memory‐to‐plasma differentiation, indicating impaired activation or effector function during disease progression (Figure 4M,N). These findings suggest that B cells and NK cells may be dysfunctional during the pathological evolution of prostate lesions.

### 2.9 Immunofluorescence of Immune Cell Infiltration in Prostate Tissue

**FIGURE 5:**
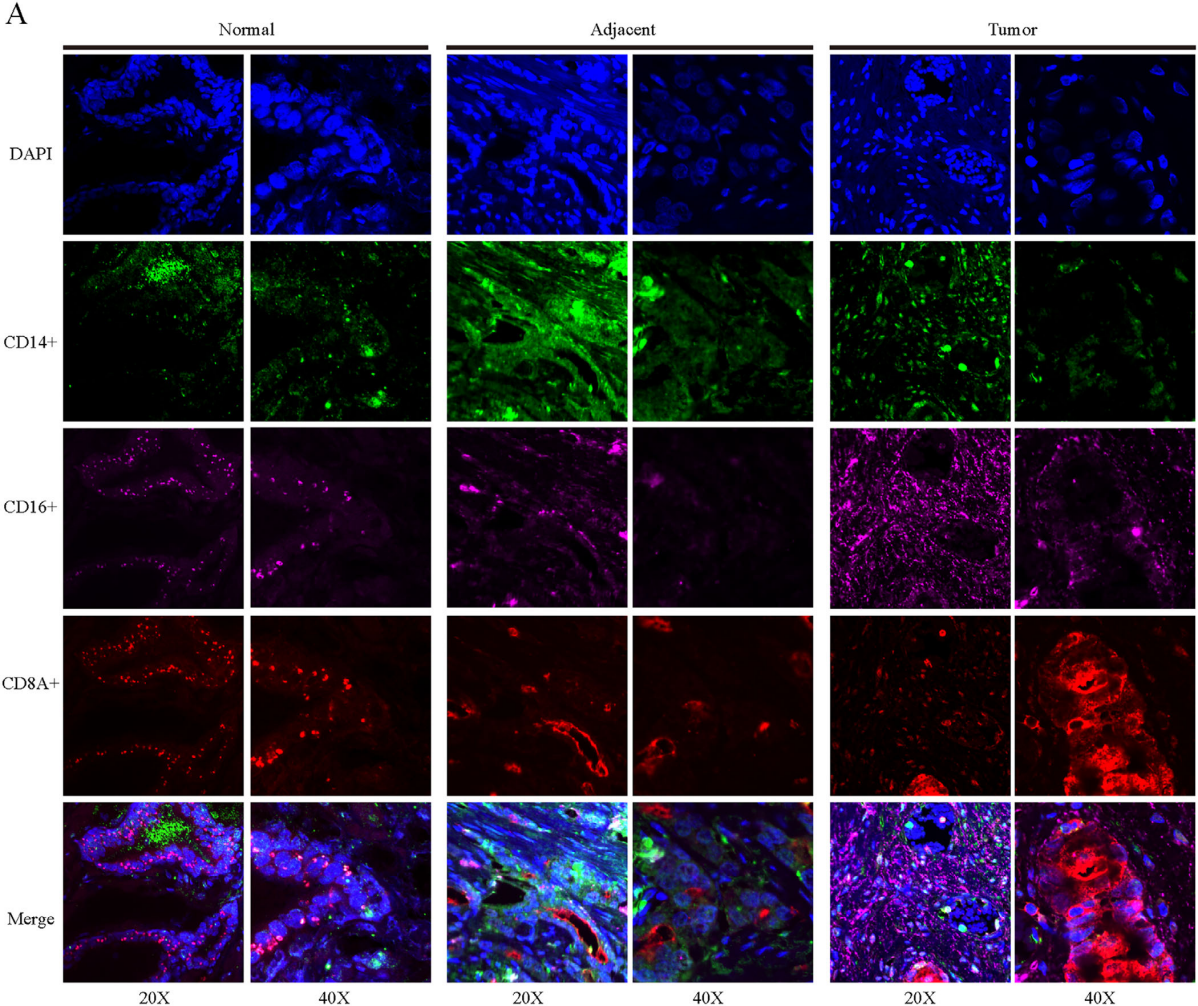
Multiplex immunofluorescence analysis identifying the expression patterns of CD14, CD16, and CD8A in hyperplastic nodules, adjacent hyperplastic regions, and malignant nodules.

To verify the distribution and function of immune cells in normal and PCa tissues, we labeled the expression of CD8, CD14, and CD16 by immunofluorescence. CD8 immunofluorescent staining reproduced the STM distribution profile of CD8+ T cells. Compared with normal tissues, obvious infiltration of CD8+ T cells was observed in malignant nodules, which was more obvious than that in adjacent hyperplastic nodules. Combining the previous functional analyses, the presence of abundant inert CD8+ T cells suggested the establishment of an immunosuppressive microenvironment, which may enhance the resistance to immunotherapy and promote the progression of PCa. CD14 and CD16 were used to identify myeloid cells (given the extremely low abundance of NK cells, the contribution of CD16 as an NK cell marker is negligible). The positive immunofluorescence signals of CD14 and CD16 were enriched in the perilesional regions of adjacent hyperplastic nodules and showed prominent accumulation within malignant nodules. Combined with the above cell typing and functional analysis, these cells were mainly M2‐type polarized macrophages. The results further supported the characteristics of persistent immunosuppressive microenvironment revealed by STM. Moreover, the spatial colocalization of CD14+ and CD8+ cells in PCa tissues suggests that there is a potential interaction between them, which jointly mediates the occurrence and development of prostate lesions (Figure 5).

### 2.10 Regulatory Interactions Between Epithelial Cells and Immune Cells

**FIGURE 6:**
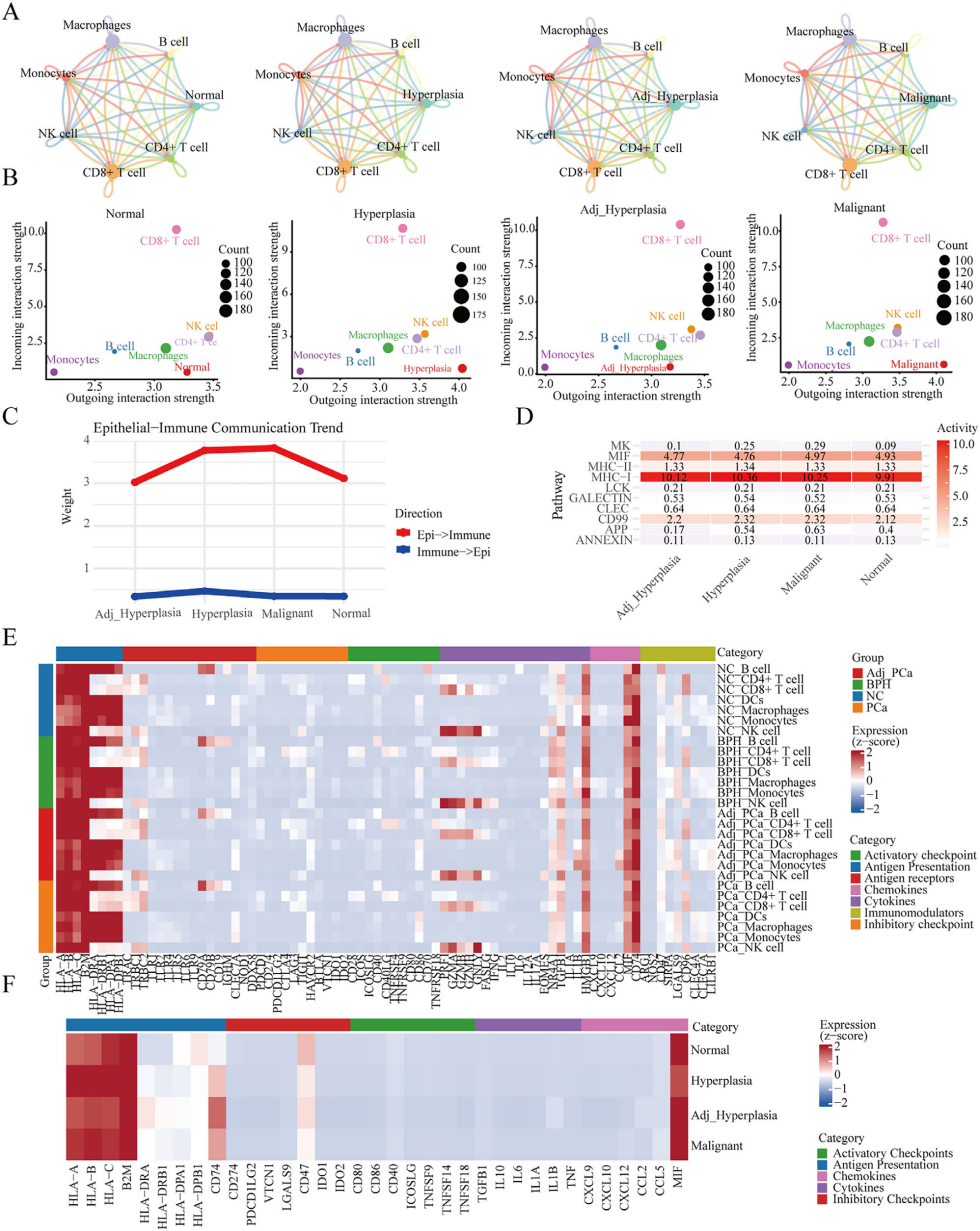
Signal network analysis of interactions between epithelial cells and the immune system. (A) Intercellular signaling interactions between normal, hyperplasia, adjacent hyperplasia, and malignant epithelial cells and major immune cell types. (B) Input and output signaling weights between normal, hyperplasia, adjacent hyperplasia, and malignant epithelial cells and major immune cell types. (C and D) Signaling exchange patterns and major signaling pathways (top 10) between epithelial cells in different states and immune cells. (E and F) Heatmaps showing the expression of immunoregulatory genes in immune cells (E) and epithelial cells (F). Expression values were log‐normalized and *Z*‐score standardized.

The interactions between different cell types within the immune microenvironment jointly construct a complex signaling network. We simulated intercellular communication networks between epithelial cells in different pathological states and major immune cell types (Figure 6A). Epithelial cells consistently showed low signal reception but high signal output within cellular communication networks, primarily through the major histocompatibility complex I (MHC‐I) pathway, followed by MIF and CD99 signals (Figure 6B–D).

MHC molecules are essential in antigen presentation, particularly in evading immune surveillance in cancers. LE cells primarily utilize MHC‐I (*HLA‐A, HLA‐B, HLA‐C*, and *HLA‐E*), which was upregulated in hyperplastic cells. Antigen‐presenting cells, including macrophages, DCs, and monocytes, express both MHC‐I and MHC‐II at high levels. However, molecules responsible for recognizing antigenic messages (*TRAC*, *TRBC*, C‐type lectin receptors, and Toll‐like receptors) were consistently expressed at low levels in immune cells. Moreover, once immune cells successfully recognize antigenic information, they will be activated or proliferated under the combination of antigen and costimulation. This process is also be regulated by immune checkpoints (including agonistic and inhibitory immune checkpoints) and costimulatory factors. However, we did not observe significant changes in the expression of immune checkpoints, which may be a key factor in patients rarely benefiting from immunotherapy. Notably, *HMGB1* was significantly expressed in most cells, and its release may trigger a cytokine storm that subsequently leads to immune cell death and immunosuppression. Another notable cytokine is the expression of TGFB1, which enhances tumor progression. Furthermore, factors that directly kill target cells, including interleukins and perforins, were only expressed in NK cells and lowly expressed in CD8+ T cells, with no regular changes observed. In contrast, factors that inhibit immune cell function were significantly expressed, mainly *MIF*, *CD74*, and *TGFB1* (Figure 6E).

High MHC‐I expression was observed in LE cells. Another notable feature is the persistently high expression of MIF, which contributes to the anti‐inflammatory, immune‐evasive, and immune‐tolerant phenotypes of innate and adaptive immune cell types. The concurrent high expression of MIF and increased expression of CD74 represent a characteristic molecular signature of proliferating and malignant cells. Additionally, CD47, which releases the “do not eat me” signal, was relatively reduced (Figure 6F).

Overall, the dominant immune cell subsets maintained the suppressive immune microenvironment in NC, BPH, and PCa.

### 2.11 Validation of DEGs and Support Vector Machine‐Based Classification of BPH and PCa

**FIGURE 7:**
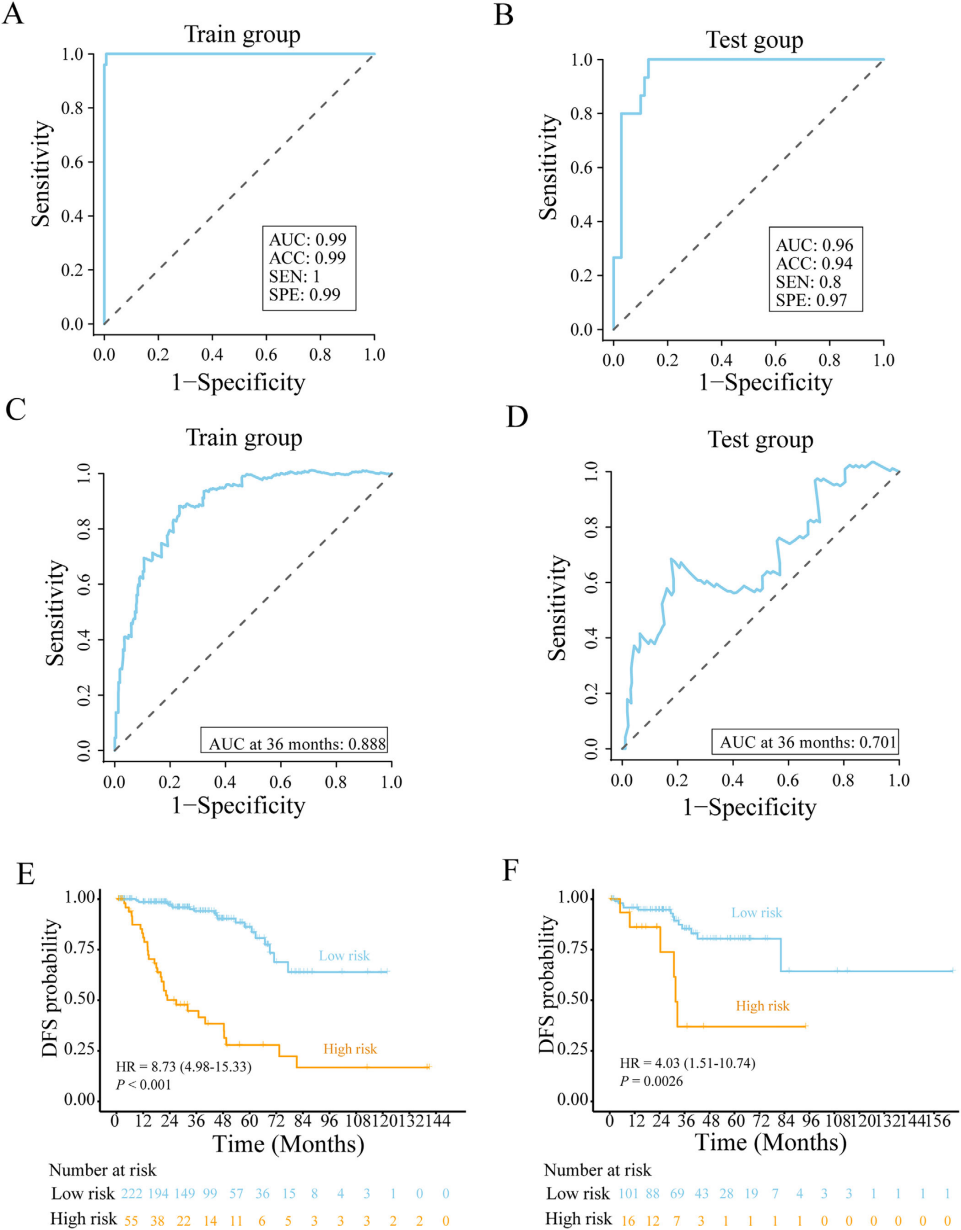
Construction of classification and prognostic models. (A) Area under the receiver operating characteristic curve (AUC) of the classification model training group. (B) AUC of the classification model test group. (C) AUC of the prognostic model training group. (D) AUC of the prognostic model test group. (E) Survival curve of the prognostic model training group. (F) Survival curve of the prognostic model test group.

We validated the DEGs obtained from the single‐cell analyses using public databases. Overall, 1091 DEGs with |log2FC| ≥ 1 were identified across three GEO datasets, containing 281 patients. All three datasets were batch corrected. A binary classification model distinguishing BPH from PCa was constructed using the support vector machine (SVM). The area under the receiver operating characteristic (ROC) curve (AUC) and accuracy (ACC) indicated that the model had good predictive performance. Specifically, the AUC and ACC were 0.99 and 0.99 in the training set, and 0.96 and 0.94 in the testing set, respectively (Figure 7A,B).

We further constructed a multimodal model based on gene‐pathology data. Pathological slides from the TCGA were used to extract features using the Contrastive learning from Captions for Histopathology (CONCH) algorithm. DEGs from 322 T and myeloid cells were identified. A prognostic model was constructed using a multimodal fusion decoder. The AUC at 36 months indicated that the model had good predictive performance (AUC was 0.888 in the training group and 0.701 in the testing group) (Figure 7C,D). Further survival analysis revealed that patients in the training and testing groups could be classified into high‐ and low‐risk subgroups, with significant prognostic differences between the groups (Figure 7E,F).

## 3 Discussion

This study offers a comprehensive spatiotemporal atlas of NC tissue, BPH, and PCa at single‐cell resolution by integrating scRNA‐seq and STM. We revealed new insights into immune landscape remodeling and epithelial cell evolution during the progression of potential prostate lesions by analyzing the cellular composition, gene expression patterns, and interactions within the tumor microenvironment. Notably, the continuous transformation was limited to the transformation of hyperplastic and malignant nodules. In addition, BPH influences the development of PCa. This study further identified significant differences in the expression of inflammatory cytokines across tissues, which affect leukocyte–cell adhesion pathways and phagocyte‐mediated immunity. The immune microenvironment of PCa, different from the conventional “cold tumor” phenotype, showed higher infiltration of myeloid cells and T cells, while with exhibiting characteristics of low activation or functional inhibition. Analyses and prognostic models constructed from multiple public datasets further substantiate the conclusions of this study.

The unique immune microenvironment of the prostate makes PCa insensitive to immunotherapy, resulting in a lack of effective clinical treatment options [30, 31]. Therefore, targeted remodeling of the immune microenvironment is essential for effective intervention of prostate lesions. This strategy regulates the recruitment, activation, and cell–cell communication of immune cells and modulates the levels of cytokines [32], immune checkpoints, [33] and antigen‐presenting molecules [34]. Our results showed significant differences in the distribution of epithelial, myeloid, and immune cells among patients with NC, BPH, and PCa. The scRNA‐seq results indicated that T cells accumulated in the tumor center were mainly exhausted or suppressed. The abundance of inactive T cells creates a suppressive immune microenvironment, leading to disease establishment with almost no efficacy in PCa immunotherapy. MHC molecules act as a bridge between cancer cells and immune cells, promoting the recognition and targeting of cancer cells [35]. Our findings indicated that LE cells primarily present antigenic information to immune cells via MHC‐I, while immune cells recognize these antigens via lowly expressed T cell receptors. Immune cells then exchange antigenic information via MHC‐I and MHC‐II. Therefore, regulating the immune microenvironment of prostate tissues, especially activating T cells and enhancing the function of immune cells through cytokines, is expected to play an intervention role in the occurrence and development of PCa.

Myeloid cells are essential in the initiation and progression of PCa. Chronic inflammation promotes PCa through multiple pathways. Myeloid‐derived suppressor cells (MDSCs) represent an important subset of myeloid cells in the PCa microenvironment [36]. MDSCs weaken the body's immune surveillance by inhibiting the functions of effector T and NK cells, which are key antitumor immune cells. Chronic inflammation enhances the recruitment and activation of myeloid cells, including MDSCs and TAMs. Myeloid cells secrete a variety of inflammatory cytokines and growth factors, which further exacerbate inflammation and tumor progression [37]. We observed that most myeloid cells in the prostate tissue exhibited immunosuppressive properties. Macrophages were highly recruited to both cancerous and adjacent tissues, whereas monocytes only infiltrated into paracancerous areas and failed to migrate into the tumor core. Gene oncology pathway enrichment of DEGs also indicated that the inflammatory pathways remained persistently activated as the lesions progressed. BPH appears to have already established a highly immunosuppressive microenvironment. Although BPH is often accompanied by prostatic inflammation, the current clinical guidelines do not recommend the combination of anti‐inflammatory regimens in the conventional treatment of BPH. Based on our findings, active anti‐inflammatory treatments during BPH progression, such as the use of indomethacin, may help reduce the incidence of PCa. Furthermore, upregulation of *CD74* and high expression of MIF constituted the key molecular axis to promote the formation of immunosuppressive microenvironment in PCa. This axis not only drives the autonomous malignant behavior of tumor cells, but also actively shapes the immunosuppressive microenvironment conducive to tumor growth [38, 39]. Therefore, therapeutic strategies aimed at enhancing phagocytosis while simultaneously targeting *CD74* and MIF might be more effective, and this method may also be applicable to BPH patients.

The prognosis of immunotherapy depends on the infiltration of immune cells and the expression of immune checkpoints. Tumors with low immune cell infiltration and low immune checkpoints expression are generally known as “cold tumors” [40]. However, our findings revealed a more complex scenario in PCa. Substantial infiltration of innate and adaptive immune cells was observed within the malignant nodules. Paradoxically, most of the widely studied inhibitory immune checkpoints and their corresponding receptors [41, 42] were either not expressed or maintained at low levels in most lesion cells or infiltrating immune cells. Consequently, related inhibitors and antibodies may prove to be ineffective, prompting us to consider the induction of immune checkpoint expression and activation. It is worth noting that the results cannot rule out the low detection rate of immune checkpoints or the limited sensitivity of sequencing technology. Antigen‐presenting cells activated by sipuleucel‐T carry the prostatic acid phosphatase antigen and present as an antigen peptide–MHC complex to the patient's T cells, thereby activating the T cells to mediate tumor cell killing [10]. The therapeutic application of recombinant human cytokines represents a potential strategy for cancer immunotherapy [43, 44, 45]. Based on our analysis, remodeling the immune microenvironment by enhancing the antigen presentation capacity and secreting cell activation factors may be a potential therapeutic approach for PCa. Combined with the results that activated inflammatory pathways promote PCa progression, combining PCa immunotherapy with inflammatory cytokine‐based therapies, using inflammatory cytokines and the resulting ADCC and complement‐dependent cellular cytotoxicity (CDCC) effects to transform the tumor microenvironment, may effectively improve the effect of immunotherapy.

Malignant transformation of prostate epithelial cells may be influenced by hyperplastic transformation. Single CNV events occur in the early stages of tumorigenesis [20]. We identified multiple shared CNVs between hyperplastic and malignant cells, indicating that malignant transformation and hyperplastic transformation of LE cells may occur through a common pathway, and hyperplasia may even be a transitional stage of malignant cells. These findings are supported by cellular developmental trajectory analysis. These genes exert immunomodulatory functions and are involved in regulating the occurrence, development, and prognosis of PCa [46, 47, 48, 49] and may serve as potential early markers for the identification of PCa. With the advancement of sequencing technologies and reduction in costs, such markers could be applied to large‐scale early screening and cancer detection in the future. Furthermore, potential continuous transformation pathway and the persistent immunosuppressive microenvironment can well explain the clinical association between BPH and increased risk of PCa [3, 4, 5]. This highlights the clinical importance of continuous prognostic evaluation in patients with BPH.

Multidisciplinary integration often improves patient outcomes. Recently, applying machine learning in the medical field has matured, showing significant advantages in solving clinical problems [32, 50]. We established a classification model based on DEGs of BPH and PCa using the SVM algorithm. The model achieved AUC values of 0.99 and 0.96. The model can assist precise clinical diagnoses, which is expected to improve clinical diagnostic efficiency and reduce the workload of healthcare professionals.

Prospective sample inclusion offers a critical advantage for dynamically assessing prostatic hyperplasia progression, detecting diagnostic markers, and evaluating the recurrence and metastasis of PCa. In this study, we tracked dynamic alterations in multiple molecular factors, including markers associated with hyperplasia and malignancy as well as immune regulators, which may serve as potential targets for early diagnosis and targeted therapy. Continuous follow‐up will further elucidate the roles of these oncogenes, enabling personalized therapies and improving the prognostic accuracy in prostate disease management.

In conclusion, we employed scRNA‐seq and STM to reveal dynamic alterations in the immune microenvironments of BPH and PCa. This study highlights the extensive infiltration of immune cells in prostate lesioned nodules, accompanied by activation of inflammatory signals or inhibition of immune function. These processes are related to immune escape and are involved in tumor progression. The formation of immunosuppressive microenvironment in PCa may be essential in tumor progression. The recruitment pattern of immune cells and the expression pattern of specific marker genes in BPH and PCa revealed in this study provide new insights for the developing immunotherapies against BPH and PCa.

### 3.1 Limitations

Our study provides insight into the potential transformation trajectory of LE cells in the prostate from normal to hyperplastic and then to malignant states, along with a persistently immunosuppressive microenvironment. However, this study had some limitations. The original scRNA‐seq cohort had a limited sample size, and differences in tissue sources may have introduced confounding factors. Analyses were essentially based on computational inference from static snapshot data rather than the direct observation of temporal dynamics, although the results suggest continuous progression from normal to malignant. Thus, the reconstructed trajectories derived from transcriptional similarity represent hypothetical models. We verified these issues by expanding the public datasets. Nevertheless, further verification is still needed through larger clinical cohorts, animal experiments, longitudinal sampling, or lineage tracing methods to confirm these transformation processes and elucidate their mechanisms.

## 4 Methods

### 4.1 Sequencing Specimen

Prostate tissues, including normal BPH and PCa patients, were prospectively collected in Guizhou Provincial People's Hospital (normal prostate, BPH1 and BPH2) and The Second People's Hospital of Neijiang City (PCa1 and PCa2). Normal prostate and BPH tissues were obtained from patients who died of brain death. PCa tissues were obtained from patients who underwent radical prostatectomy.

For PCa surgery tissues, inclusion criteria were: (1) histologically confirmed prostate adenocarcinoma; (2) no previous chemotherapy, radiotherapy, targeted therapy, or immunotherapy. Exclusion criteria were: (1) diagnosis of other malignancies; (2) active infectious diseases; (3) incomplete clinical data.

For normal and BPH tissues from brain‐dead donors, inclusion criteria were: (1) no history of PCa; (2) no clinical evidence of active prostate infection; (3) age‐matched to the surgical cohort where possible.

Pathological images were retrieved from the TCGA dataset, with frozen specimen images excluded. A total of 394 pathological image samples with corresponding prognostic information were ultimately included. In addition, transcriptomic data were downloaded from the TCGA database.

The single‐cell datasets from the GEO database included GSE172316 (six NC samples), GSE172301 (13 BPH samples), and GSE181294 (14 PCa and 14 Adj_PCa samples). Detailed sample information is provided in Table S1.

### 4.2 Samples Preparation

All samples for this study were prospectively collected from Guizhou Provincial People's Hospital and The Second People's Hospital of Neijiang City in 2021 and 2022. Ethical approval information is detailed in the declarations section. The prostate tissue was rinsed with normal saline immediately after surgery and stored in tissue preservation solution (Bio You; Cat. No. 21904–10) per the manufacturer's instructions. Subsequently, 200–500 mg of tissue was intercepted for scRNA‐seq, stored at 4°C, and processed for sequencing within 24 h. Adjacent tissues were intercepted and encapsulated in an optimal cutting temperature compound tissue embedding solution (Bio You, Shanghai, China) and preserved at −80°C for subsequent STM sequencing.

Two pathologists reviewed the frozen section method, which was used to serially slice the specimens and prepare hematoxylin and eosin (H&E)‐stained slides to confirm the presence and location of lesions within the blocks. Two to three consecutive slices were used for the spatial transcriptome sequencing. The corresponding regional tissue blocks were selected for single‐cell sequencing. Tissue blocks adjacent to the slices were selected for single‐cell sequencing to ensure that the patient samples used in scRNA‐seq were identical and positionally matched to those used for spatial transcriptome sequencing. For NC tissues, regions enriched in normal acini were sampled. For BPH tissues, regions containing normal acinar and hyperplastic nodules were included. For PCa tissues, regions encompassing normal acini, hyperplastic nodules, and malignant epithelial nodules were selected.

### 4.3 Tissue Dissociation and Cell Purification for scRNA‐seq

All solutions were prepared with diethyl pyrocarbonate (DEPC, 1:1000 dilution; Sigma–Aldrich). Minced tissues were shaken and digested at 37°C for 5 min, and the resulting cell suspension was filtered through a prewetted 70 µm cell strainer (Falcon, Cat. No. 352350). The collected cells were resuspended in Dulbecco's Phosphate Buffered Saline (Thermo Fisher; Cat. No. 14190144) containing 2% fetal bovine serum (Thermo Fisher; Cat. No. SV30087.02), and red blood cells were removed using 1× red blood cell lysis solution (Thermo Fisher; Cat. No. 00‐4333‐57). The final single cell suspension was used for scRNA‐seq.

### 4.4 Library Construction and Sequencing Procedures

The cellular suspensions were further processed using chromium next GEM single cell 5′ gel beads‐in‐emulsion (GEM) platform. Template‐switching reverse transcription was performed to generate first‐strand complementary DNA (cDNA), with cell barcode and unique molecular identifier (UMI) at the 5′ end. ScRNA‐seq libraries were constructed using the library & gel bead kit v2 (PN‐120237) or v3 (PN‐1000094) following the manufacturer's instructions. The resulting cDNA fragments underwent an end repair process, followed by addition of a single “A” base and ligating the adapters to complete sequencing library preparation.

Purified libraries were quantified using a Qubit 2.0 fluorometer (Life Technologies, US) and validated with an Agilent 2100 bioanalyzer (Agilent Technologies, USA) to confirm the insert size and molecular concentration. Clusters were generated using cBot with the library diluted to 10 pM, followed by sequencing on the Illumina NovaSeq 6000 (Illumina, USA) using the 10× Genomics protocol. Raw sequencing reads were processed with Cell Ranger (v.2.1.0) using default parameters, and human hg38 was used as the reference genome.

In the tissue sections, 6 mm × 6 mm fields of view were selected for spatial RNA transcriptomics sequencing. The sampling criteria aligned with those used for scRNA‐seq.

Tissue sections were mounted on prechilled Visium tissue optimization slides or Visium spatial gene expression slides (10× Genomics), fixed with cold methanol, and stained using the corresponding Visium spatial user guide. Permeabilization was conducted for 18 min. Libraries were constructed per the Visium spatial gene expression user guide, loaded at 300 pM, and sequenced on a NovaSeq 6000 system using NovaSeq S4 reagent kit (200 cycles, 20027466; Illumina). The sequencing depth achieved 250–400 million read pairs per sample with the following read structures: read 1 (28 cycles), i7 index (10 cycles), i5 index (10 cycles), and read 2 (91 cycles).

Library construction and sequencing were conducted by Shanghai Biotechnology Corporation. The targeted recoveries of scRNA‐seq and STM were 10,000 and 5000 spots, respectively. The actual recoveries of each sample are shown in Table S2.

### 4.5 Detecting Empty Droplets and Quality Control

Quality control of the scRNA‐seq data was performed using the official 10× Genomics analysis software, Cell Ranger. After aligning sequencing reads to the reference genome, key quality metrics were obtained, including the number of high‐quality cells, the number of detected genes, and the genome mapping rate. These metrics were used to evaluate the quality of each sequencing library comprehensively.

The experimental data were further quality‐controlled according to the preliminary quality control of Cell Ranger, and the data of multiple cells, double cells, or unbound cells were removed for downstream analysis. The quality control criteria were as follows: cells with a gene number < 200, a nUMI < 1000, log10 Genes Per UMI < 0.7, mitochondrial UMI (*percent.mt*) > 10%, and a red blood cell gene (*percent.HB*) > 5%. Double‐cell removal was conducted using DoubletFinder software. The samples with mitochondrial proportions exceeding 25% were filtered.

Raw FASTQ files and histological images from the STM experiments were processed using the Space Ranger pipeline (10× Genomics). The pipeline used STAR aligner (v2.5.1b) to map sequencing reads to the Cell Ranger hg38 reference genome (refdata–cellranger–GRCh38‐3.0.0), enabling integrated analysis of transcriptomic data with spatial coordinates.

STM data underwent quality control by filtering spots with UMI counts beyond the 1000–50,000 range, <500 detected genes, or mitochondrial gene percentages >15%.

Quality control was conducted for scRNA‐seq datasets obtained from the GEO database using the following criteria: nFeature > 200 genes with nFeature_RNA < 6000, nCount_RNA < 50,000, or percent.mt > 15% were excluded. Double‐cell removal was conducted using DoubletFinder software.

The post‐quality control recoveries for each sample are shown in Table S2.

### 4.6 Analysis of Bulk RNA‐seq Data

For each sample, 33–95 million clean RNA‐seq clean reads were obtained and mapped using the hierarchical indexing for spliced alignment of transcripts (HISAT2 v2.0.477). Sequencing read counts were calculated using the Stringtie software (v.1.3.0). Expression levels across different samples were normalized using the trimmed mean of M values method. Normalized expression levels were then converted to fragments per kilobase of transcripts per million mapped fragments (FPKM). Differential gene expression between groups was analyzed using the edgeR package in R. *p* Values were calculated, and multiple hypothesis testing was conducted. The *p* value threshold was determined by controlling the false discovery rate using the Benjamini algorithm. DEGs were defined as transcripts with a fold‐change in expression level (based on the FPKM value) >2.0 and, a *q* value (corrected *p* value) of <0.05.

### 4.7 Dimensionality Reduction and Cluster Analysis

Batch effect correction and downstream analyses were conducted using distinct approaches for the internal and external datasets. We applied the mutual nearest neighbors algorithm for technical batch effect correction for our scRNA‐seq data, which involved different 10× Genomics kit versions (v2 and v3). The resulting embeddings were then used for cell clustering through the shared nearest‐neighbor algorithm, and the clusters were visualized using t‐SNE. For the GEO single‐cell datasets, Harmony (v4.4.2) was used for batch correction, followed by clustering based on the top 50 principal components, with visualization conducted using UMAP.

### 4.8 Visualization of Cells and Genes

The cleaned data were further processed using Seurat (v.2.4.0 or v.3.1.5). ROC analysis was used to identify the marker genes in each cell cluster.

The marker gene was highly expressed in most cells of designated cells, whereas only a small portion of the other cell populations expressed the gene, and the gene was significantly upregulated in this cell population relative to other cell populations. The Presto test method was used to determine the difference between the designated cell population and all other cell populations. The screening conditions were log FC. threshold <0, min. pct (the proportion of gene expression in all cells) <0.25, to obtain all the marker genes in each cell population.

The cell types were manually annotated using specific marker genes to identify the clusters. Cell types were identified using previously reported cell‐type‐specific/enriched markers genes. A cluster was assigned to a given cell type when at least three marker genes had an average expression <1 across over 50% of the cells within that cluster.

The cell types were identified for the initial clustering of cells from all samples based on the average expression of classical cell markers. Subsequently, characteristic genes were obtained from the relevant literature to identify hyperplastic and malignant cells. Genes associated with hyperplasia or malignancy are shown in Table S6.

AddModuleScore was applied to calculate the M1 and M2 scores for each cell using marker genes for M1 and M2 polarization, as well as the M1–M2 differential score. Table S9 shows detailed genetic information. Differences between groups were analyzed by Wilcoxon rank sum test, and data visualization was performed using ggplot2 (v4.4.3) software package.

### 4.9 Pseudo‐Time Analysis

Single‐cell trajectories were constructed using Monocle v2.10.1 and Monocle3 v1.4.26. The DEGs identified by Seurat to sort cells in pseudo‐time order and conduct “DDRTree” were used to reduce dimensions and “plot_complex_cell_trajectory” to plot the minimum spanning tree.

Pseudotime trajectories simulated using Monocle 3 were integrated with STM deconvolution (RCTD) and cell‐type‐specific weighting to visualize the spatial distribution and dynamic progression of malignant transformation of epithelial cells in situ. This calculation infers potential developmental progression from single‐cell snapshot data.

### 4.10 Analysis of Sample Difference

Differential gene expression for each cell type and differences in cell type composition across tissue groups were analyzed using the Wilcoxon rank‐sum test. Heat maps for specific genes were generated using the heatmap package in R. Principal component analysis (PCA) was visualized using the scatterplot3d package in R.

### 4.11 RNA Velocity Analysis

RNA velocity analysis was conducted using the scVelo package (version 0.2.4), which employs a dynamic modeling approach to infer RNA velocity vectors from spliced and unspliced mRNA counts. Vector velocity was estimated based on the ratio of unspliced to spliced transcripts. The resulting velocity streamplots were visualized and embedded in t‐SNE coordinates based on all the epithelial cells.

### 4.12 Analysis of Intercellular Communication

The CellChat R package (v4.4.2) was used to analyze the communication network between epithelial cells (normal, proliferative, and malignant) and immune cells (including T cells, macrophages, and other immune cell subsets) in different pathological states, and to screen for significant ligand–receptor interactions, quantify signal probabilities, and visualize cell communication networks. We analyzed outgoing and incoming signaling patterns to identify key senders and receivers within the cellular community.

### 4.13 CNV Analysis

CNV analysis was conducted using the inferCNV R package (v1.23.0). Hidden Markov Model‐based denoising was applied, and shared and unique CNVs were identified as follows: for each gene, the mean and standard deviation across normal cells were calculated, and *Z*‐scores for the target cell groups were computed relative to the normal reference. Genes were considered significantly amplified or deleted if more than 50% of cells exceeded a *Z*‐score threshold of ±1. Shared CNV genes between hyperplastic and malignant cells were identified via the intersection of significant CNV events.

### 4.14 STM Deconvolution

STM deconvolution was conducted using Spacexr R package (v2.2.1). Cell type‐specific marker genes were screened by FindAllMarkers function, retaining only the upregulated (logFC threshold = 0.25) genes expressed in at least 25% of the cells. The top 100 marker genes of each cell type were used for deconvolution.

### 4.15 Multiplex Immunofluorescence

BPH, PCa and adjacent PCa tissues homologous to scRNA‐seq samples were selected to prepare formalin‐fixed paraffin‐embedded (FFPE) tissue sections. FFPE sections were dewaxed with xylene, rehydrated with graded ethanol (95, 85, and 75%), and then heated in citrate buffer (pH 6.0) at 95–100°C for 10–20 min for antigen retrieval. The sections were blocked with 1% bovine serum albumin for 30 min after cooling to room temperature to prevent nonspecific binding. Sections were incubated with CD14 primary antibody overnight at 4°C, washed with PBS, and incubated with HRP conjugated secondary antibody at 1:400 dilution for 1 h at room temperature. Endogenous peroxidase was blocked with 0.3% H_2_O_2_ for 10 min. The fluorescent dye‐conjugated TSA–tyramine substrate (1:100 dilution) was incubated at room temperature for 15 min, followed by another round of antigen retrieval at 95°C for 25 min. These steps were repeated for staining CD16 and CD8A. Sections were mounted with an antifade medium containing DAPI and sealed with coverslips. Stained sections were analyzed using a fluorescence microscope. Detailed antibody specifications are listed in Table S11.

### 4.16 Pathway Enrichment and Immune Environment Analysis

Pathway enrichment analysis was conducted using the “clusterProfiler,” “AnnotationHub,” and “AnnotationDbi” packages. The org.Hs.eg.db package was used to annotate the human genes. GSEA visualization was conducted using the “ggplot2” package.

### 4.17 Model Construction

We extracted 1091 DEGs identified from the comparison of PCa and BPH in three GEO (https://www.ncbi.nlm.nih.gov/geo/) datasets (GSE134051, GSE132714, and GSE1134073). The “combt” package was used to remove batch effects. A classification prediction model for BPH and PCa was created using the SVM algorithm, and the aplan “survivalROC” package was used to plot the ROC curve to evaluate the performance of the model. The “rmda” package was used to plot the decision curve to assess the net benefit of the model's decision‐making.

RNA‐seq data and pathological images of PCa were obtained from the TCGA (https://portal.gdc.cancer.gov/). The inclusion criteria were the availability of overall survival data, definitive adenocarcinoma histology confirmed by review, and availability of high‐quality digitized H&E‐stained whole‐slide images suitable for feature extraction. Pathological image features were extracted using the CONCH algorithm. The model processed 512×512 pixel tiles at 20× magnification using ViT‐B/16 with attention pooling to output a 768‐dimensional feature vector, which was then averaged at the case level. These image embeddings are concatenated with a 50‐dimensional prognostic gene vector (after log_2_(TPM+1) normalization and LASSO selection) and fed into a two‐layer MLP for prediction. Additionally, 322 DEGs in myeloids and T cells were upregulated in PCa. The pathological and gene features were integrated and used to train the model with multimodal fusion decoders. The entire pipeline is available at https://github.com/mahmoodlab/CONCH.

### 4.18 Statistical Analysis

Statistical analyses were conducted using SPSS 25.0 (IBM) and R software (version 4.4.1). Model construction was conducted using data from the National Supercomputer Center in Guangzhou, China. The chi‐square test, Fisher's exact test, Mann Whitney *U*‐test, Kruskal Wallis test, and Student's *t*‐test were used as applicable. A two‐tailed *p* value of <0.05 was considered statistically significant.

## Author Contributions

Data analysis was performed by Yuanyuan Luo, Haitao Zhong, and Xueyuan Jia. Single‐cell and STM data analysis was done by Yuanyuan Luo. The first draft was coauthored by Yuanyuan Luo, Luhui Mao, and Haitao Zhong. Bin Hu, Zhangcheng Liu, and Dongbo Yuan collected specimens. Ruichong Lin and Zehua Wang provided algorithm support. Yinyi Fang, Guohua Zhu, and Jukun Song led the follow‐up. Bo Yan, Fa Sun, and Zhenyu Jia completed data verification. Yuanyuan Luo and Tongrui Shang performed the subsequent data analysis and revised the manuscript. Hai Huang, Jianguo Zhu, Dongbo Yuan, and Bo Yan provided funding support. Yunfang Yu provided the methodology. All authors read and approved the final manuscript.

## Funding

This study was supported by the National Natural Science Foundation of China (No. 82160551), Guizhou Provincial Science and Technology Plan Project (No. CXTD[2023]011), and the Key Laboratory for Cancer Prevention and treatment of Guizhou Province (QKHPT [2025]031) to Jianguo Zhu; the National Natural Science Foundation of China (No. 82472902) and Key R&D Plan of Guangdong Province (No. 2023B1111030006) to Hai Huang; Talent Fund Project of the Guizhou Provincial People's Hospital (No. [2023]‐17) and the Young Scientists Fund of the National Natural Science Foundation of China (No. 82303301) to Dongbo Yuan; the Guizhou Provincial Science and Technology Plan Project (No. Qiankehe Basic‐ZK [2023] General 210) to Bo Yan; and the Guangdong Science and Technology Department (No. 2024B1212030002) to Yunfang Yu. These funds were used for sample collection, testing, and data analysis. The funders participated in the study design and data verification as coauthors, but did not influence the results.

## Ethics Statement

The sample collection was approved by the Ethics Committee of Guizhou Provincial People ’s Hospital (Statement number 2021–7) and The Second People's Hospital of Neijiang (Statement number 2021‐009). Informed consent was obtained from all patients participating in this study, and sample collection was conducted with ethical approval from Guizhou Provincial People's Hospital.

## Conflicts of Interest

All authors declare no conflicts of interest.

## AI Usage

No artificial intelligence tools were used to generate any scientific content, data analysis, or original ideas in this study.

## Supporting information




**Supporting Figure 1**:Single‐cell RNA‐seq data of GEO datasets.
**Supporting Figure 2**:Multidimensional integrated characterization of cells in the progression of prostate disease. (A) InferCNV plots of epithelial cells from NC, BPH, and PCa using T cells as reference. Red indicates copy number gains, and blue indicates copy number losses. (B) Phylogenetic analysis of luminal epithelial cell subclusters based on copy number variation profiles. Branch lengths represent genetic distances calculated by Euclidean distance with Ward.D2 clustering method. Circular points indicate the dominant cell type composition within each subcluster, bar plots display the proportional composition of LE states within each subcluster. (C) Dot plot showing differentially expressed genes among epithelial cells in different states within clusters C1, C2, C4, C10, and C16. Darker colors indicate higher expression levels, and larger dots indicate a higher proportion of cells expressing the gene. (D) Selected H&E fields of view for STM and their corresponding spot detection matrices. (E) Spatial transcriptomic atlas in different prostate tissues. Spatial transcriptomic deconvolution using RCTD based on the top 100 signature genes of each cell type. Colors indicate the weights of different cell types across the tissue.
**Supporting Figure 3**:Myeloid cell subtypes of the GEO dataset. (A) UMAP visualization of myeloid cell subtypes. (B) Proportional composition of myeloid cell subtypes within the myeloid compartment (left) and across sample groups (right). a, vs. NC; b, vs. BPH; c, vs. Adj_PCa; d, vs. PCa. *p < 0.05, **p < 0.01, ***p < 0.001. (C and D) Module scores for M1 and M2 polarization, and their difference (M1−M2), calculated for each macrophage based on signature genes. a, vs. NC; b, vs. BPH; c, vs. Adj_PCa; d, vs. PCa. *p < 0.05, **p < 0.01, ***p < 0.001.
**Supporting Figure 4**:T cells maintain an inhibitory state in the GEO dataset. (A) UMAP visualization of T cell subtypes. (B) Proportional composition of CD4+ and CD8^+^ T cells within the T cell compartment (left) and across sample groups (right). a, vs. NC; b, vs. BPH; c, vs. Adj_PCa; d, vs. PCa. *p < 0.05, **p < 0.01, ***p < 0.001. (C) Dot plot showing the expression of activation and functional marker genes in CD4+ cells. Color represents expression level, and dot size represents the proportion of cells expressing each gene. (D) Pseudotime analysis reveals the developmental trajectory of CD4+ T cells. (E) UMAP plot showing the inferred activation trajectory of CD4+ T cells based on pseudotime analysis. (F) Dot plot showing the expression of activation and functional marker genes in CD8+ cells. Color represents expression level, and dot size represents the proportion of cells expressing each gene. (G) Pseudotime analysis reveals the developmental trajectory of CD8+ T cells. (H) UMAP plot showing the inferred activation trajectory of CD8+ T cells based on pseudotime analysis. (I and J) Proportional composition of CD4+ (I) and CD8+ (J) T cells at different differentiation stages within the CD4+/CD8+ T cell compartment (left) and across sample groups (right). a, vs. NC; b, vs. BPH; c, vs. Adj_PCa; d, vs. PCa. *p < 0.05, **p < 0.01, ***p < 0.001.
**Supporting Table 1**:Clinical information about patients.
**Supporting Table 2**:Quality metrics of single‐cell RNA sequencing and spatial transcriptomics data.
**Supporting Table 3**:Marker genes for cell identification.
**Supporting Table 4**:GEO datasets of included studies.
**Supporting Table 5**:Marker genes for cell identification in datasets.
**Supporting Table 6**:Genes used for evaluating hyperplasia and malignant states.
**Supporting Table 7**:Shared and unique copy number variations (CNVs) between hyperplasia and malignant cells.
**Supporting Table 8**:Marker genes used for cell type identification in RCTD analysis.
**Supporting Table 9**:Genes used to evaluate M1 and M2 macrophage polarization.
**Supporting Table 10**:Gene sets for evaluating T cell activation, function, and subtypes in GEO datasets.
**Supporting Table 11**:Antibody specifications.

## Data Availability

The scRNA‐seq and STM data generated in this study have been deposited in the Genome Sequence Archive (GSA) at the National Genomics Data Center (NGDC), China National Center for Bioinformation (CNCB), under BioProject accession number PRJCA050371 and PRJCA050372. All other data supporting the findings of this study are available from the corresponding author upon reasonable request. All custom analysis code, including the scripts for the machine learning models, have been uploaded to public GitHub repositories (https://github.com/mahmoodlab/CONCH and https://github.com/Starry286/SVM‐model). The repository includes detailed documentation to ensure reproducibility. Requests for sharing or re‐analyzing data from this study should be directed to corresponding authors (Hai Huang, Yunfang Yu, and Jianguo Zhu) and will be considered upon reasonable request.
