# Supplementary material for: Single‐Cell and Spatial Transcriptomics Uncover Immune Dynamics and Cellular Heterogeneity in Benign Prostatic Hyperplasia and Prostate Cancer Transition
Source: MedComm (2020). 2026 May 23;7(6):e70760. doi: 10.1002/mco2.70760 (PMC13240583; doi:10.1002/mco2.70760)
Supplement: Supplementary file 1 — Supporting Figure 1:Single‐cell RNA‐seq data of GEO datasets. Supporting Figure 2:Multidimensional integrated characterization of cells in the progression of prostate disease. (A) InferCNV plots of epithelial cells from NC, BPH, and PCa using T cells as reference. Red indicates copy number gains, and blue indicates copy number losses. (B) Phylogenetic analysis of luminal epithelial cell subclusters based on copy number variation profiles. Branch lengths represent genetic distances calculated by Euclidean distance with Ward.D2 clustering method. Circular points indicate the dominant cell type composition within each subcluster, bar plots display the proportional composition of LE states within each subcluster. (C) Dot plot showing differentially expressed genes among epithelial cells in different states within clusters C1, C2, C4, C10, and C16. Darker colors indicate higher expression levels, and larger dots indicate a higher proportion of cells expressing the gene. (D) Selected H&E fields of view for STM and their corresponding spot detection matrices. (E) Spatial transcriptomic atlas in different prostate tissues. Spatial transcriptomic deconvolution using RCTD based on the top 100 signature genes of each cell type. Colors indicate the weights of different cell types across the tissue. Supporting Figure 3:Myeloid cell subtypes of the GEO dataset. (A) UMAP visualization of myeloid cell subtypes. (B) Proportional composition of myeloid cell subtypes within the myeloid compartment (left) and across sample groups (right). a, vs. NC; b, vs. BPH; c, vs. Adj_PCa; d, vs. PCa. *p < 0.05, **p < 0.01, ***p < 0.001. (C and D) Module scores for M1 and M2 polarization, and their difference (M1−M2), calculated for each macrophage based on signature genes. a, vs. NC; b, vs. BPH; c, vs. Adj_PCa; d, vs. PCa. *p < 0.05, **p < 0.01, ***p < 0.001. Supporting Figure 4:T cells maintain an inhibitory state in the GEO dataset. (A) UMAP visualization of T cell subtypes. (B) Proportional com [file MCO2-7-e70760-s001.docx]

**Single-Cell and Spatial Transcriptomics Uncover Immune Dynamics and Cellular Heterogeneity in Benign Prostatic Hyperplasia and Prostate Cancer Transition**

Yuanyuan Luo^1, 2, #^, Haitao Zhong^3, #^, Tongrui Shang^3, #^, Bin Hu^4^, Dongbo Yuan^1^, Xueyuan Jia^5^, Ruichong Lin^6, 7^, Zehua Wang^7^, Yinyi Fang^1^, Guohua Zhu^1^, Jukun Song^8^, Zhangcheng Liu^9^, Bo Yan^1^, Fa Sun^1^, Zhenyu Jia^10^, Yunfang Yu^3, 11, 12, 13*^, Luhui Mao^3, *^, Hai Huang^3*^, Jianguo Zhu^1, 2, 14*^

^1^ Department of Urology, Guizhou Provincial People’s Hospital, Guiyang, Guizhou, China.

^2^ GuiZhou University Medical College, Guiyang 550025, Guizhou Province, China.

^3^ Guangdong Provincial Key Laboratory of Malignant Tumor Epigenetics and Gene Regulation, Department of Medical Oncology, Sun Yat-sen Memorial Hospital, Sun Yat-sen University, Guangzhou, China.

^4^ Department of Urology, Kweichow Moutai Hospital, Guizhou, China.

^5^ School of Pharmacy, Faculty of Medicine, State Key Laboratory of Quality Research in Chinese Medicines, Macau University of Science and Technology, Taipa, Macau, China

^6^ Faculty of Innovation Engineering, Macau University of Science and Technology, Taipa, Macao, China.

^7^ School of Computer and Information Engineering, Guangzhou Huali College, Guangzhou, China.

^8^ Department of Stomatology, Guizhou Medical University Stomatological Hospital, Guizhou, China.

^9^ Department of Urology, The second People’s Hospital of Neijiang, Sichuan, China.

^10^ Department of Plant and Botany Sciences, University of California of Riverside, Riverside, CA 92521, USA.

^11^ Faculty of Medicine and Artificial Intelligence Cross Disciplinary Research Institute, Macau University of Science and Technology, Macau, China.

^12^ Institute of Health Medicine, Southern University of Science and Technology.

^13^ Guangdong Provincial Key Laboratory of Cancer Pathogenesis and Precision Diagnosis and Treatment, AI Big Data Laboratory, Shenshan Medical Center, Memorial Hospital of Sun Yat-sen University, Shanwei, China.

^14^ Key Laboratory for Cancer Prevention and treatment of Guizhou Province.

^#^ These authors were considered co-first authors.

* These authors were considered joint correspondence authors.

**Correspondence:**

[doctorzhujianguo@163.com;](mailto:jgzhu@gzu.edu.cn;) [yuyf9@mail.sysu.edu.cn;](mailto:yuyf9@mail.sysu.edu.cn;) [huangh9@mail.sysu.edu.cn](mailto:huangh9@mail.sysu.edu.c;)

**Supplementary Figures:**


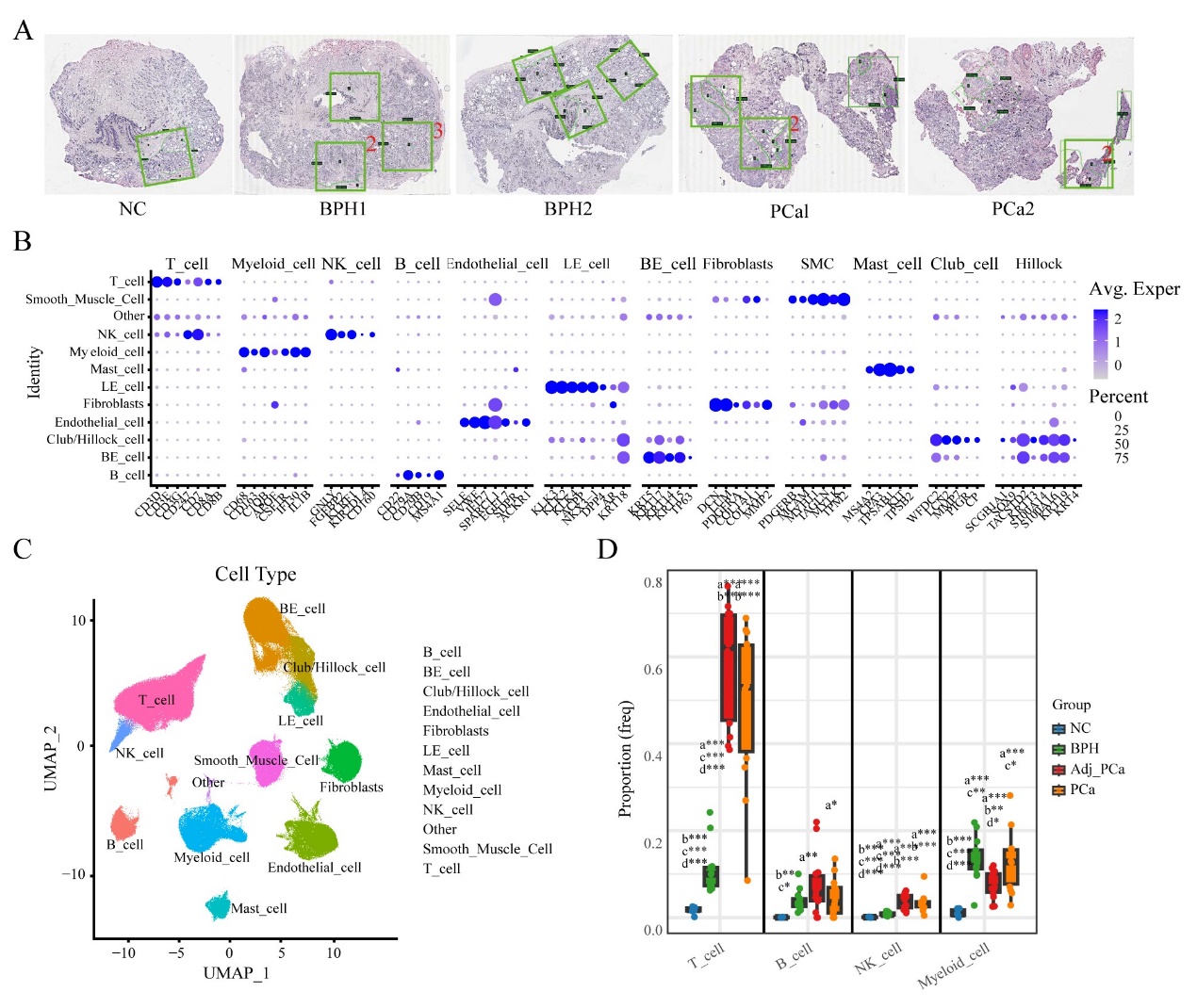


Figure S1. single-cell RNA-seq data of GEO datasets.

**A. H&E-stained section with the green box outlining the target field for STM.**

B. Dot plot showing the expression of key marker genes (see Supplement Table 5) across major cell types. Darker colors indicate higher expression levels, and larger dots indicate a higher proportion of cells expressing the gene.

C. UMAP visualization of cell types.

D. Immune cell composition across sample groups from GEO datasets. a, vs NC; b, vs BPH; c, vs Adj_PCa; d, vs PCa. * p < 0.05, ** p < 0.01, *** p < 0.001.


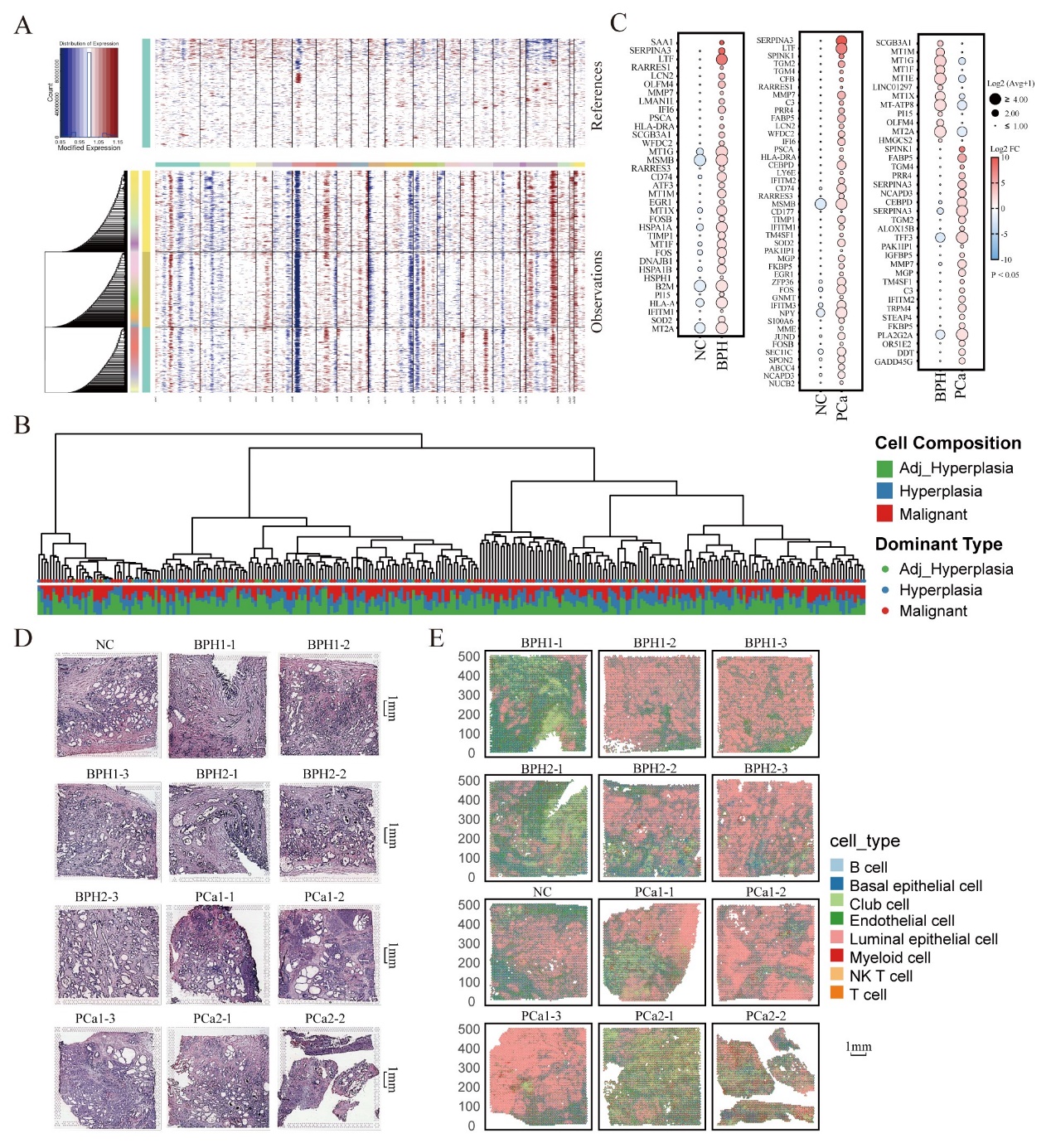


Figure S2. Multi-dimensional integrated characterization of cells in the progression of prostate disease.

A. InferCNV plots of epithelial cells from NC, BPH, and PCa using T cells as reference. Red indicates copy number gains, and blue indicates copy number losses.

B. Phylogenetic analysis of luminal epithelial cell subclusters based on copy number variation profiles. Branch lengths represent genetic distances calculated by Euclidean distance with Ward.D2 clustering method. Circular points indicate the dominant cell type composition within each subcluster, bar plots display the proportional composition of LE states within each subcluster.

C. Dot plot showing differentially expressed genes among epithelial cells in different states within clusters C1, C2, C4, C10, and C16. Darker colors indicate higher expression levels, and larger dots indicate a higher proportion of cells expressing the gene.

D. Selected H&E fields of view for STM and their corresponding detection matrices.

E. Spatial transcriptomic atlas in different prostate tissues. Spatial transcriptomic deconvolution using RCTD based on the top 100 signature genes of each cell type. Colors indicate the weights of different cell types across the tissue.


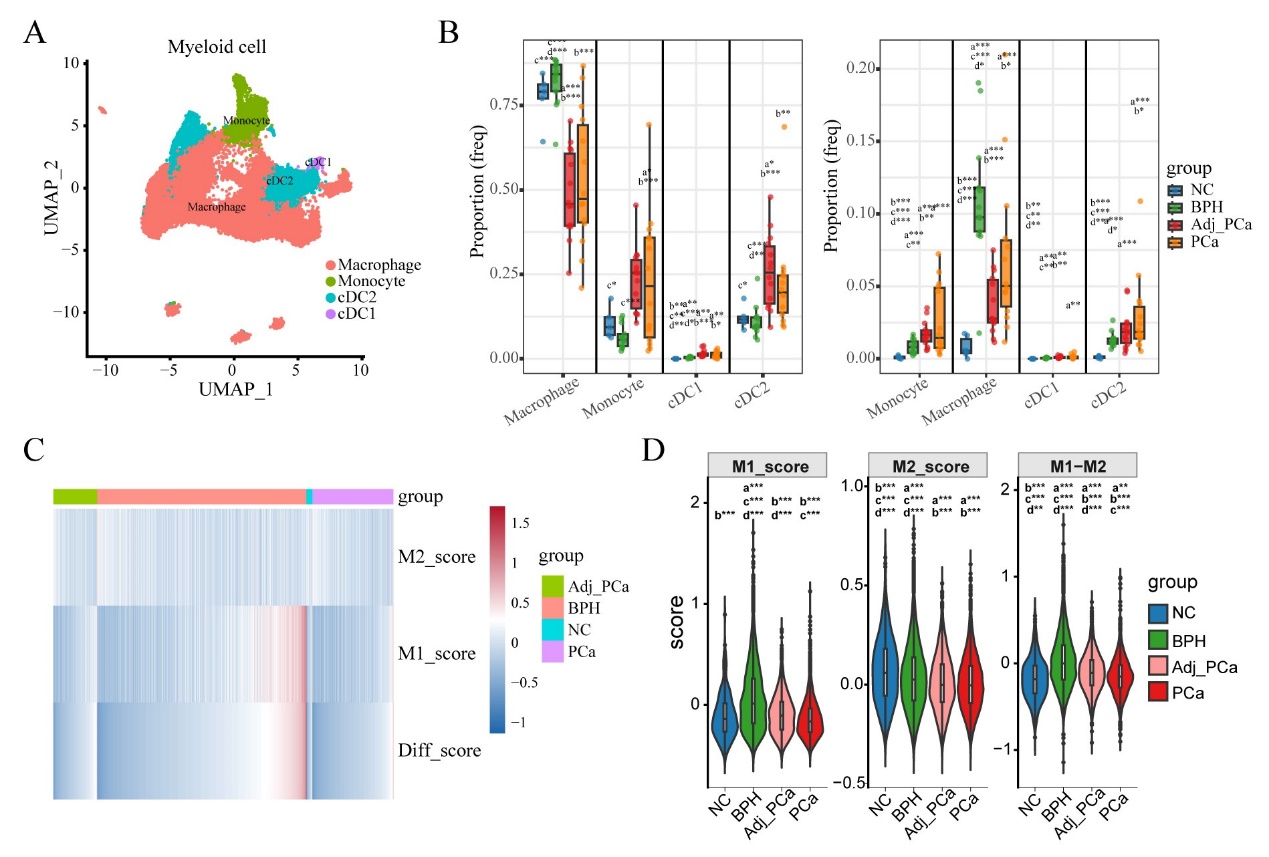


Figure S3. Myeloid cell subtypes of the GEO dataset.

A. UMAP visualization of myeloid cell subtypes.

B. Proportional composition of myeloid cell subtypes within the myeloid compartment (left) and across sample groups (right). a, vs NC; b, vs BPH; c, vs Adj_PCa; d, vs PCa. * p < 0.05, ** p < 0.01, *** p < 0.001.

C, D. Module scores for M1 and M2 polarization, and their difference (M1 − M2), calculated for each macrophage based on signature genes. a, vs NC; b, vs BPH; c, vs Adj_PCa; d, vs PCa. * p < 0.05, ** p < 0.01, *** p < 0.001.


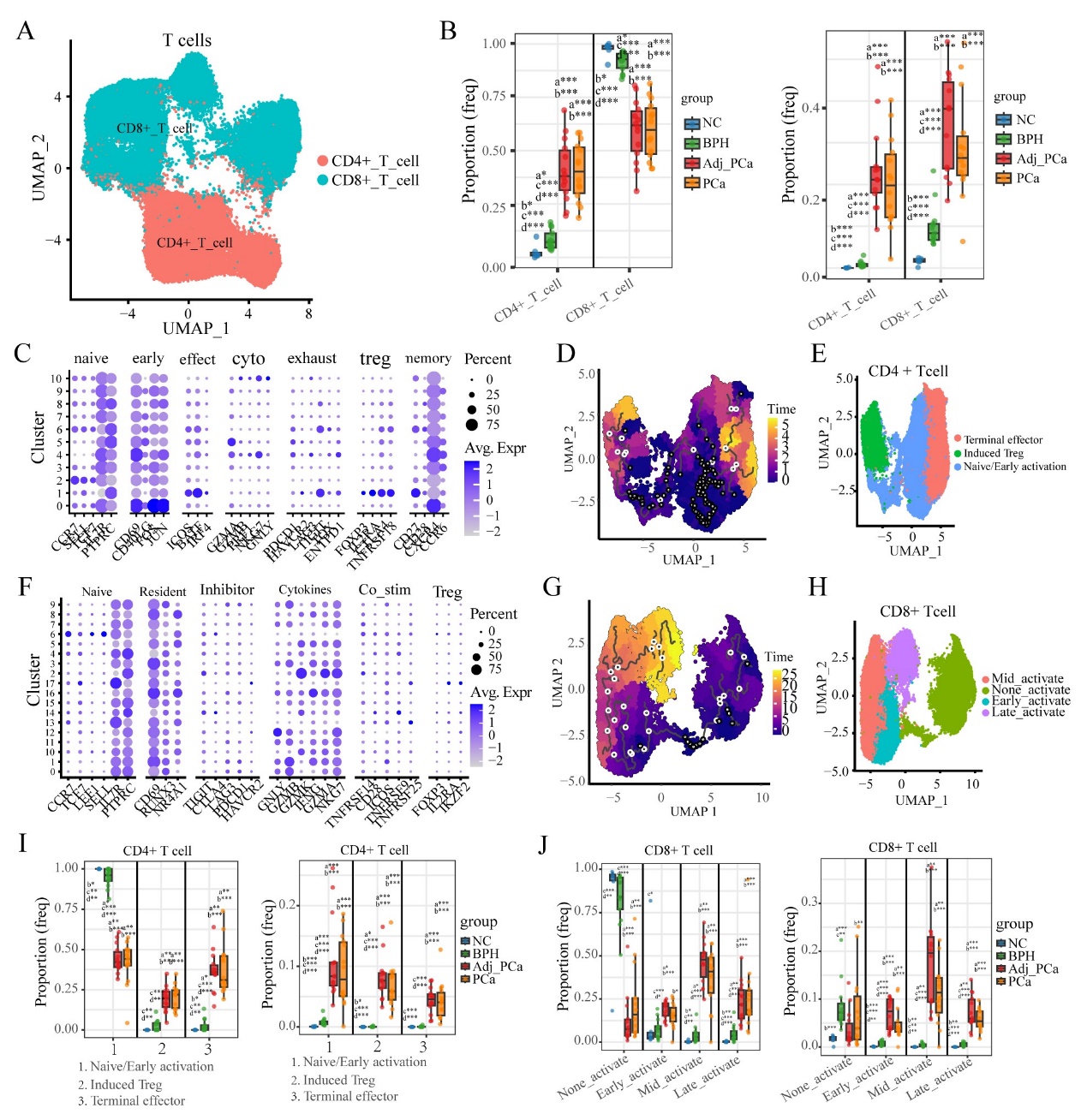


Figure S4. T cells maintain an inhibitory state in the GEO dataset.

A. UMAP visualization of T cell subtypes.

B. Proportional composition of CD4+ and CD8+ T cells within the T cell compartment (left) and across sample groups (right). a, vs NC; b, vs BPH; c, vs Adj_PCa; d, vs PCa. * p < 0.05, ** p < 0.01, *** p < 0.001.

C. Dot plot showing the expression of activation and functional marker genes in CD4+ cells. Color represents expression level, and dot size represents the proportion of cells expressing each gene.

D. Pseudotime analysis reveals the developmental trajectory of CD4+ T cells.

E. UMAP plot showing the inferred activation trajectory of CD4+ T cells based on pseudotime analysis.

F. Dot plot showing the expression of activation and functional marker genes in CD8+ cells. Color represents expression level, and dot size represents the proportion of cells expressing each gene.

G. Pseudotime analysis reveals the developmental trajectory of CD8+ T cells.

H. UMAP plot showing the inferred activation trajectory of CD8+ T cells based on pseudotime analysis.

I, J. Proportional composition of CD4+ (I) and CD8+ (J) T cells at different differentiation stages within the CD4+/CD8+ T cell compartment (left) and across sample groups (right). a, vs NC; b, vs BPH; c, vs Adj_PCa; d, vs PCa. * p < 0.05, ** p < 0.01, *** p < 0.001.

Supplementary Tables:

Table S1. Clinical information about patients.

| Patient | Age | Samples | Stage | PSA | Gleason Score |
| --- | --- | --- | --- | --- | --- |
| Normal | 36 | Normal | \ | \ | \ |
| BPH1 | 56 | BPH1 | \ | \ | \ |
| BPH2 | 56 | BPH2 | \ | \ | \ |
| PCa1 | 57 | Adjacent | pT2cNxMx | 12.79 | 3+4 |
|  |  | Tumor |  |  |  |
| PCa2 | 57 | Adjacent | pT2aNxMx | 10.42 | 3+5 |
|  |  | Tumor |  |  |  |

Table S2. Quality metrics of single-cell RNA sequencing and spatial transcriptomics data.

A. Single-cell RNA sequencing data.

| Patients | Tissue | Cell Count (Raw) | Gene Median (Raw) | UMI Mean (Raw) | Cell Count (QC) | Gene Median (QC) | UMI Mean (QC) |
| --- | --- | --- | --- | --- | --- | --- | --- |
| Normal | NC | 9,431 | 1,795 | 43,380 | 7815 | 1859 | 5034 |
| BPH | BPH1 | 3,951 | 1,072 | 138,368 | 2021 | 1431 | 3938 |
|  | BPH2 | 11,765 | 1,512 | 42,360 | 8511 | 1539 | 4641 |
| PCa | Adj-PCa1 | 4,069 | 1,610 | 83,533 | 2763 | 1967 | 6127 |
|  | PCa1 | 10,478 | 1,477 | 33,082 | 7671 | 1584 | 4955 |
|  | Adj-PCa2 | 10,326 | 1,539 | 35,855 | 8595 | 1434 | 4433 |
|  | PCa2 | 10,764 | 1,455 | 40,570 | 8387 | 1521 | 5264 |

B. Spatial transcriptomics data.

| Patients | Chips | Spot Count (Raw) | Gene Median (Raw) | UMI Mean (Raw) | Spot Count (QC) | Gene Median (QC) | UMI Mean (QC) |
| --- | --- | --- | --- | --- | --- | --- | --- |
| Normal | NC | 4650 | 2222 | 5996.611 | 4650 | 2352 | 6469 |
| BPH1 | BPH1-1 | 4657 | 2397 | 5822.570 | 4657 | 2487 | 6131 |
|  | BPH1-2 | 4700 | 2708 | 6977.360 | 4700 | 2802 | 7271 |
|  | BPH1-3 | 4660 | 2712.5 | 7834.273 | 4660 | 2803 | 8173 |
| BPH2 | BPH2-1 | 4633 | 2632 | 7902.605 | 4633 | 2995 | 9336 |
|  | BPH2-2 | 4662 | 2588 | 7292.577 | 4662 | 2812 | 8123 |
|  | BPH2-3 | 4652 | 2519 | 7335.565 | 4652 | 2697 | 8019 |
| PCa1 | PCa1-1 | 4050 | 3099 | 7636.258 | 4050 | 3304 | 8324 |
|  | PCa1-2 | 4670 | 2818.5 | 8447.978 | 4670 | 2818 | 8448 |
|  | PCa1-3 | 4628 | 2847.5 | 8021.420 | 4628 | 3069 | 8918 |
| PCa2 | PCa2-1 | 4385 | 2320 | 5828.725 | 4385 | 2401 | 6076 |
|  | PCa2-2 | 3054 | 2398 | 6562.994 | 3054 | 2630 | 7386 |

C. Single-cell RNA sequencing of GEO data.

| Patients | Tissue | Cell Count (Raw) | Gene Median (Raw) | UMI Mean (Raw) | Cell Count (QC) | Gene Median (QC) | UMI Mean (QC) |
| --- | --- | --- | --- | --- | --- | --- | --- |
| Normal | NC1 | 9474 | 1546 | 7231 | 9306 | 1548 | 6942 |
|  | NC2 | 6521 | 1708 | 7926 | 6419 | 1710 | 7678 |
|  | NC3 | 7879 | 1316 | 4698 | 7595 | 1333 | 4831 |
|  | NC4 | 7844 | 1178 | 4099 | 7737 | 1183 | 4145 |
|  | NC5 | 7230 | 1672 | 6658 | 5837 | 1846 | 7839 |
|  | NC6 | 7451 | 1725 | 6786 | 6246 | 1874 | 7779 |
| BPH | BPH1 | 5883 | 1378 | 5121 | 4772 | 1616 | 5977 |
|  | BPH2 | 3081 | 865 | 3097 | 3011 | 882 | 3147 |
|  | BPH3 | 5992 | 1428 | 4843 | 5566 | 1494 | 5138 |
|  | BPH4 | 8873 | 1248 | 3984 | 8822 | 1254 | 4001 |
|  | BPH5 | 8882 | 1668 | 6084 | 7858 | 1780 | 6726 |
|  | BPH6 | 7927 | 1341 | 5278 | 7691 | 1364 | 5403 |
|  | BPH7 | 7982 | 1297 | 6194 | 6917 | 1413 | 5889 |
|  | BPH8 | 17453 | 1106 | 3771 | 15313 | 1171 | 3850 |
|  | BPH9 | 12781 | 1443 | 4943 | 11847 | 1473 | 5007 |
|  | BPH10 | 8673 | 1759 | 6742 | 8217 | 1777 | 6636 |
|  | BPH11 | 8460 | 1529 | 6416 | 5531 | 1828 | 7153 |
|  | BPH12 | 8597 | 1505 | 5798 | 6740 | 1727 | 6201 |
|  | BPH13 | 10818 | 1418 | 4761 | 8515 | 1582 | 5392 |
| PCa | Adj_PCa1 | 7720 | 752 | 2144 | 7720 | 752 | 2144 |
|  | PCa1 | 3745 | 836 | 2385 | 3745 | 836 | 2385 |
|  | Adj_PCa2 | 4424 | 934 | 2806 | 4424 | 934 | 2806 |
|  | PCa2 | 3781 | 1076 | 4012 | 3778 | 1076 | 3966 |
|  | Adj_PCa3 | 2452 | 836 | 2829 | 2452 | 836 | 2829 |
|  | PCa3 | 4888 | 772 | 2613 | 4888 | 772 | 2613 |
|  | Adj_PCa4 | 2519 | 634 | 2044 | 2519 | 634 | 2044 |
|  | PCa4 | 1939 | 802 | 2556 | 1939 | 802 | 2556 |
|  | Adj_PCa5 | 3919 | 1005 | 3347 | 3918 | 1004 | 3338 |
|  | PCa5 | 2036 | 1178 | 4172 | 2034 | 1178 | 4136 |
|  | Adj_PCa6 | 3888 | 925 | 3190 | 3888 | 925 | 3190 |
|  | PCa6 | 2901 | 1188 | 4629 | 2899 | 1188 | 4608 |
|  | Adj_PCa7 | 737 | 1369 | 5898 | 735 | 1367 | 5793 |
|  | PCa7 | 1084 | 2711 | 12054 | 1067 | 2684 | 11313 |
|  | Adj_PCa8 | 8320 | 823 | 2408 | 8320 | 823 | 2408 |
|  | PCa8 | 3151 | 1000 | 2796 | 3150 | 1000 | 2787 |
|  | Adj_PCa9 | 1681 | 697 | 2122 | 1681 | 697 | 2122 |
|  | PCa9 | 4048 | 719 | 2147 | 4048 | 719 | 2147 |
|  | Adj_PCa10 | 5339 | 768 | 2589 | 5339 | 768 | 2589 |
|  | PCa10 | 6185 | 911 | 3376 | 6185 | 911 | 3376 |
|  | Adj_PCa11 | 4382 | 452 | 1371 | 4382 | 452 | 1371 |
|  | PCa11 | 4169 | 531 | 1730 | 4169 | 531 | 1730 |
|  | Adj_PCa12 | 7418 | 787 | 3004 | 7417 | 787 | 2998 |
|  | PCa12 | 4042 | 885 | 3359 | 4042 | 885 | 3359 |
|  | Adj_PCa13 | 7941 | 731 | 2537 | 7941 | 731 | 2537 |
|  | PCa13 | 6126 | 932 | 3055 | 6126 | 932 | 3055 |
|  | Adj_PCa14 | 5718 | 994 | 3820 | 5718 | 994 | 3820 |
|  | PCa14 | 8041 | 865 | 3419 | 8041 | 865 | 3419 |

Table S3. Marker genes for cell identification.

| cell Type | Major Marker Genes | References |
| --- | --- | --- |
| T cells | *CD3D*, CD247, CD3E, CD3G, CD7, CD8A, CD8B | [1] |
| Myeloid cells | *CD68*, CD163, C1QB, APOE, CSF1R, IF130, IL1B | [1, 3] |
| NK cells | *KLRF1*, FGFBP2, GNLY, KIR2DL4, CD160 | [1] |
| B cells | *CD22*, CD79A, CD79B, CD19, MS4A1 | [1] |
| Endothelial cells | *SELE*, VWF, IFI27, SPARCL1, SDPR, ACKR1, EGFL7 | [4] |
| LE cells | *KLK3*, KLK2, KLK4, ACPP, NKX3-1, DPP4, AR, KRT18 | [2] |
| BE cells | *KRT5*, KRT17, KRT14, KRT15, TP63, PDPN | [2] |
| Club cells | *WFDC2*, LCN2, MMP7, PIGR, CP | [2, 5] |
| Hillock cells | SCGB1A1, SOX9, TACSTD2, KRT13, S100A14, S100A16, KRT19, KRT4 | [2] |

Reference:

[1] Zheng, G. X. et al. Massively parallel digital transcriptional profiling of single cells. Nat Commun 8, 14049 (2017). DOI: 10.1038/ncomms14049

[2] Hirz T, Mei S, Sarkar H, et al. Dissecting the immune suppressive human prostate tumor microenvironment via integrated single-cell and spatial transcriptomic analyses. Nat Commun. 2023;14(1):663. doi:10.1038/s41467-023-36325-2

[3]Ma L, Hernandez MO, Zhao Y, et al. Tumor Cell Biodiversity Drives Microenvironmental Reprogramming in Liver Cancer. Cancer Cell. 2019;36(4):418-430.e6. doi:10.1016/j.ccell.2019.08.007

[4]Vanlandewijck M, He L, Mäe MA, et al. A molecular atlas of cell types and zonation in the brain vasculature. Nature. 2018;554(7693):475-480. doi:10.1038/nature25739

[5] Huang FW, Song H, Weinstein HN, et al. Club-like cells in proliferative inflammatory atrophy of the prostate. J Pathol. 2023;261(1):85-95. doi:10.1002/path.6149

Table S4. GEO datasets of included studies.

| GEO number | Sample_number | Sample name | Group |
| --- | --- | --- | --- |
| GSE172316 | GSM5252457 | NC1 | NC |
|  | GSM5252458 | NC2 | NC |
|  | GSM5252459 | NC3 | NC |
|  | GSM5252460 | NC4 | NC |
|  | GSM5252461 | NC5 | NC |
|  | GSM5252462 | NC6 | NC |
| GSE172301 | GSM5252126 | BPH1 | BPH |
|  | GSM5252127 | BPH2 | BPH |
|  | GSM5252128 | BPH3 | BPH |
|  | GSM5252129 | BPH4 | BPH |
|  | GSM5252130 | BPH5 | BPH |
|  | GSM5252131 | BPH6 | BPH |
|  | GSM5252132 | BPH7 | BPH |
|  | GSM5252133 | BPH8 | BPH |
|  | GSM5252134 | BPH9 | BPH |
|  | GSM5252135 | BPH10 | BPH |
|  | GSM5252136 | BPH11 | BPH |
|  | GSM5252137 | BPH12 | BPH |
|  | GSM5252138 | BPH13 | BPH |
| GSE181294 | GSM5494348 | PCa1 | PCa |
|  | GSM5494350 | PCa2 | PCa |
|  | GSM5494352 | PCa3 | PCa |
|  | GSM5494354 | PCa4 | PCa |
|  | GSM5494356 | PCa5 | PCa |
|  | GSM5494360 | PCa6 | PCa |
|  | GSM5494363 | PCa7 | PCa |
|  | GSM5494365 | PCa8 | PCa |
|  | GSM5494368 | PCa9 | PCa |
|  | GSM5494371 | PCa10 | PCa |
|  | GSM5494373 | PCa11 | PCa |
|  | GSM5494375 | PCa12 | PCa |
|  | GSM5494377 | PCa13 | PCa |
|  | GSM5494379 | PCa14 | PCa |
| GSE181294 | GSM5494347 | Adj-PCa1 | Adj-PCa |
|  | GSM5494351 | Adj-PCa2 | Adj-PCa |
|  | GSM5494353 | Adj-PCa3 | Adj-PCa |
|  | GSM5494355 | Adj-PCa4 | Adj-PCa |
|  | GSM5494357 | Adj-PCa5 | Adj-PCa |
|  | GSM5494361 | Adj-PCa6 | Adj-PCa |
|  | GSM5494364 | Adj-PCa7 | Adj-PCa |
|  | GSM5494366 | Adj-PCa8 | Adj-PCa |
|  | GSM5494367 | Adj-PCa9 | Adj-PCa |
|  | GSM5494370 | Adj-PCa10 | Adj-PCa |
|  | GSM5494372 | Adj-PCa11 | Adj-PCa |
|  | GSM5494374 | Adj-PCa12 | Adj-PCa |
|  | GSM5494376 | Adj-PCa13 | Adj-PCa |
|  | GSM5494378 | Adj-PCa14 | Adj-PCa |

Table S5. Marker genes for cell identification in datasets.

| cell Type | Major Marker Genes | References |
| --- | --- | --- |
| T cells | *CD3D,* CD247, CD3E, CD3G, CD7, CD8A, CD8B | [1] |
| Myeloid cells | *CD68,* CD163, C1QB, APOE, CSF1R, IF130, IL1B | [1, 3] |
| NK cells | *KLRF1,* FGFBP2, GNLY, KIR2DL4, CD160 | [1] |
| B cells | *CD22,* CD79A, CD79B, CD19, MS4A1 | [1] |
| Endothelial cells | *SELE,* VWF, IFI27, SPARCL1, SDPR, ACKR1, EGFL7 | [4] |
| LE cells | *KLK3,* KLK2, KLK4, ACPP, NKX3-1, DPP4, AR, KRT18 | [2] |
| BE cells | *KRT5,* KRT17, KRT14, KRT15, TP63 | [2] |
| Club cells | *WFDC2,* LCN2, MMP7, PIGR, CP | [2, 5] |
| Hillock cells | SCGB1A1, SOX9, TACSTD2, KRT13, S100A14, S100A16, KRT19, KRT4 | [2] |
| Fibroblasts | *DCN,* LUM, PDGFRA, COL1A1, COL3A1, MMP2 | [6, 7, 8] |
| SMC | PDGFRB, MCAM, MYH11, TAGLN, MYLK, TPM2 | [7, 9, 10] |
| Mast cells | MS4A2, CPA3, TPSAB1, KIT, TPSB2 | [10] |

Table S6. Genes used for evaluating Hyperplasia and Malignant states.

| Malignant | Hyerplasia |
| --- | --- |
| AMACR | ACPP |
| HOXC6 | AR |
| NKX3-1 | EGFR |
| PCA3 | KLK3 |
| SPINK1 | KRT18 |
| TMPRSS2 | KRT8 |
| LTF | NKX3-1 |
| AR | VEGFB |
| PIGR | THBS1 |
| SPON2 | SOX4 |
| ERG | SPDEF |
| FABP5 |  |
| GCNT1 |  |

Table S7. Shared and unique copy number variations (CNVs) between Hyperplasia and Malignant cells.

| Genes | CNV_type | Group |  |
| --- | --- | --- | --- |
| ABCF1, ABCF2, ABHD16A, ACAD8, ACOT13, AGAP3, AKAP9, ANKIB1, APLP2, ARHGAP32, ARHGEF12, ATP6V0E2, BAG6, BET1, C6orf47, C6orf62, CACNA2D1, CASD1, CDK5, CDK6, CFAP69, CFB, CLDN12, CLIC1, CROT, CSNK2B, CYP51A1, DCPS, DDAH2, DDR1, DDX39B, DHX16, DMTF1, DNAJB6, DPAGT1, DTX2, DXO, EHMT2, EI24, ERGIC2, ESYT2, ETS1, FAM133B, FASTK, FLOT1, FOXRED1, GALNT11, GATAD1, GIMAP1, GIMAP4, GIMAP7, GLB1L2, GMNN, GNAI1, GNG11, GNL1, GPANK1, GTF2I, GTF2IRD2B, GTPBP10, HCG18, HLA-B, HLA-C, HLA-E, HSPA1A, HSPA1B, HSPA8, HSPB1, IER3, INSIG1, KMT2C, KRIT1, LMBR1, LSM2, LTB, MDH2, MRPS18B, MRS2, NCAPD3, NELFE, NEU1, NFKBIL1, NUB1, PCLO, PDK4, PEX1, POM121C, PON2, POR, PPP1R10, PPP1R18, PPP1R9A, PRKAG2, PRRC2A, PTPRN2, RBM33, REPIN1, RHBDD2, RHEB, RING1, RPP21, RSBN1L, SC5D, SEMA3C, SIAE, SLC25A13, SLC25A40, SLC44A4, SNX19, SORL1, SOX4, SRI, SRPRA, ST14, STEAP1, STEAP2, STEAP4, STT3A, STYXL1, TBRG1, TDP2, THYN1, TMEM120A, TMEM218, TMEM243, TMEM60, TMUB1, TNF, TP53TG1, TRIM26, TRIM38, TUBB, UBE3C, VPS26B, VPS52, VSIG2, YWHAG, ZBTB44, ZNF862 | Amplification |  | **Shared CNVs** |
| APH1A, CCDC24, EPS8L1, MRPS21, OTUD7B, PPP6R1, PRPF3, VPS45 | Deletion |  |  |
| ECHDC2, SCP2, CPT2, MAGOH, YIPF1, LRRC42, BTN3A2, HMGN4, ABT1, TRIM27, UBD, HLA-F, HLA-A, PPP1R11, STK19, ATF6B, AGPAT1, MLLT10, GCHFR, DNAJC17, SPINT1, INO80, CHP1, RTF1, MGA, VPS39, TMEM87A, ZNF106, SNAP23, UBR1, CCNDBP1, TP53BP1, PDIA3, SERF2, DMAP1, ERI3, BOLA1, SF3B4, CISD3, SCAF1, IRF3, BCL2L12, MYADM, NDUFA3, TFPT, PRPF31, CNOT3, LENG1, TMC4, MBOAT7, TSEN34, RPS9, LENG8, LENG9, CDC42EP5 | Amplification | Hyperplasia | **Unique CNVs** |
| DMAP1, ERI3, BOLA1, SF3B4, CISD3, SCAF1, IRF3, BCL2L12, MYADM, NDUFA3, TFPT, PRPF31, CNOT3, LENG1, TMC4, MBOAT7, TSEN34, RPS9, LENG8, LENG9, CDC42EP5 | Deletion |  |  |
| HSD17B8, CLDN3, CLDN4, EIF4H, SDHAF3, LMTK2, TECPR1, RPS25, TRAPPC4, SLC37A4, HYOU1, DCXR, RFNG, GPS1, BRWD1, HMGN1, BACE2, FAM3B, TMPRSS2, C2CD2, TFF3, TFF1, SLC37A1 | Amplification | Malignant |  |
| RPRD2, SUCLG1, TMSB10, KCMF1, TGOLN2, RETSAT, CAPG, MAT2A, GGCX, POLR1D, POMP, SLC7A1, UBL3, HMGB1, ALOX5AP, HSPH1, N4BP2L2, PDS5B | Deletion |  |  |

Table S8. Marker genes used for cell type identification in RCTD analysis.

| All cell types | | Myeloid subtype | | LE cell states | |
| --- | --- | --- | --- | --- | --- |
| Gene | Cell type | Gene | Myeloid subtype | Gene | LE cell states |
| VPREB3 | B cell | APOC1 | Macrophages | RPS26 | Normal |
| CD79A | B cell | APOE | Macrophages | TRGC1 | Normal |
| FCRLA | B cell | SPP1 | Macrophages | LGALS3 | Normal |
| CD19 | B cell | TREM2 | Macrophages | CAB39L | Normal |
| JCHAIN | B cell | GPNMB | Macrophages | PRDX3 | Normal |
| IGHM | B cell | LIPA | Macrophages | GLO1 | Normal |
| TNFRSF13B | B cell | FABP5 | Macrophages | ANXA1 | Normal |
| IGKC | B cell | FCGR3A | Macrophages | ANXA3 | Normal |
| IGHA1 | B cell | LGMN | Macrophages | SRP9 | Normal |
| POU2AF1 | B cell | CD36 | Macrophages | PFN2 | Normal |
| IGHG1 | B cell | C5AR1 | Macrophages | NTAN1 | Normal |
| LINC00926 | B cell | GAL3ST4 | Macrophages | GNG4 | Normal |
| BLK | B cell | PMP22 | Macrophages | VBP1 | Normal |
| MS4A1 | B cell | PLD3 | Macrophages | CALM2 | Normal |
| TNFRSF13C | B cell | ACP5 | Macrophages | ARG2 | Normal |
| SPIB | B cell | SLC40A1 | Macrophages | HIBADH | Normal |
| HLA-DOB | B cell | DNASE2 | Macrophages | CCT8 | Normal |
| CD79B | B cell | CTSB | Macrophages | MYL12B | Normal |
| DERL3 | B cell | CD68 | Macrophages | CHMP5 | Normal |
| CD22 | B cell | C1QB | Macrophages | GLRX2 | Normal |
| AIM2 | B cell | CD14 | Macrophages | UBE2V1 | Normal |
| BANK1 | B cell | TNFSF13 | Macrophages | ACAT2 | Normal |
| ARHGAP24 | B cell | CREG1 | Macrophages | SMIM15 | Normal |
| HVCN1 | B cell | CD163 | Macrophages | CMBL | Normal |
| SELL | B cell | FCGRT | Macrophages | ADH5 | Normal |
| CCR7 | B cell | CFD | Macrophages | SCOC | Normal |
| SCIMP | B cell | LTA4H | Macrophages | CAPZA2 | Normal |
| CD37 | B cell | VCAN | Macrophages | ANXA4 | Normal |
| IRF8 | B cell | C1QC | Macrophages | MRPS35 | Normal |
| LTB | B cell | LY96 | Macrophages | COPZ1 | Normal |
| FCMR | B cell | VSIG4 | Macrophages | SKP1 | Normal |
| LYL1 | B cell | HBEGF | Macrophages | EIF3E | Normal |
| ADAM28 | B cell | SDS | Macrophages | UBE2V2 | Normal |
| RASGRP2 | B cell | FCGR1A | Macrophages | VDAC2 | Normal |
| PKIG | B cell | TMEM176A | Macrophages | EEF1G | Normal |
| PLCG2 | B cell | CD9 | Macrophages | PRDX1 | Normal |
| RALGPS2 | B cell | C1QA | Macrophages | LAMTOR3 | Normal |
| PARP15 | B cell | TMEM176B | Macrophages | SNX3 | Normal |
| TMEM154 | B cell | A2M | Macrophages | SEPHS2 | Normal |
| CD40 | B cell | TSPO | Macrophages | ARL3 | Normal |
| KLF2 | B cell | PECAM1 | Macrophages | NGRN | Normal |
| MEF2C | B cell | CD52 | Macrophages | MRPL17 | Normal |
| MARCH1 | B cell | OLR1 | Macrophages | AKR7A2 | Normal |
| POU2F2 | B cell | FTL | Macrophages | AP1S1 | Normal |
| BCAS4 | B cell | CTSL | Macrophages | S100A10 | Normal |
| SP140 | B cell | IFI6 | Macrophages | UBE2N | Normal |
| FCHSD2 | B cell | S100A4 | Macrophages | IMP3 | Normal |
| CD27 | B cell | MS4A4A | Macrophages | DSTN | Normal |
| BLNK | B cell | CRIP1 | Macrophages | STXBP6 | Normal |
| GNG7 | B cell | PLBD1 | Macrophages | BNIP3 | Normal |
| CXCR4 | B cell | MGST3 | Macrophages | POLR2K | Normal |
| CD83 | B cell | CTSD | Macrophages | SDHC | Normal |
| TCF4 | B cell | CRTAP | Macrophages | HADH | Normal |
| FCGR2B | B cell | CTSZ | Macrophages | ACY1 | Normal |
| CIITA | B cell | CTSS | Macrophages | DECR1 | Normal |
| PRKCB | B cell | LYZ | Macrophages | SNHG8 | Normal |
| FGD2 | B cell | CHMP1B | Macrophages | FKBP1A | Normal |
| TRAF5 | B cell | TYROBP | Macrophages | RSL24D1 | Normal |
| LY86 | B cell | MSR1 | Macrophages | EEF1A1 | Normal |
| SEL1L3 | B cell | FBP1 | Macrophages | SDHD | Normal |
| NCF1 | B cell | GLRX | Macrophages | PGAM1 | Normal |
| PIM2 | B cell | SULT1A1 | Macrophages | GABARAPL2 | Normal |
| LY9 | B cell | GM2A | Macrophages | APIP | Normal |
| HSH2D | B cell | S100A6 | Macrophages | LYPLA1 | Normal |
| NCF4 | B cell | HCST | Macrophages | MDH1 | Normal |
| PLEKHF2 | B cell | ASAH1 | Macrophages | CCDC115 | Normal |
| INPP5D | B cell | GLUL | Macrophages | RNF5 | Normal |
| LIMD2 | B cell | TIMP2 | Macrophages | ESD | Normal |
| SMAP2 | B cell | FPR3 | Macrophages | MYCBP | Normal |
| SYK | B cell | CD81 | Macrophages | BTF3L4 | Normal |
| CD55 | B cell | CTSA | Macrophages | TUBB | Normal |
| SH3BP5 | B cell | CSTA | Macrophages | NDRG3 | Normal |
| SWAP70 | B cell | ITM2B | Macrophages | RBM4 | Normal |
| ATP2A3 | B cell | NPC2 | Macrophages | DYNLT1 | Normal |
| SP110 | B cell | TNFSF13B | Macrophages | SH3BGRL | Normal |
| BIRC3 | B cell | CXCL16 | Macrophages | TOM1L1 | Normal |
| ANKRD44 | B cell | CD63 | Macrophages | RAB9A | Normal |
| TMEM243 | B cell | TGFBI | Macrophages | CSRP2 | Normal |
| RBM38 | B cell | CD37 | Macrophages | TADA3 | Normal |
| CD74 | B cell | CTSC | Macrophages | ETFA | Normal |
| RNASE6 | B cell | CHCHD10 | Macrophages | CKB | Normal |
| HLA-DQB1 | B cell | SLC7A7 | Macrophages | EIF4E | Normal |
| IL16 | B cell | PSAP | Macrophages | PSMA6 | Normal |
| LAT2 | B cell | S100A9 | Macrophages | AK6 | Normal |
| CD53 | B cell | C3AR1 | Macrophages | MRPS23 | Normal |
| IER5 | B cell | HNMT | Macrophages | QDPR | Normal |
| RCSD1 | B cell | TKT | Macrophages | SUMO1 | Normal |
| HLA-DMB | B cell | MARCH2 | Macrophages | SUCLG1 | Normal |
| RHOH | B cell | SCARB2 | Macrophages | NDUFA5 | Normal |
| ARHGAP25 | B cell | MNDA | Macrophages | MRPL13 | Normal |
| ISG20 | B cell | TNFSF12 | Macrophages | NDUFB3 | Normal |
| PTPN6 | B cell | BLVRA | Macrophages | MTHFD2 | Normal |
| HLA-DQA1 | B cell | LGALS3 | Macrophages | EIF2A | Normal |
| DRAM2 | B cell | ATP6V0C | Macrophages | C1D | Normal |
| CD69 | B cell | GIMAP4 | Macrophages | NUDT8 | Normal |
| FLI1 | B cell | KLF2 | Macrophages | PGK1 | Normal |
| EVI2B | B cell | SCPEP1 | Macrophages | RALB | Normal |
| TSPAN3 | B cell | MGST2 | Macrophages | COX7A2L | Normal |
| PMAIP1 | B cell | RAP2B | Macrophages | SULT2B1 | Normal |
| CD52 | B cell | BLVRB | Macrophages | VDAC1 | Normal |
| COL17A1 | Basal epithelial cell | HLA-DOB | DCs | PRR4 | Malignant |
| TP63 | Basal epithelial cell | CD1C | DCs | SPON2 | Malignant |
| DLK2 | Basal epithelial cell | CD1E | DCs | FABP5 | Malignant |
| TNS4 | Basal epithelial cell | CD207 | DCs | CD177 | Malignant |
| FLRT3 | Basal epithelial cell | PPP1R14A | DCs | TFF3 | Malignant |
| KRT5 | Basal epithelial cell | ASB2 | DCs | ACP5 | Malignant |
| KRT14 | Basal epithelial cell | CCR6 | DCs | NPY | Malignant |
| FBLN1 | Basal epithelial cell | CDH17 | DCs | APP | Malignant |
| KRT15 | Basal epithelial cell | FLT3 | DCs | IFI6 | Malignant |
| TRIM29 | Basal epithelial cell | FCER1A | DCs | ABCC4 | Malignant |
| KRT17 | Basal epithelial cell | LTB | DCs | SERPINA3 | Malignant |
| SLC14A1 | Basal epithelial cell | HPGDS | DCs | PSCA | Malignant |
| SFRP1 | Basal epithelial cell | PARM1 | DCs | SLC4A4 | Malignant |
| GJA1 | Basal epithelial cell | AFF3 | DCs | PTPRM | Malignant |
| PDPN | Basal epithelial cell | CLEC10A | DCs | WFDC2 | Malignant |
| SERPINF1 | Basal epithelial cell | MCOLN2 | DCs | CLGN | Malignant |
| CYP4B1 | Basal epithelial cell | SLC38A1 | DCs | CACNA2D1 | Malignant |
| TIMP3 | Basal epithelial cell | PIK3R6 | DCs | GGT1 | Malignant |
| COL7A1 | Basal epithelial cell | HLA-DQB2 | DCs | IGFBP5 | Malignant |
| MEG3 | Basal epithelial cell | ADGRE3 | DCs | PODXL2 | Malignant |
| NTN4 | Basal epithelial cell | DUSP5 | DCs | GLB1L2 | Malignant |
| F3 | Basal epithelial cell | ADAM19 | DCs | LY6E | Malignant |
| LAMB3 | Basal epithelial cell | CACNA2D3 | DCs | STEAP4 | Malignant |
| FGFR2 | Basal epithelial cell | CD69 | DCs | FADS2 | Malignant |
| EFEMP1 | Basal epithelial cell | JAML | DCs | ANPEP | Malignant |
| DSC3 | Basal epithelial cell | AREG | DCs | SERPING1 | Malignant |
| ITGB4 | Basal epithelial cell | TLR10 | DCs | NUCB2 | Malignant |
| PLS3 | Basal epithelial cell | FAM117A | DCs | ABCC1 | Malignant |
| MACROD2 | Basal epithelial cell | TNFSF9 | DCs | NBPF14 | Malignant |
| CAV2 | Basal epithelial cell | CST7 | DCs | LRPAP1 | Malignant |
| FERMT1 | Basal epithelial cell | CD1D | DCs | DLGAP1 | Malignant |
| S100A2 | Basal epithelial cell | PKIB | DCs | MME | Malignant |
| FAM107A | Basal epithelial cell | MS4A4E | DCs | MFSD12 | Malignant |
| DKK3 | Basal epithelial cell | PLAC8 | DCs | SEC11C | Malignant |
| PITX1 | Basal epithelial cell | PLD4 | DCs | TMEM63A | Malignant |
| SOX15 | Basal epithelial cell | RHOF | DCs | GUSB | Malignant |
| EFEMP2 | Basal epithelial cell | DAPP1 | DCs | ARSA | Malignant |
| TNS1 | Basal epithelial cell | GPAT3 | DCs | HPN | Malignant |
| DST | Basal epithelial cell | PON2 | DCs | CLDN7 | Malignant |
| IGFBP7 | Basal epithelial cell | CEACAM4 | DCs | HIST2H2AA3 | Malignant |
| MEIS2 | Basal epithelial cell | ENPP2 | DCs | TM4SF1 | Malignant |
| IGFBP4 | Basal epithelial cell | GEM | DCs | TRPM4 | Malignant |
| COL4A5 | Basal epithelial cell | HLA-DQB1 | DCs | MDK | Malignant |
| CAV1 | Basal epithelial cell | PRKAR2B | DCs | TMEM175 | Malignant |
| CLDN1 | Basal epithelial cell | HIC1 | DCs | CHPF | Malignant |
| CCL2 | Basal epithelial cell | ITGB7 | DCs | RIC3 | Malignant |
| GAS6 | Basal epithelial cell | KCNMB1 | DCs | NPNT | Malignant |
| DEFB1 | Basal epithelial cell | GRASP | DCs | ITGA6 | Malignant |
| MARVELD1 | Basal epithelial cell | PPA1 | DCs | SEC61A1 | Malignant |
| FHL2 | Basal epithelial cell | SEL1L3 | DCs | MT-ATP6 | Malignant |
| SDC1 | Basal epithelial cell | HLA-DQA1 | DCs | SLC12A2 | Malignant |
| TMEM237 | Basal epithelial cell | BTN2A2 | DCs | NAAA | Malignant |
| ITGA2 | Basal epithelial cell | SULF2 | DCs | LRIG1 | Malignant |
| MYL9 | Basal epithelial cell | MAP4K1 | DCs | ECE1 | Malignant |
| USP31 | Basal epithelial cell | DENND1B | DCs | HLA-B | Malignant |
| SCUBE2 | Basal epithelial cell | CXorf21 | DCs | ABHD2 | Malignant |
| PDK4 | Basal epithelial cell | ATP2A3 | DCs | SLC26A2 | Malignant |
| ITGB8 | Basal epithelial cell | NDRG2 | DCs | CD151 | Malignant |
| MPZL2 | Basal epithelial cell | FCGR2B | DCs | PLEC | Malignant |
| HOXD11 | Basal epithelial cell | HDAC9 | DCs | DEGS1 | Malignant |
| SMARCD3 | Basal epithelial cell | SEMA4C | DCs | SPTBN2 | Malignant |
| PDLIM1 | Basal epithelial cell | CYSLTR1 | DCs | AGTRAP | Malignant |
| CLU | Basal epithelial cell | NRARP | DCs | BMPR1B | Malignant |
| ISYNA1 | Basal epithelial cell | IL2RG | DCs | UPK3A | Malignant |
| MCC | Basal epithelial cell | CYP2S1 | DCs | TMEM8A | Malignant |
| SVIL | Basal epithelial cell | PRCP | DCs | ST14 | Malignant |
| CDCA7L | Basal epithelial cell | SPN | DCs | KLK11 | Malignant |
| ST5 | Basal epithelial cell | P2RY13 | DCs | FAM118A | Malignant |
| PTPN14 | Basal epithelial cell | F11R | DCs | MARC1 | Malignant |
| EGFR | Basal epithelial cell | RAB24 | DCs | GRN | Malignant |
| JAG1 | Basal epithelial cell | MOB3B | DCs | IGF1R | Malignant |
| PALLD | Basal epithelial cell | ACAP1 | DCs | PPIB | Malignant |
| TPM2 | Basal epithelial cell | SPINT1 | DCs | MYDGF | Malignant |
| EYA2 | Basal epithelial cell | GAPT | DCs | SLC38A10 | Malignant |
| ASPH | Basal epithelial cell | FAM89B | DCs | ANG | Malignant |
| THSD4 | Basal epithelial cell | SPINT2 | DCs | ZBTB16 | Malignant |
| ETS2 | Basal epithelial cell | ATP6V0A2 | DCs | SIL1 | Malignant |
| COLCA1 | Basal epithelial cell | CD38 | DCs | CD320 | Malignant |
| PERP | Basal epithelial cell | OPN3 | DCs | LIFR | Malignant |
| ITGA6 | Basal epithelial cell | REPIN1 | DCs | CRELD2 | Malignant |
| IRX3 | Basal epithelial cell | KCNK6 | DCs | ZNF827 | Malignant |
| TRIP6 | Basal epithelial cell | FILIP1L | DCs | FOS | Malignant |
| KCTD1 | Basal epithelial cell | GPR183 | DCs | MTDH | Malignant |
| CRIM1 | Basal epithelial cell | HLA-DRA | DCs | CALR | Malignant |
| PHLDA3 | Basal epithelial cell | MTR | DCs | GOLM1 | Malignant |
| IGFBP2 | Basal epithelial cell | PALLD | DCs | HLA-A | Malignant |
| C1S | Basal epithelial cell | FAM118A | DCs | CTSF | Malignant |
| KANK1 | Basal epithelial cell | HLA-DOA | DCs | LSAMP | Malignant |
| LAPTM4B | Basal epithelial cell | HLA-DPB1 | DCs | BCAM | Malignant |
| KRT19 | Basal epithelial cell | C12orf45 | DCs | SLC22A17 | Malignant |
| NDN | Basal epithelial cell | ABCC1 | DCs | AZGP1 | Malignant |
| DMKN | Basal epithelial cell | STK17A | DCs | DDRGK1 | Malignant |
| RGCC | Basal epithelial cell | ITGB2-AS1 | DCs | ACP2 | Malignant |
| TSKU | Basal epithelial cell | HLA-DRB5 | DCs | COL6A2 | Malignant |
| KRT7 | Basal epithelial cell | ADAM8 | DCs | AGRN | Malignant |
| TFAP2A | Basal epithelial cell | HLA-DRB1 | DCs | PRDX4 | Malignant |
| WLS | Basal epithelial cell | UVRAG | DCs | ASTN2 | Malignant |
| PRSS23 | Basal epithelial cell | HLA-DPA1 | DCs | ZDHHC24 | Malignant |
| CITED4 | Basal epithelial cell | ICAM3 | DCs | RTN1 | Malignant |
| RASSF6 | Basal epithelial cell | MYCL | DCs | PARM1 | Malignant |
| MMP7 | Club cell | MT1A | Monocytes | LCN2 | Adj_Hyperplasia |
| SAA2 | Club cell | MT1M | Monocytes | LRG1 | Adj_Hyperplasia |
| SAA1 | Club cell | MT1G | Monocytes | THBS1 | Adj_Hyperplasia |
| PIGR | Club cell | MT2A | Monocytes | SLC26A4 | Adj_Hyperplasia |
| CP | Club cell | MT1X | Monocytes | NNMT | Adj_Hyperplasia |
| CHI3L2 | Club cell | MT1H | Monocytes | EMP1 | Adj_Hyperplasia |
| RARRES1 | Club cell | MT1F | Monocytes | IER3 | Adj_Hyperplasia |
| CXCL17 | Club cell | MT1E | Monocytes | CFB | Adj_Hyperplasia |
| CX3CL1 | Club cell | HIST1H4C | Monocytes | CEACAM1 | Adj_Hyperplasia |
| LCN2 | Club cell | HMGB2 | Monocytes | PTP4A3 | Adj_Hyperplasia |
| LTF | Club cell | FKBP4 | Monocytes | THSD4 | Adj_Hyperplasia |
| C1S | Club cell | KLK3 | Monocytes | SERPINA1 | Adj_Hyperplasia |
| FAM3D | Club cell | CXCL8 | Monocytes | SOD2 | Adj_Hyperplasia |
| MGP | Club cell | TNFAIP2 | Monocytes | PDZK1IP1 | Adj_Hyperplasia |
| GPRC5A | Club cell | MSMB | Monocytes | RUNX1 | Adj_Hyperplasia |
| KRT7 | Club cell | SOD2 | Monocytes | IL4R | Adj_Hyperplasia |
| SCUBE2 | Club cell | C15orf48 | Monocytes | TMC5 | Adj_Hyperplasia |
| WFDC2 | Club cell | MALAT1 | Monocytes | PLA2G2A | Adj_Hyperplasia |
| PDZK1IP1 | Club cell | WTAP | Monocytes | SBNO2 | Adj_Hyperplasia |
| SLPI | Club cell | ACSL1 | Monocytes | ITPKC | Adj_Hyperplasia |
| RHOV | Club cell | SLC25A37 | Monocytes | CYBA | Adj_Hyperplasia |
| SLC26A4 | Club cell | CCL4 | Monocytes | NEAT1 | Adj_Hyperplasia |
| CFB | Club cell | TNIP1 | Monocytes | SLC26A2 | Adj_Hyperplasia |
| C1R | Club cell | ACPP | Monocytes | MT1X | Adj_Hyperplasia |
| MUC4 | Club cell | SUPT5H | Monocytes | CD74 | Adj_Hyperplasia |
| CRABP2 | Club cell | HIST1H1E | Monocytes | RAB17 | Adj_Hyperplasia |
| MUC1 | Club cell | LUC7L3 | Monocytes | VMP1 | Adj_Hyperplasia |
| FUT2 | Club cell | ARGLU1 | Monocytes | GPX3 | Adj_Hyperplasia |
| UBD | Club cell | MT-ATP6 | Monocytes | MAST4 | Adj_Hyperplasia |
| SERPINA3 | Club cell | MT-ND4L | Monocytes | RNF145 | Adj_Hyperplasia |
| MUC20 | Club cell | SPAG9 | Monocytes | PLIN5 | Adj_Hyperplasia |
| LXN | Club cell | TNFAIP3 | Monocytes | ZFP36 | Adj_Hyperplasia |
| RASL11A | Club cell | FKBP5 | Monocytes | SLC11A2 | Adj_Hyperplasia |
| C4A | Club cell | MT-ND5 | Monocytes | IFITM2 | Adj_Hyperplasia |
| TNFRSF12A | Club cell | NEAT1 | Monocytes | LGMN | Adj_Hyperplasia |
| C3 | Club cell | CCL3 | Monocytes | SERPING1 | Adj_Hyperplasia |
| NCOA7 | Club cell | KYNU | Monocytes | DNAH5 | Adj_Hyperplasia |
| AGR2 | Club cell | MT-CO1 | Monocytes | HLA-DRA | Adj_Hyperplasia |
| ROBO1 | Club cell | ITGA5 | Monocytes | CTSB | Adj_Hyperplasia |
| ATP1B1 | Club cell | MT-ATP8 | Monocytes | IL13RA1 | Adj_Hyperplasia |
| ASS1 | Club cell | MT-ND1 | Monocytes | SEMA4B | Adj_Hyperplasia |
| SLC5A1 | Club cell | CCL4L2 | Monocytes | RALGDS | Adj_Hyperplasia |
| PLEKHS1 | Club cell | MT-CYB | Monocytes | PPP1R15A | Adj_Hyperplasia |
| TM4SF1 | Club cell | HIST1H1C | Monocytes | CEBPD | Adj_Hyperplasia |
| CLU | Club cell | MT-CO2 | Monocytes | ARRDC3 | Adj_Hyperplasia |
| GSTA1 | Club cell | MT-ND6 | Monocytes | ATP6V0A1 | Adj_Hyperplasia |
| KRT19 | Club cell | MT-ND2 | Monocytes | HLA-DRB1 | Adj_Hyperplasia |
| TNFAIP2 | Club cell | TUBB4B | Monocytes | RPRD1B | Adj_Hyperplasia |
| TNFRSF21 | Club cell | MT-ND4 | Monocytes | ATF3 | Adj_Hyperplasia |
| CFTR | Club cell | DSTN | Monocytes | WBP1L | Adj_Hyperplasia |
| PLPP2 | Club cell | MARCKSL1 | Monocytes | SCNN1A | Adj_Hyperplasia |
| CDH3 | Club cell | NFKBIZ | Monocytes | TGM2 | Adj_Hyperplasia |
| PTGES | Club cell | FNDC3B | Monocytes | ATP1A1 | Adj_Hyperplasia |
| S100P | Club cell | MT-CO3 | Monocytes | MT1M | Adj_Hyperplasia |
| CXCL2 | Club cell | HP1BP3 | Monocytes | MT1G | Adj_Hyperplasia |
| NNMT | Club cell | HMGA1 | Monocytes | ZDHHC9 | Adj_Hyperplasia |
| PTPRK | Club cell | IL1B | Monocytes | CXADR | Adj_Hyperplasia |
| SERPING1 | Club cell | G0S2 | Monocytes | SIM2 | Adj_Hyperplasia |
| ELF3 | Club cell | TRIM28 | Monocytes | ETV6 | Adj_Hyperplasia |
| FHL2 | Club cell | XBP1 | Monocytes | SLC9A9 | Adj_Hyperplasia |
| SOD2 | Club cell | MT-ND3 | Monocytes | MT2A | Adj_Hyperplasia |
| ST5 | Club cell | PDLIM5 | Monocytes | PDE4B | Adj_Hyperplasia |
| STEAP3 | Club cell | H1F0 | Monocytes | BCL3 | Adj_Hyperplasia |
| DNAJC12 | Club cell | SRRM2 | Monocytes | IGFBP5 | Adj_Hyperplasia |
| SDC4 | Club cell | GLS | Monocytes | GPAT4 | Adj_Hyperplasia |
| PRSS22 | Club cell | TUBA1B | Monocytes | TACSTD2 | Adj_Hyperplasia |
| KCNQ1 | Club cell | C1orf122 | Monocytes | TIMP1 | Adj_Hyperplasia |
| CEACAM1 | Club cell | FUS | Monocytes | IFNGR1 | Adj_Hyperplasia |
| EMP1 | Club cell | CELF2 | Monocytes | TCIRG1 | Adj_Hyperplasia |
| CDC42EP1 | Club cell | H1FX | Monocytes | NFKBIZ | Adj_Hyperplasia |
| OCLN | Club cell | DOCK4 | Monocytes | SYNE2 | Adj_Hyperplasia |
| CLDN4 | Club cell | SLC8A1 | Monocytes | TRIM36 | Adj_Hyperplasia |
| ITGB8 | Club cell | SQSTM1 | Monocytes | SHB | Adj_Hyperplasia |
| ITGA3 | Club cell | TMEM259 | Monocytes | RALGAPA2 | Adj_Hyperplasia |
| OSBPL10 | Club cell | ADGRE2 | Monocytes | KLF6 | Adj_Hyperplasia |
| PLA2G2A | Club cell | EHBP1L1 | Monocytes | NAMPT | Adj_Hyperplasia |
| RBPMS | Club cell | GPRIN3 | Monocytes | CTSL | Adj_Hyperplasia |
| LRG1 | Club cell | PPDPF | Monocytes | MIDN | Adj_Hyperplasia |
| PPIC | Club cell | BCL2A1 | Monocytes | BACE2 | Adj_Hyperplasia |
| MDK | Club cell | NAMPT | Monocytes | TSPYL2 | Adj_Hyperplasia |
| MYH14 | Club cell | IER3 | Monocytes | FURIN | Adj_Hyperplasia |
| CXADR | Club cell | SON | Monocytes | LDLR | Adj_Hyperplasia |
| OSMR | Club cell | HMGB1 | Monocytes | FOSB | Adj_Hyperplasia |
| SCNN1A | Club cell | NFKBIA | Monocytes | LRP10 | Adj_Hyperplasia |
| BIRC3 | Club cell | ITPR2 | Monocytes | THADA | Adj_Hyperplasia |
| CD59 | Club cell | PRPF38B | Monocytes | PRSS22 | Adj_Hyperplasia |
| TGM2 | Club cell | JMJD1C | Monocytes | VSTM2L | Adj_Hyperplasia |
| INSR | Club cell | IL17RA | Monocytes | PIEZO1 | Adj_Hyperplasia |
| BACE2 | Club cell | ADPGK | Monocytes | TAPBP | Adj_Hyperplasia |
| GALNT18 | Club cell | BPTF | Monocytes | VWA1 | Adj_Hyperplasia |
| MAST4 | Club cell | ITSN2 | Monocytes | SGSM2 | Adj_Hyperplasia |
| SELENBP1 | Club cell | DAPK1 | Monocytes | LRIG1 | Adj_Hyperplasia |
| TACSTD2 | Club cell | EIF4A3 | Monocytes | SLC44A4 | Adj_Hyperplasia |
| CLDN7 | Club cell | STAB1 | Monocytes | CPLX3 | Adj_Hyperplasia |
| ITPKC | Club cell | ARHGAP4 | Monocytes | KCNK6 | Adj_Hyperplasia |
| AGRN | Club cell | MYCBP2 | Monocytes | INSR | Adj_Hyperplasia |
| DDR1 | Club cell | CHD9 | Monocytes | CD44 | Adj_Hyperplasia |
| EVA1C | Club cell | EPC1 | Monocytes | DNAJB1 | Adj_Hyperplasia |
| DUSP23 | Club cell | HSP90AA1 | Monocytes | ENTPD6 | Adj_Hyperplasia |
| THSD4 | Club cell | TRIM44 | Monocytes | ASRGL1 | Adj_Hyperplasia |
| FAM110D | Endothelial cell |  |  | LINC01297 | Hyperplasia |
| ACKR1 | Endothelial cell |  |  | POTEH | Hyperplasia |
| SOX18 | Endothelial cell |  |  | GP2 | Hyperplasia |
| CYYR1 | Endothelial cell |  |  | MT-ATP8 | Hyperplasia |
| CCL14 | Endothelial cell |  |  | CWH43 | Hyperplasia |
| MEOX1 | Endothelial cell |  |  | PI15 | Hyperplasia |
| NRN1 | Endothelial cell |  |  | SLC22A3 | Hyperplasia |
| RBP5 | Endothelial cell |  |  | ELK4 | Hyperplasia |
| FAM167B | Endothelial cell |  |  | LMAN1L | Hyperplasia |
| IFI27 | Endothelial cell |  |  | MT-ND3 | Hyperplasia |
| LDB2 | Endothelial cell |  |  | SLC5A3 | Hyperplasia |
| LIMS2 | Endothelial cell |  |  | SLC16A1 | Hyperplasia |
| IL33 | Endothelial cell |  |  | LGALS3BP | Hyperplasia |
| GNG11 | Endothelial cell |  |  | SCD | Hyperplasia |
| SPARCL1 | Endothelial cell |  |  | PDIA4 | Hyperplasia |
| EGFL7 | Endothelial cell |  |  | DHRS7 | Hyperplasia |
| SNCG | Endothelial cell |  |  | SEMA6A | Hyperplasia |
| LMCD1 | Endothelial cell |  |  | SEZ6L2 | Hyperplasia |
| CAV1 | Endothelial cell |  |  | COLEC12 | Hyperplasia |
| IGFBP4 | Endothelial cell |  |  | SLC38A2 | Hyperplasia |
| LMO2 | Endothelial cell |  |  | CPLX3 | Hyperplasia |
| IGFBP7 | Endothelial cell |  |  | HSPA5 | Hyperplasia |
| NOSTRIN | Endothelial cell |  |  | HSP90B1 | Hyperplasia |
| SPRY1 | Endothelial cell |  |  | HLA-A | Hyperplasia |
| CRIP2 | Endothelial cell |  |  | RDH11 | Hyperplasia |
| ID3 | Endothelial cell |  |  | PCSK1N | Hyperplasia |
| RNASE1 | Endothelial cell |  |  | LAMB2 | Hyperplasia |
| PDLIM1 | Endothelial cell |  |  | ACPP | Hyperplasia |
| VAMP5 | Endothelial cell |  |  | FADS2 | Hyperplasia |
| EHD4 | Endothelial cell |  |  | PEBP4 | Hyperplasia |
| ID1 | Endothelial cell |  |  | ATP1B3 | Hyperplasia |
| CTNNAL1 | Endothelial cell |  |  | MYO1E | Hyperplasia |
| SH3BP5 | Endothelial cell |  |  | PDIA6 | Hyperplasia |
| CCDC85B | Endothelial cell |  |  | HYOU1 | Hyperplasia |
| AP1S2 | Endothelial cell |  |  | SEL1L | Hyperplasia |
| ETS2 | Endothelial cell |  |  | HLA-C | Hyperplasia |
| S100A16 | Endothelial cell |  |  | SLC22A17 | Hyperplasia |
| NR2F2 | Endothelial cell |  |  | MAN2A1 | Hyperplasia |
| KLF2 | Endothelial cell |  |  | LAMP2 | Hyperplasia |
| TCF4 | Endothelial cell |  |  | SIAE | Hyperplasia |
| CNN3 | Endothelial cell |  |  | CTSF | Hyperplasia |
| ARL4A | Endothelial cell |  |  | SQLE | Hyperplasia |
| SOCS3 | Endothelial cell |  |  | MANBA | Hyperplasia |
| DUSP23 | Endothelial cell |  |  | KIAA1324 | Hyperplasia |
| S100A13 | Endothelial cell |  |  | ERO1B | Hyperplasia |
| GRASP | Endothelial cell |  |  | ATP2C1 | Hyperplasia |
| TPD52L1 | Endothelial cell |  |  | ZDHHC14 | Hyperplasia |
| EPAS1 | Endothelial cell |  |  | FOLH1 | Hyperplasia |
| CLIC4 | Endothelial cell |  |  | APOL1 | Hyperplasia |
| GIMAP1 | Endothelial cell |  |  | SLC35F2 | Hyperplasia |
| VIM | Endothelial cell |  |  | METTL7A | Hyperplasia |
| GIMAP7 | Endothelial cell |  |  | HIST1H2BG | Hyperplasia |
| SDCBP | Endothelial cell |  |  | MALAT1 | Hyperplasia |
| FKBP1A | Endothelial cell |  |  | PDIA3 | Hyperplasia |
| IFITM3 | Endothelial cell |  |  | PLOD1 | Hyperplasia |
| GIMAP4 | Endothelial cell |  |  | FSTL1 | Hyperplasia |
| IFITM2 | Endothelial cell |  |  | TAP1 | Hyperplasia |
| ARL2 | Endothelial cell |  |  | MXRA7 | Hyperplasia |
| VAMP3 | Endothelial cell |  |  | MIA3 | Hyperplasia |
| BCL3 | Endothelial cell |  |  | MT-ND4L | Hyperplasia |
| SNHG7 | Endothelial cell |  |  | PBXIP1 | Hyperplasia |
| TGFBR2 | Endothelial cell |  |  | BTN3A2 | Hyperplasia |
| NNMT | Endothelial cell |  |  | ATP8A1 | Hyperplasia |
| PIM3 | Endothelial cell |  |  | NEU1 | Hyperplasia |
| POLE4 | Endothelial cell |  |  | HLA-B | Hyperplasia |
| S100A6 | Endothelial cell |  |  | UGGT1 | Hyperplasia |
| CRTAP | Endothelial cell |  |  | ATP8B1 | Hyperplasia |
| TAGLN2 | Endothelial cell |  |  | ASTN2 | Hyperplasia |
| CARHSP1 | Endothelial cell |  |  | GGT1 | Hyperplasia |
| PLSCR1 | Endothelial cell |  |  | ATF3 | Hyperplasia |
| IFITM1 | Endothelial cell |  |  | PRSS8 | Hyperplasia |
| SNX3 | Endothelial cell |  |  | KLK3 | Hyperplasia |
| RALB | Endothelial cell |  |  | ITGB1 | Hyperplasia |
| RAB11A | Endothelial cell |  |  | SOAT1 | Hyperplasia |
| GUK1 | Endothelial cell |  |  | DSG2 | Hyperplasia |
| HEBP1 | Endothelial cell |  |  | MT-ND2 | Hyperplasia |
| BHLHE40 | Endothelial cell |  |  | GAA | Hyperplasia |
| RAB13 | Endothelial cell |  |  | SLC9A2 | Hyperplasia |
| TSC22D1 | Endothelial cell |  |  | CD82 | Hyperplasia |
| PEA15 | Endothelial cell |  |  | INHBB | Hyperplasia |
| BST2 | Endothelial cell |  |  | GGCX | Hyperplasia |
| PCBP2 | Endothelial cell |  |  | PCYOX1 | Hyperplasia |
| TACC1 | Endothelial cell |  |  | ANTXR2 | Hyperplasia |
| SLC9A3R2 | Endothelial cell |  |  | DNASE2 | Hyperplasia |
| LDHA | Endothelial cell |  |  | DNAJC3 | Hyperplasia |
| CDK2AP1 | Endothelial cell |  |  | ZDHHC20 | Hyperplasia |
| LAP3 | Endothelial cell |  |  | POTEH-AS1 | Hyperplasia |
| YBX3 | Endothelial cell |  |  | OLMALINC | Hyperplasia |
| MYL12A | Endothelial cell |  |  | FOSB | Hyperplasia |
| RHOC | Endothelial cell |  |  | ENPP5 | Hyperplasia |
| TMSB10 | Endothelial cell |  |  | PLEKHH1 | Hyperplasia |
| CDC37 | Endothelial cell |  |  | KIAA1109 | Hyperplasia |
| RPS23 | Endothelial cell |  |  | COL6A2 | Hyperplasia |
| HSPB1 | Endothelial cell |  |  | FMOD | Hyperplasia |
| ANXA2 | Endothelial cell |  |  | SLC15A2 | Hyperplasia |
| ARPC1B | Endothelial cell |  |  | OS9 | Hyperplasia |
| BTF3 | Endothelial cell |  |  | IGF2R | Hyperplasia |
| GMFG | Endothelial cell |  |  | APLP2 | Hyperplasia |
| FLOT1 | Endothelial cell |  |  | KIF5C | Hyperplasia |
| C4orf3 | Endothelial cell |  |  | ACSL3 | Hyperplasia |
| TFF3 | Luminal epithelial cell |  |  |  |  |
| TMEFF2 | Luminal epithelial cell |  |  |  |  |
| OR51E2 | Luminal epithelial cell |  |  |  |  |
| SLC45A3 | Luminal epithelial cell |  |  |  |  |
| KLK3 | Luminal epithelial cell |  |  |  |  |
| KLK2 | Luminal epithelial cell |  |  |  |  |
| LMAN1L | Luminal epithelial cell |  |  |  |  |
| NPY | Luminal epithelial cell |  |  |  |  |
| RLN1 | Luminal epithelial cell |  |  |  |  |
| NEFH | Luminal epithelial cell |  |  |  |  |
| TRPV6 | Luminal epithelial cell |  |  |  |  |
| TRGC1 | Luminal epithelial cell |  |  |  |  |
| KLK4 | Luminal epithelial cell |  |  |  |  |
| MESP1 | Luminal epithelial cell |  |  |  |  |
| ACPP | Luminal epithelial cell |  |  |  |  |
| FOLH1 | Luminal epithelial cell |  |  |  |  |
| ANO7 | Luminal epithelial cell |  |  |  |  |
| MSMB | Luminal epithelial cell |  |  |  |  |
| LRRC26 | Luminal epithelial cell |  |  |  |  |
| CPNE4 | Luminal epithelial cell |  |  |  |  |
| GNG4 | Luminal epithelial cell |  |  |  |  |
| GREB1 | Luminal epithelial cell |  |  |  |  |
| ARG2 | Luminal epithelial cell |  |  |  |  |
| HGD | Luminal epithelial cell |  |  |  |  |
| SPDEF | Luminal epithelial cell |  |  |  |  |
| CNTNAP2 | Luminal epithelial cell |  |  |  |  |
| VGLL3 | Luminal epithelial cell |  |  |  |  |
| HMGCS2 | Luminal epithelial cell |  |  |  |  |
| RAB3B | Luminal epithelial cell |  |  |  |  |
| MYBPC1 | Luminal epithelial cell |  |  |  |  |
| NKX3-1 | Luminal epithelial cell |  |  |  |  |
| GNMT | Luminal epithelial cell |  |  |  |  |
| TMSB15A | Luminal epithelial cell |  |  |  |  |
| COBL | Luminal epithelial cell |  |  |  |  |
| CKB | Luminal epithelial cell |  |  |  |  |
| SORD | Luminal epithelial cell |  |  |  |  |
| TMPRSS2 | Luminal epithelial cell |  |  |  |  |
| RAMP1 | Luminal epithelial cell |  |  |  |  |
| TRPM8 | Luminal epithelial cell |  |  |  |  |
| SYT7 | Luminal epithelial cell |  |  |  |  |
| C1orf116 | Luminal epithelial cell |  |  |  |  |
| PDE11A | Luminal epithelial cell |  |  |  |  |
| ALOX15B | Luminal epithelial cell |  |  |  |  |
| SLC30A4 | Luminal epithelial cell |  |  |  |  |
| BCAS1 | Luminal epithelial cell |  |  |  |  |
| HOXA11-AS | Luminal epithelial cell |  |  |  |  |
| PART1 | Luminal epithelial cell |  |  |  |  |
| SULT2B1 | Luminal epithelial cell |  |  |  |  |
| STEAP2 | Luminal epithelial cell |  |  |  |  |
| SPOCK1 | Luminal epithelial cell |  |  |  |  |
| SLC2A12 | Luminal epithelial cell |  |  |  |  |
| HOXB13 | Luminal epithelial cell |  |  |  |  |
| C9orf152 | Luminal epithelial cell |  |  |  |  |
| RDH11 | Luminal epithelial cell |  |  |  |  |
| HPN | Luminal epithelial cell |  |  |  |  |
| PYCR1 | Luminal epithelial cell |  |  |  |  |
| TTC39A | Luminal epithelial cell |  |  |  |  |
| PDE9A | Luminal epithelial cell |  |  |  |  |
| ALDH1A3 | Luminal epithelial cell |  |  |  |  |
| CDC42EP5 | Luminal epithelial cell |  |  |  |  |
| AR | Luminal epithelial cell |  |  |  |  |
| FAM3B | Luminal epithelial cell |  |  |  |  |
| ZNF761 | Luminal epithelial cell |  |  |  |  |
| CREB3L4 | Luminal epithelial cell |  |  |  |  |
| CAB39L | Luminal epithelial cell |  |  |  |  |
| ADIRF | Luminal epithelial cell |  |  |  |  |
| SEC14L2 | Luminal epithelial cell |  |  |  |  |
| RWDD2A | Luminal epithelial cell |  |  |  |  |
| PMEPA1 | Luminal epithelial cell |  |  |  |  |
| BMPR1B | Luminal epithelial cell |  |  |  |  |
| TMEM125 | Luminal epithelial cell |  |  |  |  |
| HOXA10 | Luminal epithelial cell |  |  |  |  |
| ARFGEF3 | Luminal epithelial cell |  |  |  |  |
| NUDT8 | Luminal epithelial cell |  |  |  |  |
| CRYM | Luminal epithelial cell |  |  |  |  |
| CPE | Luminal epithelial cell |  |  |  |  |
| TCEA3 | Luminal epithelial cell |  |  |  |  |
| NCAPD3 | Luminal epithelial cell |  |  |  |  |
| EPHX2 | Luminal epithelial cell |  |  |  |  |
| TRPM4 | Luminal epithelial cell |  |  |  |  |
| ASTN2 | Luminal epithelial cell |  |  |  |  |
| HID1 | Luminal epithelial cell |  |  |  |  |
| CSRP2 | Luminal epithelial cell |  |  |  |  |
| PRR15L | Luminal epithelial cell |  |  |  |  |
| RHPN2 | Luminal epithelial cell |  |  |  |  |
| TOM1L1 | Luminal epithelial cell |  |  |  |  |
| GCAT | Luminal epithelial cell |  |  |  |  |
| DPP4 | Luminal epithelial cell |  |  |  |  |
| FOXA1 | Luminal epithelial cell |  |  |  |  |
| GMPR | Luminal epithelial cell |  |  |  |  |
| ANXA3 | Luminal epithelial cell |  |  |  |  |
| DDAH1 | Luminal epithelial cell |  |  |  |  |
| TRIM36 | Luminal epithelial cell |  |  |  |  |
| MIPEP | Luminal epithelial cell |  |  |  |  |
| PRAC1 | Luminal epithelial cell |  |  |  |  |
| RASEF | Luminal epithelial cell |  |  |  |  |
| SMIM22 | Luminal epithelial cell |  |  |  |  |
| NECAB3 | Luminal epithelial cell |  |  |  |  |
| PLPP1 | Luminal epithelial cell |  |  |  |  |
| PRSS8 | Luminal epithelial cell |  |  |  |  |
| APOE | Myeloid cell |  |  |  |  |
| LILRB4 | Myeloid cell |  |  |  |  |
| OLR1 | Myeloid cell |  |  |  |  |
| CD163 | Myeloid cell |  |  |  |  |
| VSIG4 | Myeloid cell |  |  |  |  |
| CSF1R | Myeloid cell |  |  |  |  |
| SDS | Myeloid cell |  |  |  |  |
| C1QA | Myeloid cell |  |  |  |  |
| SLCO2B1 | Myeloid cell |  |  |  |  |
| C1QC | Myeloid cell |  |  |  |  |
| TREM2 | Myeloid cell |  |  |  |  |
| MSR1 | Myeloid cell |  |  |  |  |
| C5AR1 | Myeloid cell |  |  |  |  |
| LILRB2 | Myeloid cell |  |  |  |  |
| C1QB | Myeloid cell |  |  |  |  |
| CPVL | Myeloid cell |  |  |  |  |
| SLC8A1 | Myeloid cell |  |  |  |  |
| MS4A6A | Myeloid cell |  |  |  |  |
| MS4A7 | Myeloid cell |  |  |  |  |
| MPEG1 | Myeloid cell |  |  |  |  |
| CD86 | Myeloid cell |  |  |  |  |
| GPNMB | Myeloid cell |  |  |  |  |
| FCGR1A | Myeloid cell |  |  |  |  |
| IGSF6 | Myeloid cell |  |  |  |  |
| CD14 | Myeloid cell |  |  |  |  |
| CD68 | Myeloid cell |  |  |  |  |
| APOC1 | Myeloid cell |  |  |  |  |
| AIF1 | Myeloid cell |  |  |  |  |
| SLC7A7 | Myeloid cell |  |  |  |  |
| MS4A4A | Myeloid cell |  |  |  |  |
| FPR1 | Myeloid cell |  |  |  |  |
| ADAP2 | Myeloid cell |  |  |  |  |
| STAB1 | Myeloid cell |  |  |  |  |
| SPI1 | Myeloid cell |  |  |  |  |
| HMOX1 | Myeloid cell |  |  |  |  |
| PLD4 | Myeloid cell |  |  |  |  |
| LRRC25 | Myeloid cell |  |  |  |  |
| IFI30 | Myeloid cell |  |  |  |  |
| CYBB | Myeloid cell |  |  |  |  |
| FCGR2A | Myeloid cell |  |  |  |  |
| IL1B | Myeloid cell |  |  |  |  |
| CLEC7A | Myeloid cell |  |  |  |  |
| ADGRE2 | Myeloid cell |  |  |  |  |
| NCF2 | Myeloid cell |  |  |  |  |
| HCK | Myeloid cell |  |  |  |  |
| S100A8 | Myeloid cell |  |  |  |  |
| PILRA | Myeloid cell |  |  |  |  |
| PTAFR | Myeloid cell |  |  |  |  |
| PLAUR | Myeloid cell |  |  |  |  |
| MNDA | Myeloid cell |  |  |  |  |
| FCER1G | Myeloid cell |  |  |  |  |
| SIGLEC10 | Myeloid cell |  |  |  |  |
| LST1 | Myeloid cell |  |  |  |  |
| TGFBI | Myeloid cell |  |  |  |  |
| LYZ | Myeloid cell |  |  |  |  |
| TYROBP | Myeloid cell |  |  |  |  |
| IL18 | Myeloid cell |  |  |  |  |
| LY86 | Myeloid cell |  |  |  |  |
| RNASE6 | Myeloid cell |  |  |  |  |
| DOK3 | Myeloid cell |  |  |  |  |
| HLA-DRB5 | Myeloid cell |  |  |  |  |
| LHFPL2 | Myeloid cell |  |  |  |  |
| CSF3R | Myeloid cell |  |  |  |  |
| RAB31 | Myeloid cell |  |  |  |  |
| S100A9 | Myeloid cell |  |  |  |  |
| PHACTR1 | Myeloid cell |  |  |  |  |
| ITGAX | Myeloid cell |  |  |  |  |
| FGL2 | Myeloid cell |  |  |  |  |
| HLA-DQA1 | Myeloid cell |  |  |  |  |
| FCGR2B | Myeloid cell |  |  |  |  |
| HLA-DQB1 | Myeloid cell |  |  |  |  |
| HLA-DRA | Myeloid cell |  |  |  |  |
| SLC11A1 | Myeloid cell |  |  |  |  |
| TMEM176A | Myeloid cell |  |  |  |  |
| LILRB1 | Myeloid cell |  |  |  |  |
| HLA-DPA1 | Myeloid cell |  |  |  |  |
| MT1H | Myeloid cell |  |  |  |  |
| C1orf162 | Myeloid cell |  |  |  |  |
| HLA-DRB1 | Myeloid cell |  |  |  |  |
| MARCH1 | Myeloid cell |  |  |  |  |
| A2M | Myeloid cell |  |  |  |  |
| KCTD12 | Myeloid cell |  |  |  |  |
| FCGR3A | Myeloid cell |  |  |  |  |
| G0S2 | Myeloid cell |  |  |  |  |
| SIRPA | Myeloid cell |  |  |  |  |
| TMEM176B | Myeloid cell |  |  |  |  |
| PLXNC1 | Myeloid cell |  |  |  |  |
| RASSF4 | Myeloid cell |  |  |  |  |
| THEMIS2 | Myeloid cell |  |  |  |  |
| TNFSF13B | Myeloid cell |  |  |  |  |
| BCL2A1 | Myeloid cell |  |  |  |  |
| LY96 | Myeloid cell |  |  |  |  |
| SLC43A2 | Myeloid cell |  |  |  |  |
| CTSS | Myeloid cell |  |  |  |  |
| HLA-DMB | Myeloid cell |  |  |  |  |
| LGALS9 | Myeloid cell |  |  |  |  |
| HAVCR2 | Myeloid cell |  |  |  |  |
| GNB4 | Myeloid cell |  |  |  |  |
| CTSL | Myeloid cell |  |  |  |  |
| TIMP1 | Myeloid cell |  |  |  |  |
| SH2D1B | NK T cell |  |  |  |  |
| KLRF1 | NK T cell |  |  |  |  |
| KRT86 | NK T cell |  |  |  |  |
| TRDC | NK T cell |  |  |  |  |
| CD160 | NK T cell |  |  |  |  |
| TMIGD2 | NK T cell |  |  |  |  |
| GNLY | NK T cell |  |  |  |  |
| TXK | NK T cell |  |  |  |  |
| KLRC3 | NK T cell |  |  |  |  |
| FGFBP2 | NK T cell |  |  |  |  |
| CLIC3 | NK T cell |  |  |  |  |
| XCL2 | NK T cell |  |  |  |  |
| KLRD1 | NK T cell |  |  |  |  |
| XCL1 | NK T cell |  |  |  |  |
| CD247 | NK T cell |  |  |  |  |
| IL2RB | NK T cell |  |  |  |  |
| EOMES | NK T cell |  |  |  |  |
| CTSW | NK T cell |  |  |  |  |
| MATK | NK T cell |  |  |  |  |
| NKG7 | NK T cell |  |  |  |  |
| NMUR1 | NK T cell |  |  |  |  |
| KLRB1 | NK T cell |  |  |  |  |
| TNFRSF18 | NK T cell |  |  |  |  |
| FCRL6 | NK T cell |  |  |  |  |
| PRF1 | NK T cell |  |  |  |  |
| GZMA | NK T cell |  |  |  |  |
| TBX21 | NK T cell |  |  |  |  |
| KLRC1 | NK T cell |  |  |  |  |
| FASLG | NK T cell |  |  |  |  |
| HOPX | NK T cell |  |  |  |  |
| GZMB | NK T cell |  |  |  |  |
| CD7 | NK T cell |  |  |  |  |
| CST7 | NK T cell |  |  |  |  |
| CD244 | NK T cell |  |  |  |  |
| TRBC1 | NK T cell |  |  |  |  |
| PTGDR | NK T cell |  |  |  |  |
| HSPA6 | NK T cell |  |  |  |  |
| SAMD3 | NK T cell |  |  |  |  |
| CCL4 | NK T cell |  |  |  |  |
| PYHIN1 | NK T cell |  |  |  |  |
| CCL5 | NK T cell |  |  |  |  |
| GZMM | NK T cell |  |  |  |  |
| APOBEC3G | NK T cell |  |  |  |  |
| TOX | NK T cell |  |  |  |  |
| DUSP2 | NK T cell |  |  |  |  |
| LINC00861 | NK T cell |  |  |  |  |
| FGR | NK T cell |  |  |  |  |
| MCTP2 | NK T cell |  |  |  |  |
| AREG | NK T cell |  |  |  |  |
| ZAP70 | NK T cell |  |  |  |  |
| AOAH | NK T cell |  |  |  |  |
| IFITM1 | NK T cell |  |  |  |  |
| GZMH | NK T cell |  |  |  |  |
| CCL3 | NK T cell |  |  |  |  |
| HCST | NK T cell |  |  |  |  |
| ARHGAP9 | NK T cell |  |  |  |  |
| SH2D2A | NK T cell |  |  |  |  |
| GNG2 | NK T cell |  |  |  |  |
| BIN2 | NK T cell |  |  |  |  |
| CD300A | NK T cell |  |  |  |  |
| SPN | NK T cell |  |  |  |  |
| SKAP1 | NK T cell |  |  |  |  |
| ADAM8 | NK T cell |  |  |  |  |
| CLEC2B | NK T cell |  |  |  |  |
| SLAMF7 | NK T cell |  |  |  |  |
| DOK2 | NK T cell |  |  |  |  |
| ZNF683 | NK T cell |  |  |  |  |
| CHST12 | NK T cell |  |  |  |  |
| TGFB1 | NK T cell |  |  |  |  |
| KLF2 | NK T cell |  |  |  |  |
| TBC1D10C | NK T cell |  |  |  |  |
| GIMAP7 | NK T cell |  |  |  |  |
| ADGRG1 | NK T cell |  |  |  |  |
| RBM38 | NK T cell |  |  |  |  |
| CCDC69 | NK T cell |  |  |  |  |
| CLDND1 | NK T cell |  |  |  |  |
| LIMD2 | NK T cell |  |  |  |  |
| SOCS1 | NK T cell |  |  |  |  |
| ITGB7 | NK T cell |  |  |  |  |
| RNF125 | NK T cell |  |  |  |  |
| RHOF | NK T cell |  |  |  |  |
| ACAP1 | NK T cell |  |  |  |  |
| SYNE1 | NK T cell |  |  |  |  |
| APMAP | NK T cell |  |  |  |  |
| TAGAP | NK T cell |  |  |  |  |
| PIM1 | NK T cell |  |  |  |  |
| GZMK | NK T cell |  |  |  |  |
| STK17A | NK T cell |  |  |  |  |
| PTPN22 | NK T cell |  |  |  |  |
| LAT2 | NK T cell |  |  |  |  |
| GPR65 | NK T cell |  |  |  |  |
| IKZF3 | NK T cell |  |  |  |  |
| RAC2 | NK T cell |  |  |  |  |
| GYPC | NK T cell |  |  |  |  |
| IL16 | NK T cell |  |  |  |  |
| SH3BP1 | NK T cell |  |  |  |  |
| IL2RG | NK T cell |  |  |  |  |
| CORO1A | NK T cell |  |  |  |  |
| PSTPIP1 | NK T cell |  |  |  |  |
| FGD3 | NK T cell |  |  |  |  |
| CD5 | T cell |  |  |  |  |
| CAMK4 | T cell |  |  |  |  |
| THEMIS | T cell |  |  |  |  |
| CD8A | T cell |  |  |  |  |
| CD3G | T cell |  |  |  |  |
| CD6 | T cell |  |  |  |  |
| CD8B | T cell |  |  |  |  |
| CD3D | T cell |  |  |  |  |
| CD2 | T cell |  |  |  |  |
| ITK | T cell |  |  |  |  |
| SPOCK2 | T cell |  |  |  |  |
| ITGA1 | T cell |  |  |  |  |
| CXCR6 | T cell |  |  |  |  |
| SIT1 | T cell |  |  |  |  |
| CD96 | T cell |  |  |  |  |
| IL7R | T cell |  |  |  |  |
| CD3E | T cell |  |  |  |  |
| CXCR3 | T cell |  |  |  |  |
| SCML4 | T cell |  |  |  |  |
| STAT4 | T cell |  |  |  |  |
| GRAP2 | T cell |  |  |  |  |
| GZMH | T cell |  |  |  |  |
| ZNF683 | T cell |  |  |  |  |
| SKAP1 | T cell |  |  |  |  |
| TRAC | T cell |  |  |  |  |
| LCK | T cell |  |  |  |  |
| ZAP70 | T cell |  |  |  |  |
| CD27 | T cell |  |  |  |  |
| IKZF3 | T cell |  |  |  |  |
| FYN | T cell |  |  |  |  |
| TRBC2 | T cell |  |  |  |  |
| SH2D2A | T cell |  |  |  |  |
| CLEC2D | T cell |  |  |  |  |
| GZMM | T cell |  |  |  |  |
| IL32 | T cell |  |  |  |  |
| CCL5 | T cell |  |  |  |  |
| LAT | T cell |  |  |  |  |
| TBC1D10C | T cell |  |  |  |  |
| CD69 | T cell |  |  |  |  |
| ETS1 | T cell |  |  |  |  |
| PTPN22 | T cell |  |  |  |  |
| TRBC1 | T cell |  |  |  |  |
| CD7 | T cell |  |  |  |  |
| ACAP1 | T cell |  |  |  |  |
| SYTL3 | T cell |  |  |  |  |
| ITM2A | T cell |  |  |  |  |
| IL2RG | T cell |  |  |  |  |
| ITGAL | T cell |  |  |  |  |
| TRAF3IP3 | T cell |  |  |  |  |
| GZMB | T cell |  |  |  |  |
| PRF1 | T cell |  |  |  |  |
| GZMA | T cell |  |  |  |  |
| CISH | T cell |  |  |  |  |
| OXNAD1 | T cell |  |  |  |  |
| CST7 | T cell |  |  |  |  |
| CYTIP | T cell |  |  |  |  |
| PTPRC | T cell |  |  |  |  |
| CD247 | T cell |  |  |  |  |
| CD52 | T cell |  |  |  |  |
| CTSW | T cell |  |  |  |  |
| RHOH | T cell |  |  |  |  |
| APOBEC3G | T cell |  |  |  |  |
| HOPX | T cell |  |  |  |  |
| LTB | T cell |  |  |  |  |
| RHOF | T cell |  |  |  |  |
| ARAP2 | T cell |  |  |  |  |
| SELPLG | T cell |  |  |  |  |
| IKZF1 | T cell |  |  |  |  |
| IL2RB | T cell |  |  |  |  |
| PTPN7 | T cell |  |  |  |  |
| TMC8 | T cell |  |  |  |  |
| SPN | T cell |  |  |  |  |
| KLRD1 | T cell |  |  |  |  |
| RASAL3 | T cell |  |  |  |  |
| GBP5 | T cell |  |  |  |  |
| NKG7 | T cell |  |  |  |  |
| GIMAP7 | T cell |  |  |  |  |
| NFATC2 | T cell |  |  |  |  |
| PSTPIP1 | T cell |  |  |  |  |
| ARHGAP15 | T cell |  |  |  |  |
| CBLB | T cell |  |  |  |  |
| PTGER4 | T cell |  |  |  |  |
| CCND2 | T cell |  |  |  |  |
| PRDM1 | T cell |  |  |  |  |
| ANKRD44 | T cell |  |  |  |  |
| ITGB7 | T cell |  |  |  |  |
| RAC2 | T cell |  |  |  |  |
| PARP8 | T cell |  |  |  |  |
| CYFIP2 | T cell |  |  |  |  |
| HCST | T cell |  |  |  |  |
| IL16 | T cell |  |  |  |  |
| STK17A | T cell |  |  |  |  |
| CXCR4 | T cell |  |  |  |  |
| RORA | T cell |  |  |  |  |
| PIK3IP1 | T cell |  |  |  |  |
| TAGAP | T cell |  |  |  |  |
| FCMR | T cell |  |  |  |  |
| GNG2 | T cell |  |  |  |  |
| SOCS1 | T cell |  |  |  |  |
| SAMSN1 | T cell |  |  |  |  |

Table S9. Genes used to evaluate M1 and M2 macrophage polarization.

| M1 polarization | M2 polarization |
| --- | --- |
| CCL5 | CCL4 |
| CCR7 | CCL13 |
| CD40 | CCL18 |
| CD86 | CCL20 |
| CXCL9 | CCL22 |
| CXCL10 | CD276 |
| CXCL1 | CLEC7A |
| IDO1 | CTSB |
| IL1B | CTSD |
| IL6 | FN1 |
| IRF1 | IL4R |
| IRF5 | IRF4 |
| KYNU | LYVE1 |
| IL1A | MMP9 |
|  | MMP14 |
|  | MMP19 |
|  | MSR1 |
|  | TGFB1 |
|  | TGFB2 |
|  | TNFSF8 |
|  | TNFSF12 |
|  | VEGFA |
|  | VEGFB |
|  | VEGFC |
|  | CTSC |
|  | CTSA |

Table S10. Gene sets for evaluating T cell activation, function and subtypes in GEO datasets.

1. Gene expression for assessing subtypes of CD4 + T cells in GEO datasets.

| cluster 10 | | cluster 9 | | cluster 8 | | cluster 7 | | cluster 6 | | cluster 5 | | cluster 4 | | cluster 3 | | cluster 2 | | cluster 1 | | cluster 0 | |  |  |
| --- | --- | --- | --- | --- | --- | --- | --- | --- | --- | --- | --- | --- | --- | --- | --- | --- | --- | --- | --- | --- | --- | --- | --- |
| avg.exp | pct.exp | avg.exp | pct.exp | avg.exp | pct.exp | avg.exp | pct.exp | avg.exp | pct.exp | avg.exp | pct.exp | avg.exp | pct.exp | avg.exp | pct.exp | avg.exp | pct.exp | avg.exp | pct.exp | avg.exp | pct.exp | Gene |  |
| 1.86 | 25.35 | 1.27 | 16.67 | 0.89 | 14.45 | 0.96 | 14.23 | 2.44 | 33.20 | 0.68 | 9.77 | 0.23 | 3.49 | 1.81 | 24.12 | 3.77 | 48.68 | 1.19 | 16.75 | 2.03 | 25.82 | CCR7 | Naive |
| 1.19 | 20.33 | 0.58 | 9.41 | 1.99 | 24.39 | 0.81 | 13.21 | 1.74 | 27.91 | 0.39 | 7.19 | 0.06 | 1.39 | 0.51 | 9.60 | 2.24 | 37.08 | 1.58 | 25.99 | 1.13 | 17.05 | SELL |  |
| 0.86 | 17.55 | 0.71 | 14.12 | 0.97 | 15.93 | 0.69 | 14.14 | 1.21 | 23.96 | 0.45 | 9.81 | 0.45 | 9.21 | 0.88 | 17.93 | 1.35 | 27.85 | 0.43 | 8.88 | 1.15 | 21.80 | TCF7 |  |
| 13.02 | 81.62 | 16.97 | 87.45 | 0.90 | 16.22 | 15.62 | 84.19 | 7.35 | 64.93 | 11.85 | 73.30 | 19.82 | 91.06 | 16.87 | 90.85 | 10.96 | 82.41 | 4.69 | 52.13 | 16.98 | 90.17 | IL7R |  |
| 6.67 | 65.18 | 6.13 | 67.06 | 16.71 | 75.22 | 6.51 | 68.19 | 8.76 | 78.92 | 8.96 | 82.49 | 6.94 | 71.83 | 6.96 | 74.53 | 5.49 | 66.43 | 8.77 | 78.30 | 6.27 | 69.73 | PTPRC |  |
| 8.58 | 66.85 | 12.12 | 76.67 | 8.77 | 68.24 | 11.21 | 71.44 | 8.93 | 65.46 | 12.47 | 77.09 | 26.44 | 89.21 | 11.45 | 77.12 | 9.35 | 72.23 | 7.71 | 59.53 | 29.45 | 92.15 | CD69 | Early activation |
| 0.14 | 3.34 | 1.88 | 28.43 | 11.20 | 57.82 | 2.36 | 34.23 | 2.22 | 33.40 | 2.94 | 44.45 | 1.65 | 26.13 | 1.73 | 29.41 | 1.45 | 25.70 | 0.42 | 7.54 | 2.32 | 35.10 | CD40LG |  |
| 7.31 | 59.61 | 13.85 | 69.22 | 1.18 | 16.32 | 7.57 | 50.60 | 8.81 | 64.93 | 5.46 | 46.40 | 11.96 | 58.41 | 13.55 | 73.88 | 9.60 | 70.70 | 7.60 | 56.60 | 38.53 | 92.14 | FOS |  |
| 8.04 | 49.58 | 10.07 | 58.43 | 13.20 | 59.00 | 7.68 | 52.93 | 7.19 | 51.94 | 6.23 | 51.06 | 18.14 | 68.49 | 7.30 | 56.43 | 6.89 | 57.33 | 8.02 | 53.02 | 40.11 | 91.18 | JUN |  |
| 0.37 | 7.80 | 1.30 | 20.00 | 16.24 | 58.90 | 0.86 | 14.79 | 2.01 | 30.52 | 1.69 | 28.34 | 1.11 | 19.48 | 1.15 | 20.49 | 1.05 | 19.60 | 2.80 | 38.10 | 1.21 | 20.10 | ICOS | Effector |
| 0.64 | 9.19 | 1.35 | 19.61 | 1.54 | 20.35 | 1.30 | 21.67 | 2.73 | 37.15 | 2.33 | 33.42 | 2.40 | 35.31 | 1.06 | 17.56 | 0.69 | 13.94 | 5.70 | 57.40 | 0.94 | 16.30 | BATF |  |
| 0.19 | 3.34 | 0.26 | 3.53 | 1.82 | 20.75 | 0.13 | 2.70 | 0.25 | 5.49 | 0.27 | 5.63 | 0.29 | 6.06 | 0.21 | 4.00 | 0.16 | 3.52 | 0.43 | 8.88 | 0.18 | 3.62 | IRF4 |  |
| 0.69 | 10.31 | 1.58 | 19.61 | 0.25 | 3.64 | 1.42 | 18.05 | 0.26 | 4.28 | 4.33 | 46.05 | 2.56 | 32.50 | 1.15 | 18.83 | 0.27 | 4.94 | 0.41 | 6.12 | 1.39 | 18.73 | GZMA | Cyto |
| 1.14 | 11.42 | 0.35 | 4.51 | 0.57 | 6.88 | 0.38 | 3.63 | 0.10 | 2.48 | 0.61 | 7.54 | 0.73 | 9.37 | 0.23 | 3.35 | 0.11 | 2.60 | 0.23 | 3.34 | 0.30 | 4.69 | GZMB |  |
| 0.55 | 6.69 | 0.23 | 4.51 | 0.43 | 2.46 | 0.27 | 4.56 | 0.11 | 2.88 | 0.32 | 5.75 | 0.58 | 11.50 | 0.22 | 4.14 | 0.05 | 1.13 | 0.26 | 6.10 | 0.23 | 4.62 | PRF1 |  |
| 2.45 | 28.69 | 0.80 | 10.78 | 0.22 | 2.46 | 0.50 | 7.81 | 0.17 | 3.68 | 0.36 | 7.00 | 2.51 | 37.07 | 0.57 | 9.18 | 0.17 | 3.33 | 0.26 | 4.87 | 0.53 | 8.88 | NKG7 |  |
| 2.71 | 13.37 | 0.86 | 6.47 | 0.77 | 7.47 | 0.51 | 4.09 | 0.21 | 3.95 | 0.50 | 5.28 | 0.69 | 7.73 | 0.46 | 4.90 | 0.17 | 3.57 | 0.36 | 4.95 | 0.63 | 5.96 | GNLY |  |
| 0.28 | 4.18 | 0.57 | 10.78 | 0.51 | 3.05 | 0.64 | 11.16 | 1.06 | 20.48 | 1.31 | 22.44 | 1.28 | 23.01 | 0.63 | 11.40 | 0.19 | 4.16 | 0.53 | 10.46 | 0.48 | 9.17 | PDCD1 | Exhaust |
| 0.11 | 2.79 | 0.09 | 1.96 | 0.29 | 5.11 | 0.14 | 2.33 | 0.14 | 2.81 | 0.28 | 6.06 | 0.16 | 3.59 | 0.07 | 1.52 | 0.04 | 0.97 | 0.40 | 8.33 | 0.10 | 2.18 | HAVCR2 |  |
| 0.54 | 8.64 | 0.21 | 3.14 | 0.10 | 1.57 | 0.28 | 4.84 | 0.14 | 3.08 | 0.32 | 6.57 | 0.65 | 13.05 | 0.21 | 4.25 | 0.07 | 1.42 | 0.24 | 4.47 | 0.18 | 3.73 | LAG3 |  |
| 0.35 | 6.69 | 0.27 | 4.90 | 0.28 | 3.54 | 0.37 | 6.05 | 2.74 | 39.76 | 0.30 | 6.06 | 0.31 | 6.06 | 0.20 | 4.11 | 0.48 | 9.94 | 3.81 | 48.46 | 0.29 | 5.82 | TIGIT |  |
| 0.08 | 1.11 | 0.11 | 1.96 | 2.04 | 20.55 | 0.09 | 1.67 | 0.54 | 12.05 | 0.23 | 4.96 | 0.42 | 9.18 | 0.11 | 2.31 | 0.08 | 1.83 | 0.41 | 8.43 | 0.09 | 1.99 | TOX |  |
| 0.10 | 2.23 | 0.20 | 3.92 | 0.27 | 4.03 | 0.26 | 4.84 | 0.56 | 11.31 | 0.77 | 13.80 | 1.46 | 23.59 | 0.18 | 3.29 | 0.06 | 1.34 | 1.17 | 20.57 | 0.16 | 2.78 | ENTPD1 |  |
| 0.00 | 0.00 | 0.00 | 0.20 | 0.21 | 3.83 | 0.03 | 0.56 | 0.04 | 0.94 | 0.01 | 0.23 | 0.02 | 0.53 | 0.01 | 0.17 | 0.02 | 0.40 | 0.69 | 13.47 | 0.01 | 0.21 | FOXP3 | Treg |
| 0.15 | 2.79 | 0.26 | 5.29 | 0.14 | 2.26 | 0.33 | 6.33 | 0.20 | 4.08 | 0.19 | 3.83 | 0.17 | 3.74 | 0.36 | 7.54 | 0.29 | 6.28 | 2.83 | 32.53 | 0.30 | 6.23 | IL2RA |  |
| 0.15 | 2.79 | 0.54 | 11.18 | 0.47 | 7.08 | 0.63 | 12.47 | 1.96 | 27.58 | 1.38 | 23.03 | 1.24 | 20.10 | 0.59 | 10.39 | 0.40 | 7.52 | 3.71 | 42.75 | 0.60 | 9.47 | CTLA4 |  |
| 0.07 | 1.11 | 0.60 | 10.78 | 1.17 | 13.67 | 0.79 | 14.51 | 1.95 | 30.05 | 0.85 | 15.36 | 0.35 | 6.28 | 0.78 | 15.45 | 0.26 | 6.20 | 4.04 | 41.40 | 0.40 | 8.34 | TNFRSF18 |  |
| 1.68 | 29.53 | 0.65 | 11.18 | 1.22 | 16.22 | 0.98 | 17.49 | 2.64 | 39.02 | 0.57 | 11.42 | 0.37 | 7.08 | 0.64 | 13.20 | 1.17 | 25.46 | 4.61 | 53.99 | 1.02 | 19.80 | CD27 | Memory |
| 0.26 | 5.29 | 0.45 | 9.41 | 0.92 | 15.73 | 0.45 | 9.77 | 0.95 | 20.95 | 0.52 | 11.22 | 0.19 | 4.39 | 0.50 | 11.82 | 0.51 | 12.35 | 0.79 | 16.13 | 0.39 | 8.91 | CD28 |  |
| 23.72 | 91.92 | 29.43 | 96.86 | 0.91 | 13.77 | 24.48 | 91.26 | 24.47 | 91.50 | 33.88 | 95.74 | 47.53 | 97.93 | 29.72 | 96.68 | 19.28 | 92.16 | 19.45 | 82.10 | 55.22 | 98.05 | CXCR4 |  |
| 0.36 | 4.46 | 2.49 | 32.16 | 27.59 | 87.81 | 1.82 | 28.56 | 1.05 | 16.60 | 2.49 | 34.83 | 3.32 | 45.64 | 2.91 | 40.22 | 0.97 | 15.98 | 1.84 | 25.61 | 1.67 | 24.48 | CCR6 |  |

1. Gene expression in assessing function of CD4 + subtype cells in GEO datasets.

| cluster 10 | | cluster 9 | | cluster 8 | | cluster 7 | | cluster 6 | | cluster 5 | | cluster 4 | | cluster 3 | | cluster 2 | | cluster 1 | | cluster 0 | |  |  |
| --- | --- | --- | --- | --- | --- | --- | --- | --- | --- | --- | --- | --- | --- | --- | --- | --- | --- | --- | --- | --- | --- | --- | --- |
| avg.exp | pct.exp | avg.exp | pct.exp | avg.exp | pct.exp | avg.exp | pct.exp | avg.exp | pct.exp | avg.exp | pct.exp | avg.exp | pct.exp | avg.exp | pct.exp | avg.exp | pct.exp | avg.exp | pct.exp | avg.exp | pct.exp | Gene |  |
| 1.86 | 25.35 | 1.27 | 16.67 | 1.99 | 24.39 | 0.96 | 14.23 | 2.44 | 33.20 | 0.68 | 9.77 | 0.23 | 3.49 | 1.81 | 24.12 | 3.77 | 48.68 | 1.19 | 16.75 | 2.03 | 25.82 | CCR7 | Naive |
| 1.19 | 20.33 | 0.58 | 9.41 | 0.97 | 15.93 | 0.81 | 13.21 | 1.74 | 27.91 | 0.39 | 7.19 | 0.06 | 1.39 | 0.51 | 9.60 | 2.24 | 37.08 | 1.58 | 25.99 | 1.13 | 17.05 | SELL |  |
| 13.02 | 81.62 | 16.97 | 87.45 | 16.71 | 75.22 | 15.62 | 84.19 | 7.35 | 64.93 | 11.85 | 73.30 | 19.82 | 91.06 | 16.87 | 90.85 | 10.96 | 82.41 | 4.69 | 52.13 | 16.98 | 90.17 | IL7R |  |
| 0.86 | 17.55 | 0.71 | 14.12 | 0.90 | 16.22 | 0.69 | 14.14 | 1.21 | 23.96 | 0.45 | 9.81 | 0.45 | 9.21 | 0.88 | 17.93 | 1.35 | 27.85 | 0.43 | 8.88 | 1.15 | 21.80 | TCF7 |  |
| 6.67 | 65.18 | 6.13 | 67.06 | 8.77 | 68.24 | 6.51 | 68.19 | 8.76 | 78.92 | 8.96 | 82.49 | 6.94 | 71.83 | 6.96 | 74.53 | 5.49 | 66.43 | 8.77 | 78.30 | 6.27 | 69.73 | PTPRC |  |
| 8.58 | 66.85 | 12.12 | 76.67 | 11.20 | 57.82 | 11.21 | 71.44 | 8.93 | 65.46 | 12.47 | 77.09 | 26.44 | 89.21 | 11.45 | 77.12 | 9.35 | 72.23 | 7.71 | 59.53 | 29.45 | 92.15 | CD69 |  |
| 1.68 | 29.53 | 0.65 | 11.18 | 0.92 | 15.73 | 0.98 | 17.49 | 2.64 | 39.02 | 0.57 | 11.42 | 0.37 | 7.08 | 0.64 | 13.20 | 1.17 | 25.46 | 4.61 | 53.99 | 1.02 | 19.80 | CD27 | Memory |
| 0.26 | 5.29 | 0.45 | 9.41 | 0.91 | 13.77 | 0.45 | 9.77 | 0.95 | 20.95 | 0.52 | 11.22 | 0.19 | 4.39 | 0.50 | 11.82 | 0.51 | 12.35 | 0.79 | 16.13 | 0.39 | 8.91 | CD28 |  |
| 23.72 | 91.92 | 29.43 | 96.86 | 27.59 | 87.81 | 24.48 | 91.26 | 24.47 | 91.50 | 33.88 | 95.74 | 47.53 | 97.93 | 29.72 | 96.68 | 19.28 | 92.16 | 19.45 | 82.10 | 55.22 | 98.05 | CXCR4 |  |
| 0.36 | 4.46 | 2.49 | 32.16 | 0.89 | 14.45 | 1.82 | 28.56 | 1.05 | 16.60 | 2.49 | 34.83 | 3.32 | 45.64 | 2.91 | 40.22 | 0.97 | 15.98 | 1.84 | 25.61 | 1.67 | 24.48 | CCR6 |  |
| 0.14 | 3.34 | 1.88 | 28.43 | 1.18 | 16.32 | 2.36 | 34.23 | 2.22 | 33.40 | 2.94 | 44.45 | 1.65 | 26.13 | 1.73 | 29.41 | 1.45 | 25.70 | 0.42 | 7.54 | 2.32 | 35.10 | CD40LG | Helper |
| 0.37 | 7.80 | 1.30 | 20.00 | 1.54 | 20.35 | 0.86 | 14.79 | 2.01 | 30.52 | 1.69 | 28.34 | 1.11 | 19.48 | 1.15 | 20.49 | 1.05 | 19.60 | 2.80 | 38.10 | 1.21 | 20.10 | ICOS |  |
| 0.64 | 9.19 | 1.35 | 19.61 | 1.82 | 20.75 | 1.30 | 21.67 | 2.73 | 37.15 | 2.33 | 33.42 | 2.40 | 35.31 | 1.06 | 17.56 | 0.69 | 13.94 | 5.70 | 57.40 | 0.94 | 16.30 | BATF |  |
| 0.19 | 3.34 | 0.26 | 3.53 | 0.25 | 3.64 | 0.13 | 2.70 | 0.25 | 5.49 | 0.27 | 5.63 | 0.29 | 6.06 | 0.21 | 4.00 | 0.16 | 3.52 | 0.43 | 8.88 | 0.18 | 3.62 | IRF4 |  |
| 0.69 | 10.31 | 1.58 | 19.61 | 0.57 | 6.88 | 1.42 | 18.05 | 0.26 | 4.28 | 4.33 | 46.05 | 2.56 | 32.50 | 1.15 | 18.83 | 0.27 | 4.94 | 0.41 | 6.12 | 1.39 | 18.73 | GZMA | Cytotoxic |
| 1.14 | 11.42 | 0.35 | 4.51 | 0.43 | 2.46 | 0.38 | 3.63 | 0.10 | 2.48 | 0.61 | 7.54 | 0.73 | 9.37 | 0.23 | 3.35 | 0.11 | 2.60 | 0.23 | 3.34 | 0.30 | 4.69 | GZMB |  |
| 0.55 | 6.69 | 0.23 | 4.51 | 0.22 | 2.46 | 0.27 | 4.56 | 0.11 | 2.88 | 0.32 | 5.75 | 0.58 | 11.50 | 0.22 | 4.14 | 0.05 | 1.13 | 0.26 | 6.10 | 0.23 | 4.62 | PRF1 |  |
| 0.24 | 4.46 | 0.36 | 6.86 | 0.40 | 5.51 | 0.52 | 9.67 | 0.13 | 3.28 | 0.29 | 5.90 | 0.20 | 4.08 | 0.46 | 9.26 | 0.24 | 5.50 | 0.13 | 3.02 | 0.52 | 10.28 | KLRG1 |  |
| 2.45 | 28.69 | 0.80 | 10.78 | 0.77 | 7.47 | 0.50 | 7.81 | 0.17 | 3.68 | 0.36 | 7.00 | 2.51 | 37.07 | 0.57 | 9.18 | 0.17 | 3.33 | 0.26 | 4.87 | 0.53 | 8.88 | NKG7 |  |
| 0.00 | 0.00 | 0.01 | 0.20 | 0.02 | 0.29 | 0.00 | 0.00 | 0.00 | 0.07 | 0.00 | 0.08 | 0.00 | 0.03 | 0.01 | 0.14 | 0.00 | 0.05 | 0.02 | 0.58 | 0.00 | 0.05 | CX3CR1 |  |
| 0.28 | 4.18 | 0.57 | 10.78 | 0.29 | 5.11 | 0.64 | 11.16 | 1.06 | 20.48 | 1.31 | 22.44 | 1.28 | 23.01 | 0.63 | 11.40 | 0.19 | 4.16 | 0.53 | 10.46 | 0.48 | 9.17 | PDCD1 | Exhaustion |
| 0.11 | 2.79 | 0.09 | 1.96 | 0.10 | 1.57 | 0.14 | 2.33 | 0.14 | 2.81 | 0.28 | 6.06 | 0.16 | 3.59 | 0.07 | 1.52 | 0.04 | 0.97 | 0.40 | 8.33 | 0.10 | 2.18 | HAVCR2 |  |
| 0.54 | 8.64 | 0.21 | 3.14 | 0.28 | 3.54 | 0.28 | 4.84 | 0.14 | 3.08 | 0.32 | 6.57 | 0.65 | 13.05 | 0.21 | 4.25 | 0.07 | 1.42 | 0.24 | 4.47 | 0.18 | 3.73 | LAG3 |  |
| 0.35 | 6.69 | 0.27 | 4.90 | 2.04 | 20.55 | 0.37 | 6.05 | 2.74 | 39.76 | 0.30 | 6.06 | 0.31 | 6.06 | 0.20 | 4.11 | 0.48 | 9.94 | 3.81 | 48.46 | 0.29 | 5.82 | TIGIT |  |
| 0.08 | 1.11 | 0.11 | 1.96 | 0.27 | 4.03 | 0.09 | 1.67 | 0.54 | 12.05 | 0.23 | 4.96 | 0.42 | 9.18 | 0.11 | 2.31 | 0.08 | 1.83 | 0.41 | 8.43 | 0.09 | 1.99 | TOX |  |
| 0.10 | 2.23 | 0.20 | 3.92 | 0.21 | 3.83 | 0.26 | 4.84 | 0.56 | 11.31 | 0.77 | 13.80 | 1.46 | 23.59 | 0.18 | 3.29 | 0.06 | 1.34 | 1.17 | 20.57 | 0.16 | 2.78 | ENTPD1 |  |
| 0.00 | 0.00 | 0.00 | 0.20 | 0.14 | 2.26 | 0.03 | 0.56 | 0.04 | 0.94 | 0.01 | 0.23 | 0.02 | 0.53 | 0.01 | 0.17 | 0.02 | 0.40 | 0.69 | 13.47 | 0.01 | 0.21 | FOXP3 | Treg |
| 0.15 | 2.79 | 0.26 | 5.29 | 0.47 | 7.08 | 0.33 | 6.33 | 0.20 | 4.08 | 0.19 | 3.83 | 0.17 | 3.74 | 0.36 | 7.54 | 0.29 | 6.28 | 2.83 | 32.53 | 0.30 | 6.23 | IL2RA |  |
| 0.15 | 2.79 | 0.54 | 11.18 | 1.17 | 13.67 | 0.63 | 12.47 | 1.96 | 27.58 | 1.38 | 23.03 | 1.24 | 20.10 | 0.59 | 10.39 | 0.40 | 7.52 | 3.71 | 42.75 | 0.60 | 9.47 | CTLA4 |  |
| 0.07 | 1.11 | 0.60 | 10.78 | 1.22 | 16.22 | 0.79 | 14.51 | 1.95 | 30.05 | 0.85 | 15.36 | 0.35 | 6.28 | 0.78 | 15.45 | 0.26 | 6.20 | 4.04 | 41.40 | 0.40 | 8.34 | TNFRSF18 |  |

1. Gene expression for assessing activation of CD8 + T cells in GEO datasets

| cluster 10 | | cluster 9 | | cluster 8 | | cluster 7 | | cluster 6 | | cluster 5 | | cluster 4 | | cluster 3 | | cluster 2 | | cluster 1 | | cluster 0 | |  |  |
| --- | --- | --- | --- | --- | --- | --- | --- | --- | --- | --- | --- | --- | --- | --- | --- | --- | --- | --- | --- | --- | --- | --- | --- |
| avg.exp | pct.exp | avg.exp | pct.exp | avg.exp | pct.exp | avg.exp | pct.exp | avg.exp | pct.exp | avg.exp | pct.exp | avg.exp | pct.exp | avg.exp | pct.exp | avg.exp | pct.exp | avg.exp | pct.exp | avg.exp | pct.exp | Gene |  |
| 0.83 | 12.21 | 0.16 | 2.16 | 0.61 | 6.64 | 0.55 | 10.05 | 3.04 | 35.13 | 1.03 | 11.72 | 1.54 | 19.75 | 0.13 | 2.40 | 0.47 | 7.32 | 0.15 | 2.60 | 0.22 | 3.49 | CCR7 | Naive |
| 0.72 | 16.14 | 0.51 | 9.42 | 0.70 | 12.73 | 0.52 | 12.90 | 1.07 | 20.60 | 0.72 | 12.85 | 1.03 | 18.89 | 0.51 | 9.85 | 0.75 | 12.34 | 0.51 | 10.40 | 0.56 | 10.33 | TCF7 |  |
| 0.36 | 7.00 | 0.04 | 1.04 | 0.25 | 4.94 | 0.16 | 5.25 | 0.77 | 16.05 | 0.10 | 2.16 | 0.13 | 2.97 | 0.06 | 1.31 | 0.08 | 1.87 | 0.05 | 1.20 | 0.06 | 1.42 | LEF1 |  |
| 0.46 | 8.28 | 0.08 | 1.44 | 0.45 | 6.64 | 0.31 | 7.33 | 1.98 | 30.61 | 0.29 | 4.80 | 0.51 | 8.22 | 0.06 | 0.98 | 0.26 | 4.32 | 0.05 | 1.05 | 0.07 | 1.48 | SELL |  |
| 12.05 | 57.81 | 12.28 | 73.08 | 8.13 | 59.72 | 11.54 | 71.76 | 11.28 | 76.97 | 5.73 | 45.20 | 16.33 | 68.66 | 10.04 | 70.22 | 3.77 | 30.50 | 11.91 | 74.90 | 13.08 | 76.25 | IL7R |  |
| 7.71 | 75.66 | 8.97 | 74.60 | 5.33 | 58.95 | 7.60 | 73.17 | 5.35 | 59.81 | 5.61 | 60.15 | 12.83 | 82.18 | 6.67 | 68.91 | 11.47 | 80.81 | 8.19 | 77.43 | 9.60 | 78.58 | PTPRC |  |
| 24.31 | 73.53 | 21.01 | 84.66 | 31.43 | 84.34 | 16.24 | 83.08 | 12.77 | 66.99 | 20.59 | 75.31 | 7.48 | 61.44 | 37.66 | 94.85 | 17.11 | 78.12 | 33.22 | 95.35 | 13.55 | 80.43 | CD69 | Resident |
| 2.28 | 36.29 | 3.85 | 48.80 | 1.80 | 27.62 | 2.01 | 33.98 | 1.33 | 21.68 | 2.47 | 34.90 | 3.48 | 44.75 | 2.99 | 42.33 | 3.16 | 40.65 | 3.43 | 49.32 | 3.82 | 48.25 | RUNX3 |  |
| 4.46 | 45.00 | 1.36 | 18.37 | 7.27 | 61.50 | 0.96 | 16.02 | 4.39 | 48.35 | 5.95 | 54.38 | 0.85 | 13.59 | 3.02 | 38.01 | 1.20 | 16.67 | 1.91 | 29.29 | 0.64 | 11.05 | NR4A1 |  |
| 0.93 | 15.71 | 0.70 | 11.98 | 0.80 | 13.04 | 0.67 | 12.17 | 1.34 | 19.72 | 1.17 | 18.03 | 1.22 | 17.55 | 0.50 | 9.69 | 1.90 | 26.85 | 0.73 | 14.87 | 0.65 | 11.18 | TIGIT | Inhibitor |
| 0.18 | 3.67 | 0.23 | 4.31 | 0.10 | 1.70 | 0.21 | 4.98 | 0.78 | 11.29 | 0.14 | 2.34 | 0.36 | 6.19 | 0.10 | 1.88 | 0.32 | 4.93 | 0.26 | 4.78 | 0.20 | 3.51 | CTLA4 |  |
| 0.52 | 11.02 | 1.24 | 20.93 | 0.74 | 12.35 | 0.71 | 13.35 | 0.19 | 3.24 | 0.85 | 13.77 | 0.52 | 9.06 | 0.86 | 16.28 | 1.00 | 16.23 | 1.14 | 22.25 | 1.11 | 18.73 | LAG3 |  |
| 0.41 | 8.20 | 0.97 | 15.73 | 0.35 | 6.71 | 0.63 | 11.81 | 0.41 | 7.55 | 0.68 | 10.66 | 0.45 | 8.27 | 0.57 | 10.63 | 0.83 | 13.07 | 0.99 | 18.41 | 0.99 | 15.69 | PDCD1 |  |
| 0.09 | 1.79 | 0.21 | 3.35 | 0.09 | 1.70 | 0.19 | 3.89 | 0.08 | 1.69 | 0.12 | 2.16 | 0.09 | 1.73 | 0.13 | 2.34 | 0.15 | 2.35 | 0.17 | 3.52 | 0.19 | 3.47 | HAVCR2 |  |
| 2.45 | 9.05 | 7.67 | 30.19 | 5.57 | 20.37 | 9.80 | 30.32 | 0.82 | 5.77 | 4.26 | 17.14 | 3.75 | 12.95 | 10.85 | 33.68 | 1.85 | 8.25 | 10.76 | 36.92 | 9.40 | 34.22 | GNLY | Cytokines |
| 1.27 | 11.10 | 8.96 | 55.59 | 1.71 | 16.05 | 5.12 | 38.96 | 0.29 | 2.87 | 2.42 | 19.12 | 4.16 | 19.68 | 8.06 | 51.69 | 2.28 | 17.73 | 13.15 | 69.16 | 9.48 | 59.81 | GZMB |  |
| 7.85 | 42.78 | 0.95 | 10.14 | 4.48 | 27.08 | 1.51 | 15.88 | 1.02 | 10.35 | 7.06 | 48.64 | 3.81 | 31.29 | 1.27 | 12.47 | 12.87 | 79.17 | 0.92 | 10.79 | 0.71 | 8.95 | GZMK |  |
| 10.93 | 34.33 | 4.93 | 30.19 | 12.08 | 38.97 | 1.91 | 17.15 | 2.15 | 12.64 | 12.60 | 43.55 | 3.38 | 24.18 | 11.66 | 49.31 | 5.74 | 32.22 | 12.11 | 54.34 | 1.40 | 15.11 | IFNG |  |
| 4.95 | 42.10 | 3.05 | 33.95 | 3.42 | 32.18 | 4.16 | 39.73 | 0.61 | 7.75 | 4.12 | 40.85 | 1.39 | 18.37 | 4.31 | 40.99 | 8.97 | 67.90 | 4.82 | 47.61 | 3.27 | 35.20 | GZMA |  |
| 5.20 | 42.19 | 5.34 | 56.07 | 4.66 | 44.44 | 5.74 | 55.02 | 0.63 | 7.89 | 7.05 | 57.05 | 4.10 | 34.95 | 5.30 | 59.22 | 12.26 | 74.75 | 5.64 | 63.57 | 5.62 | 58.01 | NKG7 |  |
| 0.64 | 13.92 | 0.60 | 10.86 | 0.73 | 13.97 | 0.89 | 19.37 | 0.91 | 19.76 | 0.62 | 11.28 | 0.48 | 9.88 | 0.71 | 13.91 | 1.02 | 17.58 | 0.59 | 13.06 | 0.66 | 12.36 | TNFRSF14 | Co_stim |
| 0.42 | 9.39 | 0.13 | 2.56 | 0.23 | 3.86 | 0.16 | 4.43 | 0.70 | 13.52 | 0.37 | 6.69 | 0.65 | 12.18 | 0.09 | 1.86 | 0.56 | 9.84 | 0.10 | 2.15 | 0.12 | 2.52 | CD28 |  |
| 0.92 | 15.63 | 1.31 | 18.69 | 0.78 | 12.11 | 0.54 | 11.27 | 1.29 | 18.11 | 0.95 | 13.50 | 1.06 | 16.11 | 0.71 | 11.77 | 1.03 | 14.98 | 1.56 | 24.71 | 0.86 | 13.74 | ICOS |  |
| 0.47 | 8.88 | 0.28 | 3.99 | 0.26 | 4.09 | 0.13 | 2.22 | 0.99 | 9.88 | 1.18 | 13.97 | 1.90 | 18.91 | 0.09 | 1.70 | 1.16 | 14.52 | 0.21 | 3.70 | 0.22 | 3.43 | TNFRSF9 |  |
| 0.36 | 8.54 | 0.26 | 5.11 | 0.44 | 7.72 | 0.35 | 9.19 | 0.52 | 10.82 | 0.13 | 2.66 | 0.20 | 4.36 | 0.32 | 6.35 | 0.12 | 2.56 | 0.22 | 5.04 | 0.18 | 3.51 | TNFRSF25 |  |
| 0.02 | 0.51 | 0.01 | 0.24 | 0.01 | 0.23 | 0.02 | 0.27 | 0.08 | 1.69 | 0.00 | 0.06 | 0.01 | 0.20 | 0.01 | 0.22 | 0.00 | 0.10 | 0.00 | 0.10 | 0.01 | 0.14 | FOXP3 | Treg |
| 0.10 | 2.48 | 0.09 | 1.68 | 0.06 | 1.39 | 0.09 | 2.53 | 0.35 | 6.44 | 0.07 | 1.24 | 0.17 | 2.92 | 0.07 | 1.40 | 0.03 | 0.42 | 0.06 | 1.49 | 0.05 | 1.04 | IL2RA |  |
| 0.16 | 3.16 | 0.02 | 0.48 | 0.06 | 1.16 | 0.06 | 1.22 | 0.22 | 4.55 | 0.08 | 1.54 | 0.06 | 1.11 | 0.03 | 0.59 | 0.14 | 2.38 | 0.02 | 0.49 | 0.02 | 0.45 | IKZF2 |  |
|  |  |  |  |  |  |  |  |  |  |  |  |  |  |  |  |  |  |  |  |  |  |  |  |
|  |  |  |  |  |  |  |  | cluster 17 | | cluster 16 | | cluster 15 | | cluster 14 | | cluster 13 | | cluster 12 | | cluster 11 | |  |  |
|  |  |  |  |  |  |  |  | avg.exp | pct.exp | avg.exp | pct.exp | avg.exp | pct.exp | avg.exp | pct.exp | avg.exp | pct.exp | avg.exp | pct.exp | avg.exp | pct.exp | Gene |  |
|  |  |  |  |  |  |  |  | 1.13 | 14.96 | 0.32 | 4.19 | 0.62 | 10.75 | 1.61 | 10.96 | 1.87 | 20.83 | 0.11 | 1.83 | 0.35 | 4.88 | CCR7 | Naive |
|  |  |  |  |  |  |  |  | 1.35 | 22.09 | 0.67 | 11.63 | 0.99 | 19.85 | 0.89 | 12.79 | 0.73 | 14.71 | 0.85 | 16.46 | 0.54 | 9.59 | TCF7 |  |
|  |  |  |  |  |  |  |  | 0.07 | 1.19 | 0.06 | 0.70 | 0.16 | 3.64 | 0.12 | 1.99 | 0.12 | 2.57 | 0.29 | 5.49 | 0.02 | 0.46 | LEF1 |  |
|  |  |  |  |  |  |  |  | 0.48 | 6.41 | 0.03 | 0.70 | 0.22 | 4.19 | 0.34 | 4.32 | 0.22 | 4.17 | 0.15 | 2.80 | 0.06 | 1.11 | SELL |  |
|  |  |  |  |  |  |  |  | 26.47 | 84.32 | 6.07 | 49.53 | 11.87 | 68.85 | 12.34 | 47.51 | 19.18 | 87.25 | 6.64 | 47.93 | 8.13 | 57.24 | IL7R |  |
|  |  |  |  |  |  |  |  | 4.73 | 57.72 | 6.42 | 68.37 | 9.84 | 81.60 | 12.52 | 65.78 | 5.59 | 62.25 | 8.52 | 72.56 | 6.35 | 63.50 | PTPRC |  |
|  |  |  |  |  |  |  |  | 23.39 | 78.15 | 43.61 | 89.77 | 16.73 | 77.41 | 9.54 | 48.34 | 22.33 | 79.41 | 23.37 | 86.46 | 17.19 | 78.43 | CD69 | Resident |
|  |  |  |  |  |  |  |  | 1.16 | 21.38 | 2.69 | 39.53 | 3.08 | 43.90 | 3.33 | 33.22 | 1.85 | 29.53 | 2.72 | 39.15 | 3.17 | 41.38 | RUNX3 |  |
|  |  |  |  |  |  |  |  | 4.26 | 40.62 | 7.66 | 65.35 | 2.26 | 31.51 | 3.89 | 26.08 | 4.68 | 49.63 | 1.46 | 22.07 | 3.09 | 35.02 | NR4A1 |  |
|  |  |  |  |  |  |  |  | 0.15 | 3.33 | 0.95 | 13.49 | 0.98 | 16.94 | 1.90 | 21.59 | 0.21 | 4.78 | 0.96 | 15.00 | 0.69 | 12.35 | TIGIT | Inhibitor |
|  |  |  |  |  |  |  |  | 0.10 | 2.38 | 0.08 | 1.16 | 0.31 | 5.10 | 0.89 | 9.14 | 0.39 | 6.13 | 0.09 | 1.83 | 0.08 | 1.47 | CTLA4 |  |
|  |  |  |  |  |  |  |  | 0.13 | 2.61 | 1.18 | 18.37 | 0.81 | 14.94 | 0.46 | 6.81 | 0.37 | 6.25 | 0.74 | 13.78 | 1.05 | 18.89 | LAG3 |  |
|  |  |  |  |  |  |  |  | 0.07 | 0.95 | 0.55 | 10.00 | 0.75 | 15.30 | 0.31 | 4.82 | 0.54 | 10.66 | 0.14 | 2.20 | 0.64 | 10.32 | PDCD1 |  |
|  |  |  |  |  |  |  |  | 0.45 | 7.60 | 0.11 | 2.33 | 0.14 | 3.10 | 0.17 | 1.50 | 0.08 | 1.72 | 0.29 | 5.12 | 0.11 | 2.30 | HAVCR2 |  |
|  |  |  |  |  |  |  |  | 4.68 | 17.34 | 11.09 | 32.56 | 2.75 | 13.66 | 1.75 | 7.81 | 0.87 | 6.74 | 31.72 | 59.76 | 9.69 | 36.04 | GNLY | Cytokines |
|  |  |  |  |  |  |  |  | 0.88 | 7.60 | 5.55 | 39.30 | 4.06 | 31.33 | 2.44 | 8.97 | 0.53 | 4.41 | 7.19 | 42.20 | 7.20 | 46.73 | GZMB |  |
|  |  |  |  |  |  |  |  | 0.80 | 8.08 | 4.49 | 29.53 | 3.27 | 30.42 | 7.77 | 36.71 | 0.56 | 7.72 | 1.61 | 17.44 | 2.16 | 18.43 | GZMK |  |
|  |  |  |  |  |  |  |  | 1.38 | 8.55 | 23.83 | 60.23 | 3.42 | 25.32 | 5.47 | 19.77 | 4.46 | 17.65 | 5.03 | 30.00 | 8.09 | 38.06 | IFNG |  |
|  |  |  |  |  |  |  |  | 1.05 | 10.21 | 4.67 | 50.93 | 2.73 | 31.88 | 2.84 | 23.75 | 1.35 | 16.79 | 2.85 | 25.37 | 3.87 | 39.35 | GZMA |  |
|  |  |  |  |  |  |  |  | 2.29 | 19.00 | 6.17 | 65.58 | 4.57 | 44.44 | 4.06 | 30.73 | 0.83 | 12.25 | 8.88 | 68.05 | 6.05 | 61.01 | NKG7 |  |
|  |  |  |  |  |  |  |  | 0.33 | 7.36 | 0.50 | 10.70 | 0.71 | 16.21 | 0.46 | 6.98 | 0.69 | 14.58 | 0.53 | 10.12 | 0.51 | 10.14 | TNFRSF14 | Co_stim |
|  |  |  |  |  |  |  |  | 0.11 | 2.14 | 0.19 | 3.49 | 0.33 | 6.56 | 0.96 | 10.80 | 0.66 | 11.52 | 0.04 | 0.73 | 0.24 | 3.32 | CD28 |  |
|  |  |  |  |  |  |  |  | 1.30 | 19.00 | 1.29 | 18.60 | 0.88 | 18.40 | 1.08 | 11.96 | 1.38 | 19.61 | 0.34 | 6.34 | 1.08 | 17.14 | ICOS |  |
|  |  |  |  |  |  |  |  | 0.10 | 2.61 | 0.45 | 5.81 | 1.34 | 10.93 | 3.01 | 21.10 | 0.38 | 6.37 | 0.70 | 8.78 | 0.81 | 8.85 | TNFRSF9 |  |
|  |  |  |  |  |  |  |  | 0.78 | 15.91 | 0.25 | 4.42 | 0.19 | 5.28 | 0.17 | 2.49 | 0.98 | 18.26 | 0.08 | 1.59 | 0.11 | 2.21 | TNFRSF25 |  |
|  |  |  |  |  |  |  |  | 0.00 | 0.00 | 0.00 | 0.00 | 0.01 | 0.36 | 0.05 | 0.83 | 0.02 | 0.25 | 0.00 | 0.00 | 0.00 | 0.00 | FOXP3 | Treg |
|  |  |  |  |  |  |  |  | 0.81 | 12.83 | 0.05 | 0.93 | 0.08 | 2.19 | 0.26 | 3.65 | 0.37 | 6.74 | 0.15 | 2.56 | 0.14 | 2.30 | IL2RA |  |
|  |  |  |  |  |  |  |  | 0.69 | 12.35 | 0.03 | 0.47 | 0.03 | 0.55 | 0.48 | 4.82 | 0.10 | 1.84 | 0.39 | 7.20 | 0.02 | 0.28 | IKZF2 |  |

| cluster 10 | | cluster 9 | | cluster 8 | | cluster 7 | | cluster 6 | | cluster 5 | | cluster 4 | | cluster 3 | | cluster 2 | | cluster 1 | | cluster 0 | |  |  |
| --- | --- | --- | --- | --- | --- | --- | --- | --- | --- | --- | --- | --- | --- | --- | --- | --- | --- | --- | --- | --- | --- | --- | --- |
| avg.exp | pct.exp | avg.exp | pct.exp | avg.exp | pct.exp | avg.exp | pct.exp | avg.exp | pct.exp | avg.exp | pct.exp | avg.exp | pct.exp | avg.exp | pct.exp | avg.exp | pct.exp | avg.exp | pct.exp | avg.exp | pct.exp | Gene |  |
| 0.83 | 12.21 | 0.16 | 2.16 | 0.61 | 6.64 | 0.55 | 10.05 | 3.04 | 35.13 | 1.03 | 11.72 | 1.54 | 19.75 | 0.13 | 2.40 | 0.47 | 7.32 | 0.15 | 2.60 | 0.22 | 3.49 | CCR7 | Naive |
| 0.72 | 16.14 | 0.51 | 9.42 | 0.70 | 12.73 | 0.52 | 12.90 | 1.07 | 20.60 | 0.72 | 12.85 | 1.03 | 18.89 | 0.51 | 9.85 | 0.75 | 12.34 | 0.51 | 10.40 | 0.56 | 10.33 | TCF7 | Naive |
| 0.36 | 7.00 | 0.04 | 1.04 | 0.25 | 4.94 | 0.16 | 5.25 | 0.77 | 16.05 | 0.10 | 2.16 | 0.13 | 2.97 | 0.06 | 1.31 | 0.08 | 1.87 | 0.05 | 1.20 | 0.06 | 1.42 | LEF1 | Naive |
| 0.46 | 8.28 | 0.08 | 1.44 | 0.45 | 6.64 | 0.31 | 7.33 | 1.98 | 30.61 | 0.29 | 4.80 | 0.51 | 8.22 | 0.06 | 0.98 | 0.26 | 4.32 | 0.05 | 1.05 | 0.07 | 1.48 | SELL | Naive |
| 12.05 | 57.81 | 12.28 | 73.08 | 8.13 | 59.72 | 11.54 | 71.76 | 11.28 | 76.97 | 5.73 | 45.20 | 16.33 | 68.66 | 10.04 | 70.22 | 3.77 | 30.50 | 11.91 | 74.90 | 13.08 | 76.25 | IL7R | Naive |
| 7.71 | 75.66 | 8.97 | 74.60 | 5.33 | 58.95 | 7.60 | 73.17 | 5.35 | 59.81 | 5.61 | 60.15 | 12.83 | 82.18 | 6.67 | 68.91 | 11.47 | 80.81 | 8.19 | 77.43 | 9.60 | 78.58 | PTPRC | Naive |
| 0.42 | 9.39 | 0.13 | 2.56 | 0.23 | 3.86 | 0.16 | 4.43 | 0.70 | 13.52 | 0.37 | 6.69 | 0.65 | 12.18 | 0.09 | 1.86 | 0.56 | 9.84 | 0.10 | 2.15 | 0.12 | 2.52 | CD28 | TM |
| 0.05 | 1.20 | 0.00 | 0.08 | 0.02 | 0.31 | 0.01 | 0.14 | 0.03 | 0.40 | 0.03 | 0.80 | 0.13 | 2.43 | 0.00 | 0.04 | 0.01 | 0.17 | 0.00 | 0.07 | 0.01 | 0.12 | ADGRG1 | TM |
| 0.03 | 0.51 | 0.02 | 0.40 | 0.01 | 0.15 | 0.02 | 0.54 | 0.00 | 0.10 | 0.03 | 0.65 | 0.03 | 0.77 | 0.01 | 0.15 | 0.04 | 0.72 | 0.01 | 0.15 | 0.01 | 0.25 | FGFBP2 | TM |
| 4.95 | 42.10 | 3.05 | 33.95 | 3.42 | 32.18 | 4.16 | 39.73 | 0.61 | 7.75 | 4.12 | 40.85 | 1.39 | 18.37 | 4.31 | 40.99 | 8.97 | 67.90 | 4.82 | 47.61 | 3.27 | 35.20 | GZMA | Cyto |
| 1.27 | 11.10 | 8.96 | 55.59 | 1.71 | 16.05 | 5.12 | 38.96 | 0.29 | 2.87 | 2.42 | 19.12 | 4.16 | 19.68 | 8.06 | 51.69 | 2.28 | 17.73 | 13.15 | 69.16 | 9.48 | 59.81 | GZMB | Cyto |
| 10.93 | 34.33 | 4.93 | 30.19 | 12.08 | 38.97 | 1.91 | 17.15 | 2.15 | 12.64 | 12.60 | 43.55 | 3.38 | 24.18 | 11.66 | 49.31 | 5.74 | 32.22 | 12.11 | 54.34 | 1.40 | 15.11 | IFNG | Cyto |
| 5.20 | 42.19 | 5.34 | 56.07 | 4.66 | 44.44 | 5.74 | 55.02 | 0.63 | 7.89 | 7.05 | 57.05 | 4.10 | 34.95 | 5.30 | 59.22 | 12.26 | 74.75 | 5.64 | 63.57 | 5.62 | 58.01 | NKG7 | Cyto |
| 27.18 | 52.52 | 108.31 | 91.45 | 39.78 | 63.19 | 42.82 | 77.06 | 5.23 | 20.73 | 39.75 | 71.97 | 9.54 | 36.88 | 187.36 | 93.80 | 101.23 | 90.52 | 200.95 | 96.68 | 51.27 | 86.05 | CCL4 | Cyto |
| 22.24 | 70.88 | 51.69 | 98.64 | 37.05 | 82.25 | 52.42 | 93.21 | 9.16 | 31.83 | 36.75 | 89.31 | 16.37 | 69.50 | 59.07 | 99.10 | 49.54 | 97.43 | 60.17 | 99.20 | 49.51 | 98.68 | CCL5 | Cyto |
| 0.52 | 11.02 | 1.24 | 20.93 | 0.74 | 12.35 | 0.71 | 13.35 | 0.19 | 3.24 | 0.85 | 13.77 | 0.52 | 9.06 | 0.86 | 16.28 | 1.00 | 16.23 | 1.14 | 22.25 | 1.11 | 18.73 | LAG3 | TEX |
| 0.09 | 1.79 | 0.21 | 3.35 | 0.09 | 1.70 | 0.19 | 3.89 | 0.08 | 1.69 | 0.12 | 2.16 | 0.09 | 1.73 | 0.13 | 2.34 | 0.15 | 2.35 | 0.17 | 3.52 | 0.19 | 3.47 | HAVCR2 | TEX |
| 0.93 | 15.71 | 0.70 | 11.98 | 0.80 | 13.04 | 0.67 | 12.17 | 1.34 | 19.72 | 1.17 | 18.03 | 1.22 | 17.55 | 0.50 | 9.69 | 1.90 | 26.85 | 0.73 | 14.87 | 0.65 | 11.18 | TIGIT | TEX |
| 0.41 | 8.20 | 0.97 | 15.73 | 0.35 | 6.71 | 0.63 | 11.81 | 0.41 | 7.55 | 0.68 | 10.66 | 0.45 | 8.27 | 0.57 | 10.63 | 0.83 | 13.07 | 0.99 | 18.41 | 0.99 | 15.69 | PDCD1 | TEX |
| 0.18 | 3.67 | 0.23 | 4.31 | 0.10 | 1.70 | 0.21 | 4.98 | 0.78 | 11.29 | 0.14 | 2.34 | 0.36 | 6.19 | 0.10 | 1.88 | 0.32 | 4.93 | 0.26 | 4.78 | 0.20 | 3.51 | CTLA4 | TEX |
| 0.50 | 10.76 | 0.66 | 12.54 | 0.63 | 12.58 | 0.79 | 16.33 | 0.52 | 10.65 | 0.53 | 9.89 | 0.38 | 7.33 | 0.92 | 17.64 | 0.62 | 10.99 | 0.77 | 16.29 | 0.72 | 13.51 | ITGAE | TRM |
| 1.04 | 15.63 | 1.91 | 25.64 | 0.75 | 10.73 | 1.53 | 21.99 | 0.35 | 4.96 | 0.83 | 12.40 | 1.28 | 16.98 | 1.17 | 19.12 | 1.28 | 17.26 | 2.16 | 32.45 | 1.40 | 21.44 | CXCR6 | TRM |
| 24.31 | 73.53 | 21.01 | 84.66 | 31.43 | 84.34 | 16.24 | 83.08 | 12.77 | 66.99 | 20.59 | 75.31 | 7.48 | 61.44 | 37.66 | 94.85 | 17.11 | 78.12 | 33.22 | 95.35 | 13.55 | 80.43 | CD69 | TRM |
| 0.02 | 0.51 | 0.01 | 0.24 | 0.01 | 0.23 | 0.02 | 0.27 | 0.08 | 1.69 | 0.00 | 0.06 | 0.01 | 0.20 | 0.01 | 0.22 | 0.00 | 0.10 | 0.00 | 0.10 | 0.01 | 0.14 | FOXP3 | Treg |
| 0.10 | 2.48 | 0.09 | 1.68 | 0.06 | 1.39 | 0.09 | 2.53 | 0.35 | 6.44 | 0.07 | 1.24 | 0.17 | 2.92 | 0.07 | 1.40 | 0.03 | 0.42 | 0.06 | 1.49 | 0.05 | 1.04 | IL2RA | Treg |
| 0.16 | 3.16 | 0.02 | 0.48 | 0.06 | 1.16 | 0.06 | 1.22 | 0.22 | 4.55 | 0.08 | 1.54 | 0.06 | 1.11 | 0.03 | 0.59 | 0.14 | 2.38 | 0.02 | 0.49 | 0.02 | 0.45 | IKZF2 | Treg |
|  |  |  |  |  |  |  |  |  |  |  |  |  |  |  |  |  |  |  |  |  |  |  |  |
|  |  |  |  |  |  |  |  | cluster 17 | | cluster 16 | | cluster 15 | | cluster 14 | | cluster 13 | | cluster 12 | | cluster 11 | |  |  |
|  |  |  |  |  |  |  |  | avg.exp | pct.exp | avg.exp | pct.exp | avg.exp | pct.exp | avg.exp | pct.exp | avg.exp | pct.exp | avg.exp | pct.exp | avg.exp | pct.exp | Gene |  |
|  |  |  |  |  |  |  |  | 1.13 | 14.96 | 0.32 | 4.19 | 0.62 | 10.75 | 1.61 | 10.96 | 1.87 | 20.83 | 0.11 | 1.83 | 0.35 | 4.88 | CCR7 | Naive |
|  |  |  |  |  |  |  |  | 1.35 | 22.09 | 0.67 | 11.63 | 0.99 | 19.85 | 0.89 | 12.79 | 0.73 | 14.71 | 0.85 | 16.46 | 0.54 | 9.59 | TCF7 | Naive |
|  |  |  |  |  |  |  |  | 0.07 | 1.19 | 0.06 | 0.70 | 0.16 | 3.64 | 0.12 | 1.99 | 0.12 | 2.57 | 0.29 | 5.49 | 0.02 | 0.46 | LEF1 | Naive |
|  |  |  |  |  |  |  |  | 0.48 | 6.41 | 0.03 | 0.70 | 0.22 | 4.19 | 0.34 | 4.32 | 0.22 | 4.17 | 0.15 | 2.80 | 0.06 | 1.11 | SELL | Naive |
|  |  |  |  |  |  |  |  | 26.47 | 84.32 | 6.07 | 49.53 | 11.87 | 68.85 | 12.34 | 47.51 | 19.18 | 87.25 | 6.64 | 47.93 | 8.13 | 57.24 | IL7R | Naive |
|  |  |  |  |  |  |  |  | 4.73 | 57.72 | 6.42 | 68.37 | 9.84 | 81.60 | 12.52 | 65.78 | 5.59 | 62.25 | 8.52 | 72.56 | 6.35 | 63.50 | PTPRC | Naive |
|  |  |  |  |  |  |  |  | 0.11 | 2.14 | 0.19 | 3.49 | 0.33 | 6.56 | 0.96 | 10.80 | 0.66 | 11.52 | 0.04 | 0.73 | 0.24 | 3.32 | CD28 | TM |
|  |  |  |  |  |  |  |  | 0.04 | 0.71 | 0.00 | 0.00 | 0.04 | 0.91 | 0.08 | 1.00 | 0.01 | 0.25 | 0.01 | 0.12 | 0.03 | 0.65 | ADGRG1 | TM |
|  |  |  |  |  |  |  |  | 0.00 | 0.00 | 0.02 | 0.23 | 0.00 | 0.00 | 0.03 | 0.33 | 0.01 | 0.12 | 0.02 | 0.24 | 0.02 | 0.37 | FGFBP2 | TM |
|  |  |  |  |  |  |  |  | 1.05 | 10.21 | 4.67 | 50.93 | 2.73 | 31.88 | 2.84 | 23.75 | 1.35 | 16.79 | 2.85 | 25.37 | 3.87 | 39.35 | GZMA | Cyto |
|  |  |  |  |  |  |  |  | 0.88 | 7.60 | 5.55 | 39.30 | 4.06 | 31.33 | 2.44 | 8.97 | 0.53 | 4.41 | 7.19 | 42.20 | 7.20 | 46.73 | GZMB | Cyto |
|  |  |  |  |  |  |  |  | 1.38 | 8.55 | 23.83 | 60.23 | 3.42 | 25.32 | 5.47 | 19.77 | 4.46 | 17.65 | 5.03 | 30.00 | 8.09 | 38.06 | IFNG | Cyto |
|  |  |  |  |  |  |  |  | 2.29 | 19.00 | 6.17 | 65.58 | 4.57 | 44.44 | 4.06 | 30.73 | 0.83 | 12.25 | 8.88 | 68.05 | 6.05 | 61.01 | NKG7 | Cyto |
|  |  |  |  |  |  |  |  | 32.43 | 29.22 | 107.61 | 92.09 | 44.59 | 70.31 | 21.36 | 43.36 | 11.07 | 27.45 | 92.99 | 78.90 | 40.76 | 82.49 | CCL4 | Cyto |
|  |  |  |  |  |  |  |  | 10.06 | 38.00 | 53.37 | 97.44 | 27.34 | 83.06 | 15.40 | 54.49 | 11.92 | 50.12 | 54.97 | 90.49 | 46.57 | 96.41 | CCL5 | Cyto |
|  |  |  |  |  |  |  |  | 0.13 | 2.61 | 1.18 | 18.37 | 0.81 | 14.94 | 0.46 | 6.81 | 0.37 | 6.25 | 0.74 | 13.78 | 1.05 | 18.89 | LAG3 | TEX |
|  |  |  |  |  |  |  |  | 0.45 | 7.60 | 0.11 | 2.33 | 0.14 | 3.10 | 0.17 | 1.50 | 0.08 | 1.72 | 0.29 | 5.12 | 0.11 | 2.30 | HAVCR2 | TEX |
|  |  |  |  |  |  |  |  | 0.15 | 3.33 | 0.95 | 13.49 | 0.98 | 16.94 | 1.90 | 21.59 | 0.21 | 4.78 | 0.96 | 15.00 | 0.69 | 12.35 | TIGIT | TEX |
|  |  |  |  |  |  |  |  | 0.07 | 0.95 | 0.55 | 10.00 | 0.75 | 15.30 | 0.31 | 4.82 | 0.54 | 10.66 | 0.14 | 2.20 | 0.64 | 10.32 | PDCD1 | TEX |
|  |  |  |  |  |  |  |  | 0.10 | 2.38 | 0.08 | 1.16 | 0.31 | 5.10 | 0.89 | 9.14 | 0.39 | 6.13 | 0.09 | 1.83 | 0.08 | 1.47 | CTLA4 | TEX |
|  |  |  |  |  |  |  |  | 0.45 | 10.21 | 0.65 | 13.72 | 0.74 | 16.76 | 0.34 | 5.32 | 0.50 | 10.66 | 0.75 | 14.39 | 0.51 | 9.68 | ITGAE | TRM |
|  |  |  |  |  |  |  |  | 0.48 | 8.55 | 1.02 | 17.67 | 1.21 | 20.58 | 1.14 | 10.47 | 0.90 | 14.09 | 1.44 | 21.46 | 1.13 | 16.50 | CXCR6 | TRM |
|  |  |  |  |  |  |  |  | 23.39 | 78.15 | 43.61 | 89.77 | 16.73 | 77.41 | 9.54 | 48.34 | 22.33 | 79.41 | 23.37 | 86.46 | 17.19 | 78.43 | CD69 | TRM |
|  |  |  |  |  |  |  |  | 0.00 | 0.00 | 0.00 | 0.00 | 0.01 | 0.36 | 0.05 | 0.83 | 0.02 | 0.25 | 0.00 | 0.00 | 0.00 | 0.00 | FOXP3 | Treg |
|  |  |  |  |  |  |  |  | 0.81 | 12.83 | 0.05 | 0.93 | 0.08 | 2.19 | 0.26 | 3.65 | 0.37 | 6.74 | 0.15 | 2.56 | 0.14 | 2.30 | IL2RA | Treg |
|  |  |  |  |  |  |  |  | 0.69 | 12.35 | 0.03 | 0.47 | 0.03 | 0.55 | 0.48 | 4.82 | 0.10 | 1.84 | 0.39 | 7.20 | 0.02 | 0.28 | IKZF2 | Treg |

Table S11. Antibody specifications.

| Antibody | Supplier | Catalog number | Dilution ratio |
| --- | --- | --- | --- |
| CD14 | Cell Signaling Technology (CST) | 75181 | 1: 100 |
| CD16 | Cell Signaling Technology (CST) | 24326 | 1: 100 |
| CD8 | Cell Signaling Technology (CST) | 85336 | 1: 100 |
| Dylight488 secondary antibody | Cell Signaling Technology (CST) | 12935 | 1: 400 |

Reference:

[1] Zheng GX, Terry JM, Belgrader P, et al. Massively parallel digital transcriptional profiling of single cells. Nat Commun. 2017;8:14049.

[2] Hirz T, Mei S, Sarkar H, et al. Dissecting the immune suppressive human prostate tumor microenvironment via integrated single-cell and spatial transcriptomic analyses. *Nat Commun*. 2023;14(1):663.

[3] Ma L, Hernandez MO, Zhao Y, et al. Tumor Cell Biodiversity Drives Microenvironmental Reprogramming in Liver Cancer. *Cancer Cell*. 2019;36(4):418-430.e6.

[4] Vanlandewijck M, He L, Mäe MA, et al. A molecular atlas of cell types and zonation in the brain vasculature. *Nature*. 2018;554(7693):475-480.

[5] Huang FW, Song H, Weinstein HN, et al. Club-like cells in proliferative inflammatory atrophy of the prostate. *J Pathol*. 2023;261(1):85-95.

[6] Yan Q, Wang M, Xia H, et al. Single-cell RNA-sequencing technology demonstrates the heterogeneity between aged prostate peripheral and transitional zone. Clin Transl Med. 2022;12(10):e1084.

[7] Joseph DB, Henry GH, Malewska A, et al. Single-cell analysis of mouse and human prostate reveals novel fibroblasts with specialized distribution and microenvironment interactions. J Pathol. 2021;255(2):141-154.

[8] Öhlund D, Handly-Santana A, Biffi G, et al. Distinct populations of inflammatory fibroblasts and myofibroblasts in pancreatic cancer. J Exp Med. 2017;214(3):579-596.

[9] Dominguez CX, Müller S, Keerthivasan S, et al. Single-Cell RNA Sequencing Reveals Stromal Evolution into LRRC15+ Myofibroblasts as a Determinant of Patient Response to Cancer Immunotherapy. Cancer Discov. 2020;10(2):232-253.

[10] Ak Ç, Sayar Z, Thibault G, et al. Multiplex imaging of localized prostate tumors reveals altered spatial organization of AR-positive cells in the microenvironment. iScience. 2024;27(9):110668.
